# Genome Sequencing Highlights the Dynamic Early History of Dogs

**DOI:** 10.1371/journal.pgen.1004016

**Published:** 2014-01-16

**Authors:** Adam H. Freedman, Ilan Gronau, Rena M. Schweizer, Diego Ortega-Del Vecchyo, Eunjung Han, Pedro M. Silva, Marco Galaverni, Zhenxin Fan, Peter Marx, Belen Lorente-Galdos, Holly Beale, Oscar Ramirez, Farhad Hormozdiari, Can Alkan, Carles Vilà, Kevin Squire, Eli Geffen, Josip Kusak, Adam R. Boyko, Heidi G. Parker, Clarence Lee, Vasisht Tadigotla, Adam Siepel, Carlos D. Bustamante, Timothy T. Harkins, Stanley F. Nelson, Elaine A. Ostrander, Tomas Marques-Bonet, Robert K. Wayne, John Novembre

**Affiliations:** 1 Department of Ecology and Evolutionary Biology, University of California, Los Angeles, Los Angeles, California, United States of America; 2 Department of Biological Statistics and Computational Biology, Cornell University, Ithaca, New York, United States of America; 3 CIBIO-UP, University of Porto, Vairão, Portugal; 4 ISPRA, Ozzano dell'Emilia, Italy; 5 Key Laboratory of Bioresources and Ecoenvironment, Sichuan University, Chengdu, China; 6 Department of Measurement and Information Systems, Budapest University of Technology and Economics, Budapest, Hungary; 7 Institut de Biologia Evolutiva (CSIC-Univ Pompeu Fabra), Barcelona, Spain; 8 National Institutes of Health/NHGRI, Bethesda, Maryland, United States of America; 9 Department of Computer Science, University of California, Los Angeles, Los Angeles, California, United States of America; 10 Bilkent University, Ankara, Turkey; 11 Estación Biológia de Doñana EBD-CSIC, Sevilla, Spain; 12 Department of Human Genetics, University of California, Los Angeles, Los Angeles, California, United States of America; 13 Department of Zoology, Tel Aviv University, Tel Aviv, Israel; 14 University of Zagreb, Zagreb, Croatia; 15 Department of Veterinary Medicine, Cornell University, Ithaca, New York, United States of America; 16 Life Technologies, Foster City, California, United States of America; 17 Stanford School of Medicine, Stanford, California, United States of America; 18 Institució Catalana de Recerca i Estudis Avançats (ICREA). 08010, Barcelona, Spain; Uppsala University, Sweden

## Abstract

To identify genetic changes underlying dog domestication and reconstruct their early evolutionary history, we generated high-quality genome sequences from three gray wolves, one from each of the three putative centers of dog domestication, two basal dog lineages (Basenji and Dingo) and a golden jackal as an outgroup. Analysis of these sequences supports a demographic model in which dogs and wolves diverged through a dynamic process involving population bottlenecks in both lineages and post-divergence gene flow. In dogs, the domestication bottleneck involved at least a 16-fold reduction in population size, a much more severe bottleneck than estimated previously. A sharp bottleneck in wolves occurred soon after their divergence from dogs, implying that the pool of diversity from which dogs arose was substantially larger than represented by modern wolf populations. We narrow the plausible range for the date of initial dog domestication to an interval spanning 11–16 thousand years ago, predating the rise of agriculture. In light of this finding, we expand upon previous work regarding the increase in copy number of the amylase gene (*AMY2B*) in dogs, which is believed to have aided digestion of starch in agricultural refuse. We find standing variation for amylase copy number variation in wolves and little or no copy number increase in the Dingo and Husky lineages. In conjunction with the estimated timing of dog origins, these results provide additional support to archaeological finds, suggesting the earliest dogs arose alongside hunter-gathers rather than agriculturists. Regarding the geographic origin of dogs, we find that, surprisingly, none of the extant wolf lineages from putative domestication centers is more closely related to dogs, and, instead, the sampled wolves form a sister monophyletic clade. This result, in combination with dog-wolf admixture during the process of domestication, suggests that a re-evaluation of past hypotheses regarding dog origins is necessary.

## Author Summary

The process of dog domestication is still poorly understood, largely because no studies thus far have leveraged deeply sequenced whole genomes from wolves and dogs to simultaneously evaluate support for the proposed source regions: East Asia, the Middle East, and Europe. To investigate dog origins, we sequence three wolf genomes from the putative centers of origin, two basal dog breeds (Basenji and Dingo), and a golden jackal as an outgroup. We find that none of the wolf lineages from the hypothesized domestication centers is supported as the source lineage for dogs, and that dogs and wolves diverged 11,000–16,000 years ago in a process involving extensive admixture and that was followed by a bottleneck in wolves. In addition, we investigate the amylase (*AMY2B*) gene family expansion in dogs, which has recently been suggested as being critical to domestication in response to increased dietary starch. We find standing variation in *AMY2B* copy number in wolves and show that some breeds, such as Dingo and Husky, lack the *AMY2B* expansion. This suggests that, at the beginning of the domestication process, dogs may have been characterized by a more carnivorous diet than their modern day counterparts, a diet held in common with early hunter-gatherers.

## Introduction

Gray wolves have been dominant predators across Eurasia and North America, often exerting top-down impacts on the ecological communities they inhabit [1], [2]. As humans expanded out of Africa into Eurasia, they came into contact with gray wolves and, through a complex and poorly understood process, dogs emerged as the first human companion species and the only large carnivore to ever be domesticated. Archaeological evidence provides partial clues about dog origins. For example, dog-like canids first appear in the fossil record as early as 33,000 years ago in Siberia [3]. However, it is not clear if these proto-dog fossils are ancestral to living dogs or instead represent failed domestication attempts or simply morphologically distinct wolves [3]. Similarly, the geographic origin of dogs is uncertain, with distinct lines of evidence supporting Southeast Asia, the Middle East, and Europe as potential domestication centers, and ruling out Africa, Australia, and North America [4]–[10]. Nonetheless, several recent studies have begun to illuminate the genetic basis of traits that changed during dog domestication and breed formation, advancing the general understanding of how genetic mechanisms shape phenotypic trait diversity [11]–[14]. For example, a recent study found an increase in copy number of the amylase gene (*AMY2B*) during dog domestication suggesting adaptation to starch-rich diets [15]. Given the unique behavioral adaptations of dogs, including docility and the ability to form social bonds with humans [16], comparative genomics analyses of dogs and wolves holds great promise for identifying genetic loci involved in complex behavioral traits [14]. However, the demographic context of selection must first be understood to determine how it may have affected patterns of genetic divergence between dogs and wolves.

To advance the understanding of dog origins and genetic changes early in dog domestication, we sequenced the genomes of six canid individuals, including three wolves (*Canis lupus*), an Australian Dingo, a Basenji and a golden jackal (*Canis aureus*).

**Figure 1:**
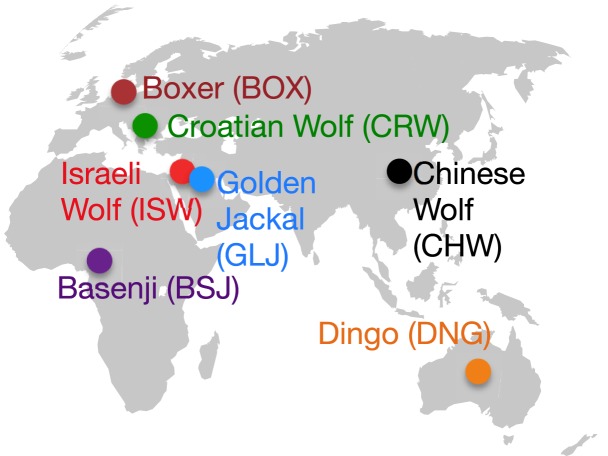
Geographic distribution of sampled lineages.

The three wolves were chosen to represent the broad regions of Eurasia where domestication is hypothesized to have taken place (Europe, the Middle East, and East/Southeast Asia) [6], and specifically, were sampled from Croatia, Israel, and China (Figure 1). The Dingo and Basenji represent divergent lineages relative to the reference Boxer genome [10] and maximize the opportunity to capture distinct alleles present in the earliest dogs. These lineages are also geographically distinct, with modern Basenjis tracing their history to hunting dogs of western Africa, while Dingoes are free-living semi-feral dogs of Australia that arrived there at least 3,500 years ago (Figure 1) [17]. As a result of their geographic isolation, the natural range of wolves has never extended as far south as the geographic sources for these two dog lineages [6], thus they are less likely to have overlapped and admixed with wolves in the recent past. Sequencing the golden jackal in principle allows us to infer the ancestral state of variants arising in dogs and wolves (Text S1, S2), though in practice this was complicated by the observation of wolf-jackal admixture (see below). For some analyses, we also leverage data from a companion study of 12 additional dog breeds (Text S1).

We chose to sequence a small number of individual genomes to high coverage, rather than larger numbers of (pooled) individuals at low coverage, to take advantage of recently developed demography inference methods based on small numbers of high quality genomes [18]–[20]. These methods allowed us to disentangle the effects of incomplete lineage sorting (ILS)–the discordance from the population phylogeny at individual loci resulting from deep coalescence–and post-divergence gene flow, which pose a particular challenge in analysis of such recently diverged species as dogs and wolves [21]. Combining the results of multiple complementary methods provided us with an integrated, robust view of the shared history of dogs and wolves, including population divergence times, ancestral population sizes, and rates of gene flow. Using polymorphism data from 10 million single-nucleotide variant sites, we investigated: 1) the size of the ancestral wolf population at the time of wolf/dog divergence; 2) the geographic origins and timing of dog domestication; 3) post-divergence admixture between dogs and wolves; and 4) lineage-specific characteristics of the recently discovered dog-specific *AMY2B* expansion [15].

## Results

### Individual-level genome sequences

For each of the six samples, we generated high-quality genome sequences. Cumulative coverage was 72× for the wolves (24× average per individual), 38× coverage for the two dogs (19× average per individual), and 24× for the golden jackal, for a total of 335 Gb of uniquely aligned sequence from 11.2 billion reads (Table S1). Surveys of wolf genetic diversity to date have been limited to shotgun sequencing with incomplete genomic coverage [22], small numbers of sequence loci [23], limited pooled sequencing (6× average from a pool of 12 wolves, 30× average from a pool of 60 dogs) [15] or lower coverage sequencing (9–11× coverage of 4 wolves, 9–14× of 7 dogs) [24].

**Figure 2:**
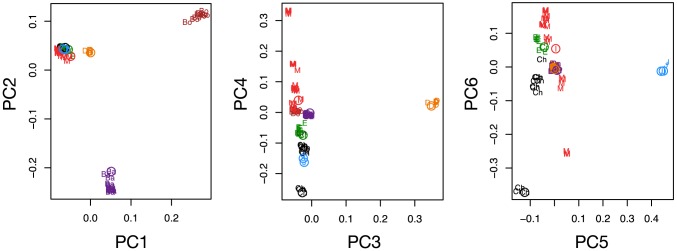
Comparison of next generation sequencing with array typed samples, and historical changes in effective population size. PCA plot of next-generation sequencing (NGS) samples generated in this study (open circles) along with corresponding samples genotyped on the Affymetrix canid array [10] (colors and two letter codes: red M = Mid-East Wolf, green E = European Wolf, black Ch = Chinese Wolf, purple Ba = Basenji, brown Bo = Boxer, orange D = Dingo, cyan J = Golden Jackal).

Our analyses draw on 10,265,254 high quality variants detected by our genotyping pipeline (Text S3, S4, S5), of which 6,970,672 were at genomic positions with no missing data for any lineage (Tables S2, S3). We estimate genotype error rates to be very low based on comparison to genotype calls from genotyping arrays (e.g. heterozygote discordance rates of 0.01–0.04%, Tables S4, S5, Text S5). Further, PCA on the intersection of sequencing and genotyping array variants show the novel samples cluster appropriately, suggesting batch effects due to technology have been minimized (Figure 2, Text S5).

### Ancestral population sizes of dogs and wolves

**Figure 3:**
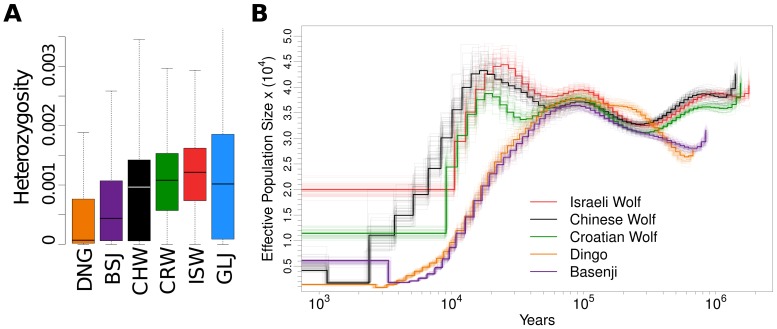
Heterozygosity and historical changes in effective population size. (A) Box plots of heterozygosity measured in 5000 100 kb windows for each sample. (B) Reconstruction of historical patterns of effective population size (N_e_) for individual genome sequences. Based upon the genomic distribution of heterozygous sites using the pairwise sequential Markovian coalescent (PSMC) method of Li and Durbin 2011 [20]. Time scale on the x-axis is calculated assuming a mutation rate of 1×10^−8^ per generation (see Text S8); estimates from the full data and 50 bootstraps are depicted by darker and lighter lines, respectively.

Genome-wide patterns of heterozygosity provide useful information on long-term effective population sizes. The mean heterozygosity rates (per nucleotide position) observed in the genome sequences of the Basenji and Dingo were 9×10^−4^ and 6×10^−4^, respectively (Figure 3A, Table S6), consistent with a rate of 6×10^−4^ previously observed in modern dog breeds [22], and considerably smaller than the rates observed in the three wolf genomes (1.2×10^−3^–1.6×10^−3^). This twofold reduction in heterozygosity observed in dogs relative to wolves can be superficially interpreted to reflect a relatively weak two-fold reduction in effective population size of dogs relative to their ancestors, assuming that genetic variation in modern wolves is representative of the ancestral population.

To better understand the changes in ancestral population sizes that influenced dogs and wolves, we employed the pairwise sequential Markovian coalescent (PSMC) method [20]. This method infers ancestral effective population sizes (*N_e_*) over time, based on a probabilistic model of coalescence with recombination and changes in heterozygosity rates along a single diploid genome. We applied PSMC to each of the five genomes (Figure 3B, Text S8) and converted the mutation-scaled estimates of time (to years) and population size (to numbers of individuals) by assuming an average mutation rate per generation of μ = 1×10^−8^ and an average generation time of three years [22],[25] (see **Discussion**). The inferred tracks of ancestral *N_e_* in dogs show a population decline of at least 16-fold over the past 50 thousand years, from greater than 32,000 individuals (ancestral *N_e_* for Basenji lineage: 32,100–35,500; for Dingo lineage: 32,500–37,400 95% bootstrap CI) to less than 2,000 individuals (Basenji lineage: 1640–1980 at 4,000 years ago; Dingo lineage: 704–1042 at 3,000 years ago). Interestingly, wolves also show a considerable, yet milder, 3-fold reduction in effective population size to present estimates between 10,000 and 17,000 for the three wolf samples. For clarity, we note that with PSMC the population size trajectories are effective sizes for the lineages that eventually lead to the canid samples as they are known today (e.g. as Basenji or as Dingo) and that looking backwards in time eventually trace back to the common ancestral lineage of dogs and wolves. Our observations do not appear to be biased by very recent inbreeding in dogs and wolves, as we found that runs of homozygosity do not affect our inferences of ancestral *N_e_* (Text S8). These results indicate the ancestral wolf population from which dogs were domesticated was considerably larger than estimated from current levels of diversity in wolves and suggest that simple comparisons of nucleotide diversity in present-day dogs and wolves lead to substantial underestimates of the severity of the bottleneck in dogs.

### Phylogenetic relationships and admixture between dogs and wolves

**Figure 4:**
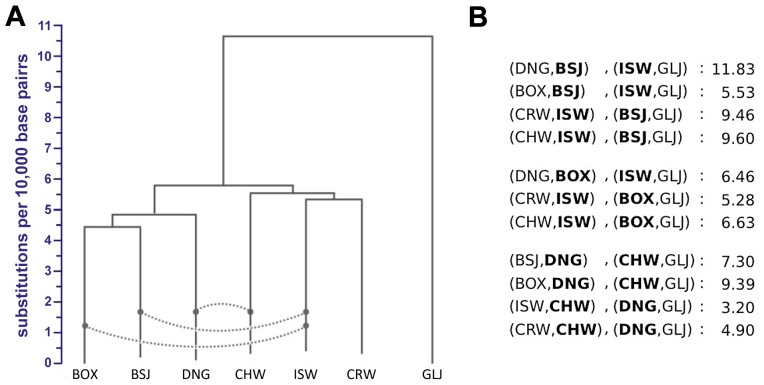
Neighbor-joining tree and admixture signatures from ABBA/BABA tests. (A) NJ tree constructed from genome-wide pairwise divergence, calculated using equation E8.1 in . All nodes have 100% bootstrap support. Dashed lines indicate admixture edges that were statistically significant in ABBA/BABA tests. (B) ABBA/BABA tests with significant Z-scores (values ≥3 are significant). All comparisons made are shown in Table S11. For each row, boldfaced labels indicate admixing lineages.

Individual genome sequences include valuable information about phylogenetic relationships between our samples. However, interpretation of these phylogenetic signals is challenging due to the possibility of post-divergence gene flow between dogs and wolves, as well as ILS, which is an expected consequence of the large ancestral population sizes inferred by PSMC. Indeed, we observed predominant ancestral polymorphism in our data: for variant sites with no missing data, and where variants were observed in dogs or wolves, 32.0% of variant sites were shared across dogs and wolves, 47.3% were private to wolves, 20.2% were private to dogs, and only 0.5% were fixed between dogs and wolves (Table S3). Pairwise sequence divergence captures mean coalescent times that are robust to both ILS and moderate levels of gene flow (see below). Thus, to provide accurate estimates of phylogeny given these demographic processes, we constructed a neighbor-joining (NJ) tree from a conservative estimator of genome-wide pairwise sequence divergence for all pairs in our seven genomes, including the Boxer reference and using the golden jackal as an outgroup (Figure 4A, Text S8, Table S7). Bootstrap support for all nodes was 100%, with dogs and wolves recovered as monophyletic sister clades. Surprisingly, the Boxer reference is only slightly more divergent from the three wolf genomes than it is from the two dog genomes. To evaluate the robustness of our phylogenetic inference, we also constructed a NJ tree using an estimator of sequence divergence for which all possible mismatches between alleles from a pair of individuals are counted (Table S8). The consensus tree based on this metric places the Chinese wolf at a position sister to a clade of our other wolf and dog samples (Figure S1), but the bootstrap support for this relationship is low (54%), suggesting poorer resolution with this estimator. Importantly, both approaches and additional phylogenetic analyses strongly support the hypothesis of dogs forming a distinct clade (Text S8, Tables S9, S10).

One important factor that could complicate inference of divergence between dogs and wolves is post-divergence gene flow. To examine admixture in our sampled genomes, we employed the nonparametric ‘ABBA-BABA’ test for gene flow between two divergent populations, such as humans and Neandertals [26], from individual genome sequences. This method tallies site patterns for four taxa, compares them to those expected under an assumed phylogeny and then uses this comparison to identify significant pattern asymmetries that cannot be explained by ILS or sequencing errors. We applied this test to all dog-wolf sample pairs, using the golden jackal as an outgroup and one of the other four samples as an additional ingroup (Text S8). We found significant evidence of admixture for three population pairs: Israeli wolf and Basenji, Chinese wolf and Dingo, and Israeli wolf and Boxer (Figure 4B, see also Table S11). Care should be taken in interpreting these results, as the detected admixture signals may reflect gene flow between lineages ancestral to our contemporary samples. The signal for Chinese wolf and Dingo likely represents ancient admixture in Eastern Eurasia, and the signal observed for Israeli wolf, Basenji, and Boxer likely represents ancestral admixture that occurred in western Eurasia. The resulting phylogeny with admixture edges (Figure 4A) is used as the starting point for a more comprehensive examination of joint demographic model for dogs and wolves.

### A complete demographic model for dogs and wolves

We next inferred a complete demographic model for dogs and wolves, including population divergence times, ancestral population sizes, and rates of post-divergence gene flow by jointly analyzing all seven genomes using the Generalized Phylogenetic Coalescent Sampler (*G-PhoCS*) [19], a recently developed Bayesian demographic inference method. The method is based on a full coalescent-based probabilistic model that considers both ILS (by modeling ancestral population size) and post-divergence gene flow (by allowing lineages to migrate between populations through designated migration bands). *G-PhoCS* conditions its inference on a given population phylogeny, and uses information on local genealogies at a large number of short, unlinked, neutrally evolving loci to generate samples of demographic parameters from an approximate posterior distribution. We applied *G-PhoCS* to a multiple sequence alignment of the six genomes and Boxer reference in 16,434 carefully filtered putative neutral autosomal loci using the NJ tree to indicate the topology of the population phylogeny (Text S9, see discussion on alternative topologies below).

Initially, we considered various migration bands with significant signatures of gene flow (Text S9). We found evidence of bi-directional gene flow between Israeli wolf and Basenji, as well as Chinese wolf and Dingo, consistent with our findings from the non-parametric ABBA-BABA test. Interestingly, the joint analysis of all genomes indicated that admixture inferred by the ABBA-BABA test for the Israeli wolf and the Boxer is likely a result of gene flow from a population ancestral to Basenji into a population ancestral to Israeli wolves. We base this conclusion on the observation that there is no significant signature of admixture between Boxer and Israeli wolf in the ABBA-BABA test or the *G-PhoCS* inference when Basenji is also included in the analysis. Using *G-PhoCS* we were also able to examine signatures of admixture in the jackal outgroup, which cannot be detected using the ABBA-BABA test, and found significant gene flow between the golden jackal and the Israeli wolf, as well as the population ancestral to all dog and wolf samples.

**Figure 5:**
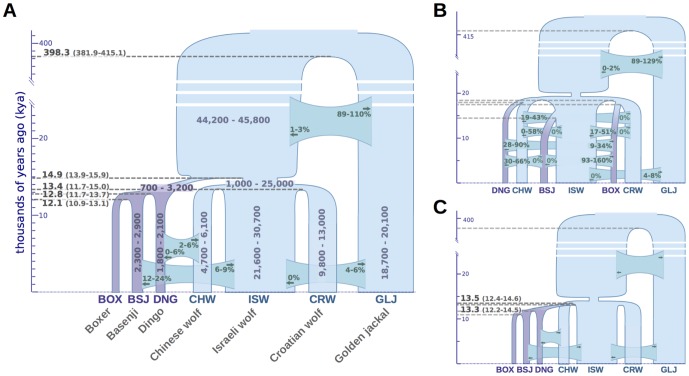
Demographic model of domestication. Divergence times, effective population sizes (*N_e_*), and post-divergence gene flow inferred by *G-PhoCS* in joint analysis of the Boxer reference genome, and the sequenced genomes of two basal dog breeds, three wolves, and a golden jackal. The width of each population branch is proportional to inferred population size, and stated ranges of parameter estimates indicate 95% Bayesian credible intervals. Horizontal gray dashed lines indicate timing of lineage divergences, with associated means in bold, and 95% credible intervals in parentheses. Migration bands are shown in green with associated values indicating estimates of total migration rates, which equal the probability that a lineage will migrate through the band during the time period when the two populations co-occur. Panels show parameter estimates for (A) the population tree best supported by genome-wide sequence divergence (Fig. 4A) (B) a regional domestication model, and (C) a single wolf lineage origin model in which dogs diverged most recently from the Israeli wolf lineage (similar star-like divergences are found assuming alternative choices for the single wolf ancestor. Estimated divergence times and effective population sizes are calibrated assuming an average mutation rate of 1×10^−8^ substitutions per generation and an average generation time of three years. See Text S9 and Table S12 for details.

Our divergence time estimates imply that dogs and wolves diverged 14.9 thousand years ago (kya) with 13.9–15.9 kya Bayesian 95% credible interval (CI), assuming an average mutation rate per generation of μ = 1×10^−8^ and three years per generation (Figure 5A). Divergence times between wolf populations were tightly clustered at 13.4 kya (11.7–15.1 kya), and divergence between dogs was estimated to have occurred slightly more recently, at 12.8 kya (11.8–13.7 kya; divergence of Dingo) and 12.1 kya (10.9–13.1 kya; divergence between Boxer and Basenji). Interestingly, we inferred a divergence time of 398 kya (382–415 kya) between the golden jackal and the population ancestral to dogs and wolves, which is considerably more recent than previously reported [27]. To validate this finding, we ensured that our estimates appropriately account for ancestral gene flow into the golden jackal population (Text S9) and validated the position of our sample within the golden jackal lineage by comparing polymorphism data from that genome to a larger panel of wolves and jackals (Text S5, S11).

*G-PhoCS* produced estimates of ancestral effective population sizes compatible with the ones inferred by PSMC, with a large effective population size of 45,000 individuals (44,200–44,800) for the population ancestral to dogs and wolves, followed by a 22-fold reduction to 2,000 individuals (700–3,200) in the population ancestral to all dogs, and a more moderate 3.6-fold reduction to 12,600 individuals (1,000–25,000) in the population ancestral to all wolves. As with our inferences based on PSMC, we estimate a far more severe domestication bottleneck than previously reported [22], [23].

The main discrepancy between PSMC and *G-PhoCS* concerns the timing of these changes. *G-PhoCS* associates this reduction in *N_e_* with the divergence between dogs and wolves at around 15 kya, whereas PSMC infers a gradual population decline starting as early as 50 kya (Figure 3B). As PSMC is based upon the density of heterozygous sites within the genome sequence of an individual, it does not directly infer divergence times. However, one can informally estimate them as the points when *N_e_* trajectories that are overlapping diverge moving forward in time towards the present. The discrepancy between *G-PhoCS* and PSMC reflects the distinct models used by these methods: *G-PhoCS* assumes a constant population size for every branch of the phylogeny, which prevents it from characterizing gradual changes in population size, whereas PSMC tends to produce smoothed traces of ancestral *N_e_*, which may limit its ability to capture rapid population bottlenecks. To test which of the inferred models has a better fit to the data, we simulated data under both models, and then used each method to analyze the data simulated under the model inferred by the other method (Text S8, S9). These two reciprocal tests indicated that the early and gradual population decline inferred by PSMC is compatible with a more recent dramatic reduction (Text S8, Figure S2), and that divergence time estimates of *G-PhoCS* were not compromised by its inability to model gradual changes in *N_e_* (Figure S3). Both results support the demographic model inferred by *G-PhoCS*, which has a relatively recent divergence between dogs and wolves followed by a dramatic reduction in population size. We additionally validated the robustness of our demographic parameter estimates under the set of loci chosen for the analysis as well as assumptions made on intra-locus recombination (Text S9).

### Alternative models for dog domestication

The demographic model we inferred using *G-PhoCS* reflects the population phylogeny estimated in the NJ analysis. To validate the robustness of our inference to this assumption, we considered a series of alternative topologies that correspond to plausible scenarios of the shared histories dogs and wolves. When we assume a model in which each dog population originated from the wolf population corresponding to its geographic origin (a model of regional domestication, e.g. Figure 5B), *G-PhoCS* infers extremely large rates of post-divergence gene flow between dogs and between wolves. For instance, the total rate of gene flow from Basenji to Boxer is inferred to be m^tot^ = 1.24 (0.93–1.59, 95% Bayesian CI), implying a probability near 100% for any Boxer lineage to have migrated from a population ancestral to Basenji. Total rates above 30% were inferred for additional migration bands, such as Basenji-to-Dingo (0.47), Croatian-to-Israeli wolf (0.33), and Croatian-to-Chinese wolf (0.33) (Figure S4). In terms of the number of migrants per generation (4*N_e_*m), these estimates translate into 0.26 (CI: 0.15–0.38), 4.48 (CI: 2.52–6.36), and 0.89 (CI: 0.56–1.23), reflecting large amounts of gene flow, which is unlikely given historical separation of these geographically distinct populations. In contrast, the migration rates estimated in our original inference were considerably lower, with nearly all total rates falling below 10% (Figure 5, Text S9, Table S12), indicating a better fit of that topology to the data.

Another set of alternative topologies we examined is one in which the dog clade originates from one of the four branches in the wolf sub-phylogeny (e.g. Figure 5C). Assuming such topologies, *G-PhoCS* infers that dogs diverged from wolves less than 200 years after wolves diverged from each other (Figure S5), whereas in the original inference conditioned on the NJ tree, the divergence between dogs and wolves was estimated to have occurred 1,400 years before the divergence between wolf populations. All other parameter estimates were not significantly affected by the choice of origin population for the dog clade. Thus regardless of our assumptions on the identity of the wolf population from which dogs originated, we infer that dogs diverged from the sampled wolf populations at about the same time these wolf populations diverged from each other. Additionally, the greater difference between estimated divergence times in our original analysis provides some support for our initial assumption that dogs and wolves form sister clades.

### Assessment of models in lights of site configuration statistics

Because *G-PhoCS* does not yet support statistical tests for model selection, we assessed relative support for the alternative models by performing simulations under each model, and comparing the simulated and real data with respect to a series of site configuration statistics informative about the topologies of local genealogies. For every quartet in our sample set that contains the jackal outgroup, we computed the relative frequencies of bi-allelic sites in which each of the two alleles (denoted A and B) is present in exactly two of the four individuals. Similar statistics are used in the ABBA-BABA test for admixture, but in our case we were also interested in the frequency of the BBAA configuration, which is the one compatible with the topology of the assumed phylogeny (see Text S8 for more information). We compared frequencies of the three configurations in 20 quartets observed in our data with those observed in data simulated under the three demographic models shown in Figure 5, denoted as “dog/wolf reciprocal monophyly” (Figure 5A), “regional domestication” (Figure 5B), and “ISW-source” (Figure 5C). This comparison allowed us to draw conclusions regarding the fit of each of these models to the data with respect to the distribution of local genealogies it implies (Table S10).

The three models appeared to be fairly compatible with the data overall, with the reciprocal monophyly model showing the lowest discrepancy (absolute error = 0.43), followed closely by the ISW-source model (absolute error = 0.47) and then trailed by the regional domestication model (absolute error = 0.82). The regional domestication model showed the largest discrepancy in quartets including Dingo and at least one other dog, indicating considerably weaker support for the dog clade and its internal structure than present in the data. This implies that the patterns of sequence similarity between dogs are more compatible with a distinct dog clade than they are with similarity solely generated by gene flow between the different dog lineages. The ISW-source model showed high discrepancy in quartets containing the Croatian and Israeli wolves, indicating that the model has problems capturing the phylogenetic relationships between those wolves and the dogs. The reciprocal monophyly model provided the best fit to the data, but it did show some discrepancy in quartets containing both the Dingo and the Chinese wolf. This is perhaps related to the large credible intervals for the rates of gene flow between these populations in the *G-PhoCS* inference (CHW→DNG, 0–6%; DNG→CHW, 2–6%). In conclusion, these tests show that topological signatures in the data provide strong support for a monophyletic dog clade and somewhat weaker support for a monophyletic wolf clade.

### Amylase expansion and dog origins

**Figure 6:**
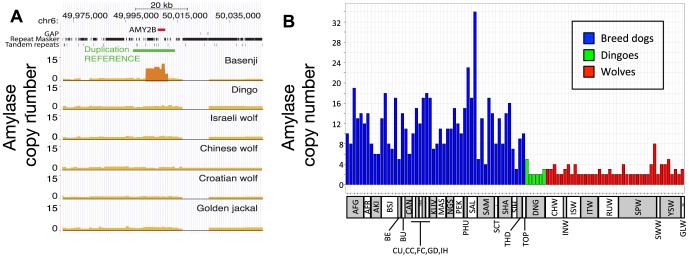
Copy number variation at amylase (*AMY2B*) locus. (A) Copy number variation (CNV) at *AMY2B* estimated from whole genome sequence data, showing presence of elevated copy number in Basenji but not in other lineages. Results are based on SOLiD data, except for the Chinese wolf (see Text S6 for supporting results and Text S10 for CNV analyses in an additional 12 dog breeds). (B) qPCR results on CNV state in an expanded set of wolf and dog lineages. Abbreviations for lineages are: AFG, Afgan Hound; AFR, Africanis; AKI, Akita; BSJ, Basenji; BE, Beagle; BU, Bulldog, CAN, Canaan Dog; CU, Chihuahua; CC, Chinese Crested; FC, Flat-coated Retriever; GD, Great Dane; IH, Ibizan Hound; KUV, Kuvasz; MAS, Mastiff; NGS, New Guinea Singing Dog; PEK, Pekinese; PHU, Phu Quoc; SAL, Saluki; SAM, Samoyed; SCT, Scottish Terrier; SHA, Shar Pei; SIH, Siberian Husky; THD, Thai Dog; TOP, Toy Poodle; DNG, Dingo; CHW, Chinese wolf; INW, Indian wolf; ISW, Israeli wolf; ITW, Italian wolf; RUW, Russian wolf; SPW, Spanish wolf; YSW, Yellowstone wolf; GLW, Great Lakes wolf.

Our inference of a pre-agriculture origin of dogs provides an important context for re-assessing the recent hypothesis that copy number expansion at the amylase locus (*AMY2B*) in dogs was an important part of the domestication process [15]. In that study, copy number segregated between species, with only two copies of the gene in each of the 35 wolves genotyped and an average 7.4-fold increase across 136 dogs. This finding was interpreted to suggest that *AMY2B* expansion enabled early dogs to exploit a starch-rich diet as they fed on refuse from agriculture. Surprisingly, and using the corrected depth of coverage to estimate discrete gene copy number, we find the Dingo has just two copies of *AMY2B* (Figure 6A, Text S6), suggesting that the *AMY2B* copy number expansion was not fixed across all dogs early in the domestication process. In a survey of sequence data from 12 additional domestic dog breeds, we find that the Siberian Husky, a breed historically associated with nomadic hunter gatherers of the Arctic, has only three to four copies of *AMY2B*, whereas the Saluki, which was historically bred in the Fertile Crescent where agriculture originated, has 29 copies (Figure S6). In order to validate the results, we used real-time quantitative PCR (qPCR) to explore the variation in *AMY2B* copies across additional breed dogs (n = 52), additional dingoes (n = 6) and a worldwide distribution of wolves (n = 40) (Text S6). The qPCR results show modern dog breeds on average have a high copy number of *AMY2B* and that wolves and Dingoes do not (Figure 6B, Table S13). However, the qPCR results also shows that the *AMY2B* expansion is polymorphic in wolves (16 of 40 wolves with >2 copies Figure 6B) and thus is not restricted to dogs.

## Discussion

In this study, we generated high-quality individual canid genomes, and used them to uncover the history of dogs and gray wolves. Interpretation of the phylogenetic signals in these genomes was particularly challenging due to high levels of incomplete lineage sorting and post-divergence gene flow. We were able to disentangle the effects of these factors by using an array of recently developed statistical methods that together provided a detailed and robust inference of past demography for these canids. We used methods that rely on different aspects of this dataset: 1) whole-genome patterns of heterozygosity in single individuals (PSMC), 2) a subset of sites that are informative for post-divergence admixture (ABBA/BABA analyses) and 3) a set of neutral loci analyzed jointly across all individuals (*G-PhoCS*).

We found evidence of wolf-dog admixture in two divergent dog lineages (Basenji and Dingo). The fact that these lineages have been geographically isolated from wolves in the recent past suggests that this gene flow was ancestral and thus likely impacted multiple (if not most) dog lineages [28], [29]. Admixture has likely complicated previous inferences of dog origins. For instance, the presence of long shared haplotypes in Middle East wolves with several dog breeds [10] may reflect historic admixture rather than recent divergence. Similarly, elevated genetic diversity in East Asian dogs and affinities between East Asian village dogs and wolves [7], [9], [24] may be confounded by past admixture with wolves. In areas where village dogs [30] roam freely and wolves have historically been in close proximity, admixture may also be present and exert a non-trivial impact on patterns of genetic variation [21].

Our inferences of ancestral population size from both PSMC and *G-PhoCS* revealed an unexpected, roughly threefold population bottleneck in wolves. With PSMC, we detect the start of this bottleneck as early as 20 kya, while with *G-PhoCS* the bottleneck occurs at the timing of dog-wolf divergence, approximately 15 kya. Because our simulations indicated that the timing of abrupt changes in *N_e_* are overestimated by PSMC (Text S8, S9, Figure S2), we place higher confidence in the more recent date inferred with *G-PhoCS*. Regardless of the method chosen, the bottleneck in wolves appears to have occurred well in advance of direct extermination campaigns by humans and within the timeframe of environmental and biotic changes associated with the ending of the Pleistocene era. Although the specific cause of this bottleneck is unknown, it has important implications for dog domestication. Because of this bottleneck, we expect that at the onset of domestication, there was substantially more genetic diversity for selection to act on than what is observed in modern wolves. Direct comparisons of dog and wolf diversity (such as comparisons of heterozygosity) will not show as large a difference and thus previous studies that did not consider a wolf population decline [22], [23] have underestimated the bottleneck associated with domestication. These previous studies estimated a two to fourfold reduction in dog *N_e_*, a far milder population contraction than the at least 16-fold reduction we infer here.

We provide several lines of evidence supporting a single origin for dogs, and disfavoring alternative models in which dog lineages arise separately from geographically distinct wolf populations (Figures 4–5, Table S10). Considering a full multi-population demographic model with gene flow, we infer that dogs diverged from wolves at around 15 kya (CI: 14–16 kya). Examination of previous estimates shows a wide range of suggested divergence times [24], [25]. However, most of the discrepancy between different studies can be traced to differences in the assumed mutation rate. We assume an average mutation rate per generation of 1×10^−8^ and an average generation time of three years. However, we observed that CpG di-nucleotides, which we filtered out from the data, contribute roughly 30% of mutations in these canid genomes, similar to what was observed in human genomes [19]. Thus our assumptions regarding mutation rate imply a genome-wide rate (i.e. including filtered sites) of 1.4×10^−8^. Other studies of dog domestication assume slightly lower genome-wide rates. For instance, a recent study based on shotgun sequencing data [25] assumes a mutation rate of 1×10^−8^ and estimates the divergence time to be 14 kya (CI:11–18 kya) or 30 kya (CI:15–90 kya), depending on the assumed amount of gene flow. Another recent study [20] assumes an even lower mutation rate of 0.66×10^−8^ and estimates the divergence time at roughly 32 kya. Calibrating the different estimates using the same mutation rate shows a remarkable consistency with our conclusions. Unfortunately, very little is known about dog mutation rates, and estimates of mammalian mutation rates range from 0.22×10^−8^ per year (i.e., 0.66×10^−8^ per generation) [31] to 1.8×10^−8^ per generation [32]. Considering this wide range expands the credible interval for the divergence time of dogs and wolves from 14–16 kya to 11–34 kya. Importantly, our study was able to eliminate much of the uncertainty in the mutation-scaled divergence time (CI: 0.46×10^−4^–0.53×10^−4^), leaving the mutation rate as the dominant source of uncertainty in dating the origin of dogs.

The divergence time between dogs and wolves provides an estimated upper bound for the time of domestication. We can also estimate a lower bound as the divergence time between the Dingo and the population ancestral to Basenji and Boxer, which we infer at 13 kya (CI: 11–12 kya, 9–25 kya assuming a range of mutation rates). Thus, our demographic analysis strongly suggests that domestication occurred between about 11 and 16 kya (9–34 kya with mutation rate uncertainty), which would place it prior to the adoption of extensive agriculture by humans. This finding is consistent with the fossil record, but it raises questions regarding the hypothesis that the advent of agriculture created a novel niche that was the driving force in dog domestication [15]. Our examination of *AMY2B* confirmed previously reported high copy numbers across almost all dog breeds [15]. However, we also found variation in copy numbers across wolf populations, and low copy numbers in dog lineages that are not associated with agrarian societies (Dingo and Husky). This suggests a more complicated history of the high copy number variants of *AMY2B*, which likely existed already as standing variation in early domestic dogs, but expanded more recently with the development of large agriculturally based civilizations in the Middle East, Europe and Eastern Asia.

Overall, the genomes sequenced in this study reveal a dynamic and complex genetic history interrelating dogs and wolves. One question that remains unanswered is that of the geographic origin of dogs and the wolf lineage most closely related to them. Our analysis suggests that none of the sampled wolf populations is more closely related to dogs than any of the others, and that dogs diverged from wolves at about the same time that the sampled wolf populations diverged from each other (Figures 5A, 5C). One possible implication of this finding is that a more closely related wolf population exists today, but was not represented by our samples. We consider this unlikely, as we sampled the three major putative domestication regions, and previous SNP array studies demonstrate that wolf populations are only weakly differentiated, indicating that the wolves we sampled should serve as good proxies for wolves in each broad geographic region [10].

Another alternative is that the wolf population (or populations) from which dogs originated has gone extinct and the current wolf diversity from each region represents novel younger wolf lineages, as suggested by their recent divergence from each other (Figure 5A). Our inference that wolves have gone through bottlenecks across Eurasia (Figures 3B, 5A) suggests a dynamic period for wolf populations over the last 20,000 years and that extinction of particular lineages is not inconceivable. Indeed, several external lines of evidence provide support for substantial turnover in wolf lineages. For example, ancient DNA, isotope, and morphologic evidence identify a divergent North American Late Pleistocene wolf [33] and in Eurasia, similarly distinct wolves exist in the early archaeological record in Northern Europe and Russia, 15–36kya [3]–[5]. Presumed changes in available prey (e.g. megafaunal extinctions) as habitats shrunk with the expansion of humans and agriculture also suggest the plausibility of wolf population declines and lineage turnover. A remaining alternative to our inferred population phylogeny is that the basal lineage was absorbed into the three lineages sampled. Such a hypothesis is questionable, though, as it requires there to be enough effective gene flow among the three wolf lineages such that no single lineage today serves best as a proxy for the basal lineage in our analysis. If true, the hypothesis that dogs were originally domesticated from a now-extinct wolf population suggests that ancient DNA studies will play a central role in advancing our understanding of the rapid transition from a large, aggressive carnivore to the omnivorous domestic companion that is a fixture of modern civilization.

## Materials and Methods

### Samples and sequencing

We selected six samples for genome sequencing and generated single end and long mate pair SOLiD reads. We generated additional paired end (PE) sequence data on the Illumina HiSeq platform (Text S1). For most downstream analyses, we also utilized sequence information from the Boxer reference genome (CanFam 3.0).

### Sequence alignment, genotyping, and filters

We aligned sequence reads to CanFam 3.0, with post-processing of aligned reads including the removal of duplicates, local realignment, and base quality recalibration (Text S3). We then genotyped each sample individually, using the Genome Analysis Toolkit (GATK) pipeline [34]. For SNV genotyping and analysis, we excluded repeats of recent origin, CpG sites, regions falling in copy number variants, and triallelic sites, and at the sample level we filtered out genotypes proximate to called indels, with excess depth of coverage, with low genotype quality scores, or where the SNV fell within five base pairs of another SNV (Text S4).

### Genotype validation

We compared genotype calls based upon sequencing to those from the same samples using the Illumina CanineHD BeadChip, which consists of >170,00 markers evenly spaced throughout the dog genome (Text S5). We also analyzed variants overlapping those generated in a previous SNP array study of a large panel of dogs and wolves [10], and performed PCA on the combined data set to verify that NGS genotypes clustered with array genotypes for the same lineages (Text S5).

### Structural variant detection

We delineated segmental duplications in our six genomes by identifying regions with a significant excess depth of coverage (Text S6). For this purpose, we aligned Illumina and SOLiD reads with MrFAST [35] and drFAST [36] respectively. Absolute copy numbers were calculated using mrCaNaVar version 0.31 (http://mrcanavar.sourceforge.net/). In the particular case of the previously reported *AMY2B* expansion in the dog lineage [15] we also examined patterns of copy number across 52 breed dogs, six Dingoes, and 40 wolves using qPCR (Text S6).

### Functional element annotation

In order to conduct demographic analyses on putatively neutral genomic regions without any apparent functional annotation, we first identified genic region using annotations from the union of refGene, Ensembl and SeqGene annotation databases, with the condition that all annotated transcripts had proper start and stop codons, and contained no internal stop codons (Text S7). In addition, we defined conserved non-coding elements (CNEs) on the basis of phastCons scores [37] (Text S7).

### *N_e_* through time: Pairwise-Sequential-Markov-Coalescent (PSMC)-based inference

We used the PSMC methods developed by Li and Durbin [20] to infer the trajectory of population sizes across time for the six canid genome sequences (Text S8).

### Testing for admixture: ABBA-BABA

To investigate the extent of gene flow between wolves and dogs subsequent to their divergence, we employed a method recently developed by Durand et al. [18]. This method tests for directional gene flow by testing for asymmetries in allele sharing between a source lineage (P3), and either of two receiving lineages (P1, P2) with reference to an outgroup (O). To focus on gene flow most germane to evolutionary processes influencing wolf-dog divergence, we restricted testing to those cases where one of the dog samples was P3, the other two (P1 and P2) were wolves, and viceversa (P3 = wolf, P1 and P2 = dogs). For more details, see Text S8.

### Demographic model for dog domestication

Our main demographic analysis is based on the Generalized Phylogenetic Coalescent Sampler (*G-PhoCS*) developed by Gronau et al. [19] and which we applied to 16,434 1 kb loci chosen via a strict set of criteria to obtain putatively neutral loci (Text S9). The prior distributions over model parameters was defined by a product of Gamma distributions using the default setting chosen by Gronau et al. [19]. Markov Chain was run for 100,000 burn-in iterations, after which parameter values were sampled for 200,000 iterations every 10 iterations, resulting in a total of 20,001 samples from the approximate posterior. Convergence was inspected manually for each run. We conditioned inference on the population phylogeny based upon the neighbor-joining tree constructed from the genome-wide distance matrix described above (Fig. 4A). We also constructed models under a ‘regional domestication’ scenario, in which each dog lineage originated from a wolf lineage from the same geographic region, i.e. Basenji from Israeli wolf, Boxer from Croatian wolf, and Dingo from Chinese wolf. We assessed models in which the branch ancestral to dogs was sister to a particular extant wolf population, or one of internal branches in the wolf clade. In addition, we investigated the sensitivity of parameter estimates to choice of locus length, number of loci, intra-locus recombination, distance from coding exons, and selection on linked sites. For more details, see Text S9.

## Supporting Information

Figure S1 Neighbor-joining tree of canid samples plus the Boxer reference (CanFam3.0) for all positions passing the GF2 and SF filters and for which there was no missing data for any sample. The distance metrics used were equations E8.1 and E8.2 (see Text S8) for panel A) and B), respectively. For each branch, we report the genetic distance (left side of the slash) and the bootstrap support (right side of the slash). Bootstrap replicates were generated by dividing the genome of each species into windows of 500 kb based on the genomic coordinates of the Boxer reference, and then resampling with replacement from those windows until the bootstrapped genomes for each species contain an equal or greater number of sites called as the true genomes.
(PDF)

Figure S2 *N_e_* trajectories of 6 canid lineages reconstructed using the PSMC method of Li and Durbin [20], for data simulated under the *G-PhoCS* inferred demographic history, including all detected gene flow. The actual *N_e_* trajectories are shown as dotted lines whereas the inferred *N_e_* trajectories are depicted by solid lines.
(PDF)

Figure S3 Estimates obtained by *G-PhoCS* for data simulated under a demographic model implied by the ancestral effective population sizes inferred by PSMC. Twelve data sets were simulated according to the ancestral effective population sizes estimated by PSMC, and using three levels of recombination (see text). Four replicates were generated for each recombination rate. These data were analyzed with *G-PhoCS* using the same population phylogeny and migration bands assumed in our main analysis (without the BOX population). Estimates of demographic parameters in the twelve simulated data sets are shown with 95% Bayesian credible intervals. Raw estimates, scaled by mutation rate (×10^4^), are shown (left axis) next to calibrated estimate (right axis) (see Text S9 for details on calibration). Horizontal bars indicate true values assumed for divergence times in the simulation (red) and values estimated from real data by *G-PhoCS* (dashed blue).
(PDF)

Figure S4 Regional origins for dogs. (A) A population phylogeny for dogs and wolves describing a demographic scenario in which dogs have been domesticated separately in each geographic region. There are three possible topologies describing such scenarios; each of the three is determined by the topology over the three ancestral populations, EUR, EAS, and MEA (dashed). We considered post-divergence gene flow between wolf populations (red) and between dog populations (blue), as well as gene flow with golden jackal (gray). (B) Estimates and 95% Bayesian credible intervals for select demographic parameters under the three topologies consistent with regional origins. Each bar plot describes estimates for a given parameter obtained by *G-PhoCS* in six different runs: three runs without any migration band (left three bars), and three runs with the 16 migration bands shown in panel A (right three bars). Raw estimates, scaled by mutation rate (×10^4^), are shown (left axis) next to calibrated estimate (right axis) (see Text S9. for details on calibration). Estimates of τ_anc_DOG and τ_anc_DW obtained in our main analysis are shown for comparison (horizontal blue bars).
(PDF)

Figure S5 Alternative hypotheses for origin for dog clade. (A) Five possible branches in the wolf sub-phylogeny were considered as a sister branch to the root of the dog clade (ancDOG): ancWLF, ISW, CRW, CHW, and ancWLF1. The tree inferred by neighbor joining suggests that the sister branch of the dog clade is the one at the root of the wolf clade (ancWLF). We ran *G-PhoCS* assuming each of the other four alternative topologies with the eight migration bands assumed in our main analysis (gray). (B) Estimates and 95% Bayesian credible intervals for select demographic parameters under the five possible topologies. Estimates obtained using the default topology are highlighted (red). Raw estimates, scaled by mutation rate (×10^4^), are shown (left axis) next to calibrated estimate (right axis) (see Text S9 for details on calibration). The estimated difference between divergence times, Δ_τ_ = τ_anc_ DW−τ_anc_ WLF, is also shown.
(PDF)

Figure S6 Copy number in 12 breed dogs around AMY2B exons on chr6. Copy number was calculated for each base and plotted on the y-axis. Red lines indicate the syntenic positions of the human AMY2B transcript ENST00000361355. The blue line indicates the region across which average copy number was measured. Average copy number is indicated by the horizontal line and printed value. The dotted green line indicates the approximate boundaries of the copied sequence.
(PDF)

Table S1 Sequence data generated, rate of PCR duplicates, alignment statistics, and mean depth of coverage per sample.
(PDF)

Table S2 Percentage of genome genotyped and containing variants.
(PDF)

Table S3 Counts of variant site configurations at sites with no missing data.
(PDF)

Table S4 Concordance between high-quality Illumina array genotypes and genotypes obtained from genotyping pipeline. Proportions are normalized by row, reflecting concordance conditional on the chip genotype, based upon sites that passed GF3 and SF.
(PDF)

Table S5 Estimated heterozygote discordance rates and reference bias in genotypes called from sequencing relative to genotypes called on the Illumina CanineHD BeadChip.
(PDF)

Table S6 Autosomal heterozygosity for six canid genomes.
(PDF)

Table S7 Genome-wide pairwise sequence divergence, estimated using equation E8.1 (see Text S8) using all the genomic sites that passed the genomic quality filters outlined in Text S8.
(PDF)

Table S8 Genome-wide pairwise sequence divergence, estimated using equation E8.2 (see Text S8) using all the genomic sites that passed the genomic quality filters outlined in Text S8.
(PDF)

Table S9 Estimates of the number of ABBA/BABA/BBAA sites in the six canid genomes. For each cell and each quartet comparison we report the number of ABBA/BABA/BBAA sites followed by the frequency of those three types of sites given that the site is bi-allelic with the two alleles found in two species each. The golden jackal was used as an outgroup in all comparisons.
(PDF)

Table S10 Estimates of the number of ABBA/BABA/BBAA sites in the three *G-PhoCS* models analyzed. For each cell and each quartet comparison we report: 1) The number of ABBA/BABA/BBAA sites; 2) The frequency of those three types of sites given that the site is bi-allelic with the two alleles found in two species each and 3) the difference of that frequency in the simulations minus what is estimated in the data (when this difference is bigger than 1.5%, we highlight the cell in bold). The lower row of the table indicates the fit of the model to the data as estimated by equation 8.7 in Text S8. The golden jackal was used as an outgroup in all comparisons.
(PDF)

Table S11 Estimation of post-divergence gene flow using the D Statistic [18]. The outgroup in all comparisons is the golden jackal. Statistical significance is evaluated using a two-tailed Z test, with the additional requirement that that absolute value of the Z-score to be ≥3. Significant tests and sample pairs showing evidence for post-divergence gene flow are shown in bold.
(PDF)

Table S12 Main set of parameter estimates in the *G-PhoCS* analysis.
(PDF)

Table S13 qPCR results for copy number at *AMY2B* for 52 breed dogs, 6 Dingoes, and 40 wolves representing their global distribution.
(PDF)

Text S1 Information on samples chosen for genome sequencing.
(PDF)

Text S2 Details concerning multi-platform library construction and sequencing strategy, read alignment statistics, and sequencing depth of coverage.
(PDF)

Text S3 Information on sequence alignment and genotyping pipeline methods.
(PDF)

Text S4 Information concerning quality filters applied to genotype data.
(PDF)

Text S5 Methods and results related to the validation of genotype calls made from whole genome sequencing data for our six canid samples.
(PDF)

Text S6 Methods and supporting results for structural variant calling.
(PDF)

Text S7 Methods for annotation of genes and conserved non-coding regions.
(PDF)

Text S8 Methods and supporting results for demographic analyses using sequence divergence, ABBA/BABA tests and PSMC.
(PDF)

Text S9 Methods and supporting results for demographic analyses using *G-PhoCS*.
(PDF)

Text S10 Copy number status of the amylase gene (*AMY2B*) on CFA6 in 12 dog breeds.
(PDF)

Text S11 Comparison of golden jackal sample to jackals and wolves.
(PDF)
