# Correction: Genome Sequencing Highlights the Dynamic Early History of Dogs

**DOI:** 10.1371/journal.pgen.1004631

**Published:** 2014-08-12

**Authors:** The PLOS Genetics Staff

Alan Wilton is not included in the author byline. He should be listed as the 23rd author and affiliated with School of Biotechnology & Biomolecular Sciences, Faculty of Science, The University of New South Wales, Sydney, NSW 2052, Australia. The contributions of this author are as follows: sample collection.

The following sentence should also be included in the acknowledgements. "The authors also wish to thank the Australian Native Dog Conservation Society and the Dingo Sanctuary Bargo for generously providing the dingo sample used in this study."
