# Supplementary figures and images for: Genome Sequencing Highlights the Dynamic Early History of Dogs
Source: PLoS Genet. 2014 Jan 16;10(1):e1004016. doi: 10.1371/journal.pgen.1004016 (PMC3894170; doi:10.1371/journal.pgen.1004016)

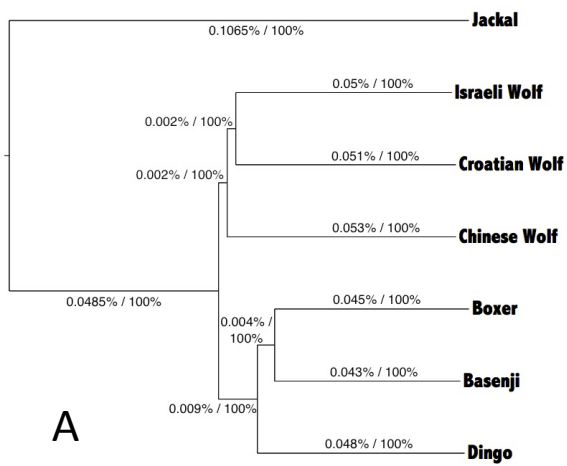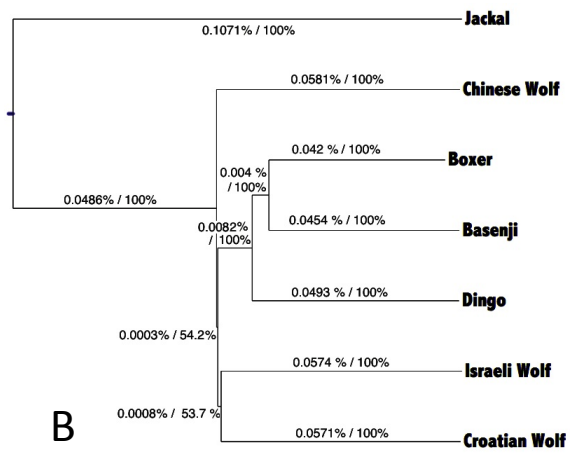

Supplement: Figure S1 — Neighbor-joining tree of canid samples plus the Boxer reference (CanFam3.0) for all positions passing the GF2 and SF filters and for which there was no missing data for any sample. The distance metrics used were equations E8.1 and E8.2 (see Text S8) for panel A) and B), respectively. For each branch, we report the genetic distance (left side of the slash) and the bootstrap support (right side of the slash). Bootstrap replicates were generated by dividing the genome of each species into windows of 500 kb based on the genomic coordinates of the Boxer reference, and then resampling with replacement from those windows until the bootstrapped genomes for each species contain an equal or greater number of sites called as the true genomes. (PDF) [file pgen.1004016.s001.pdf]

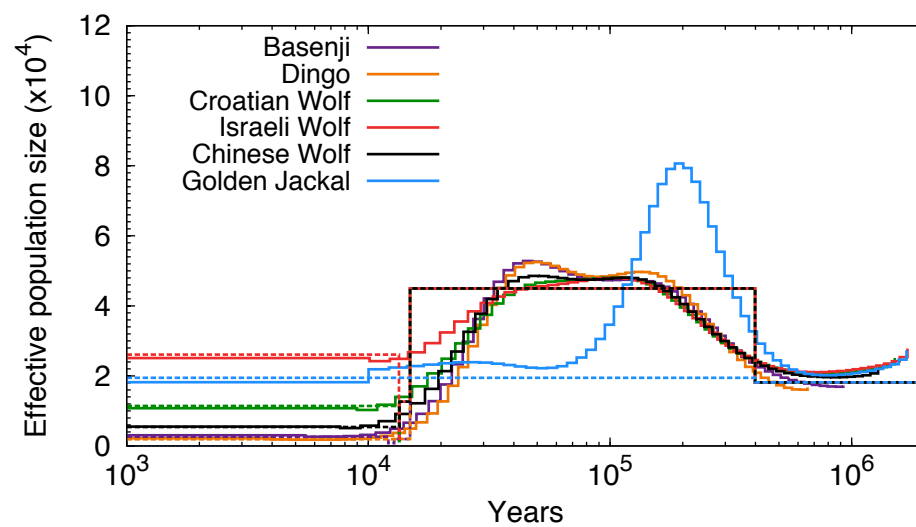

Supplement: Figure S2 — Ne trajectories of 6 canid lineages reconstructed using the PSMC method of Li and Durbin [20], for data simulated under the G-PhoCS inferred demographic history, including all detected gene flow. The actual Ne trajectories are shown as dotted lines whereas the inferred Ne trajectories are depicted by solid lines. (PDF) [file pgen.1004016.s002.pdf]

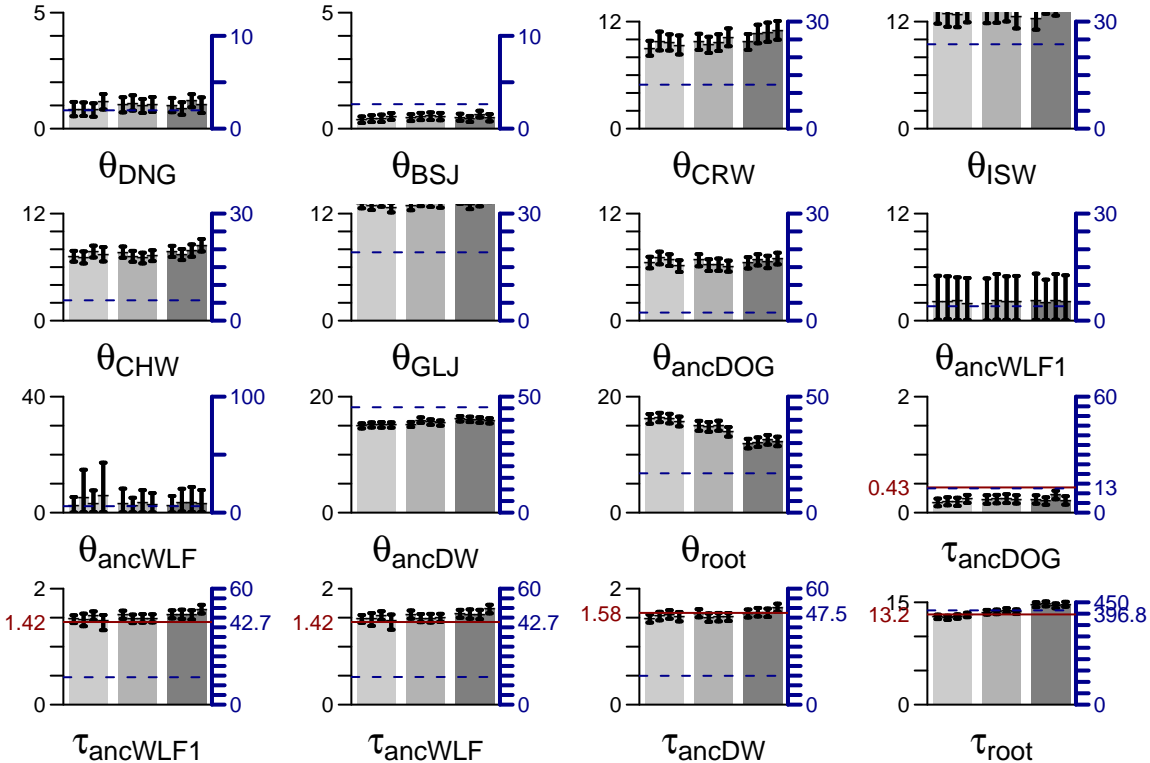

Supplement: Figure S3 — Estimates obtained by G-PhoCS for data simulated under a demographic model implied by the ancestral effective population sizes inferred by PSMC. Twelve data sets were simulated according to the ancestral effective population sizes estimated by PSMC, and using three levels of recombination (see text). Four replicates were generated for each recombination rate. These data were analyzed with G-PhoCS using the same population phylogeny and migration bands assumed in our main analysis (without the BOX population). Estimates of demographic parameters in the twelve simulated data sets are shown with 95% Bayesian credible intervals. Raw estimates, scaled by mutation rate (×104), are shown (left axis) next to calibrated estimate (right axis) (see Text S9 for details on calibration). Horizontal bars indicate true values assumed for divergence times in the simulation (red) and values estimated from real data by G-PhoCS (dashed blue). (PDF) [file pgen.1004016.s003.pdf]

**A**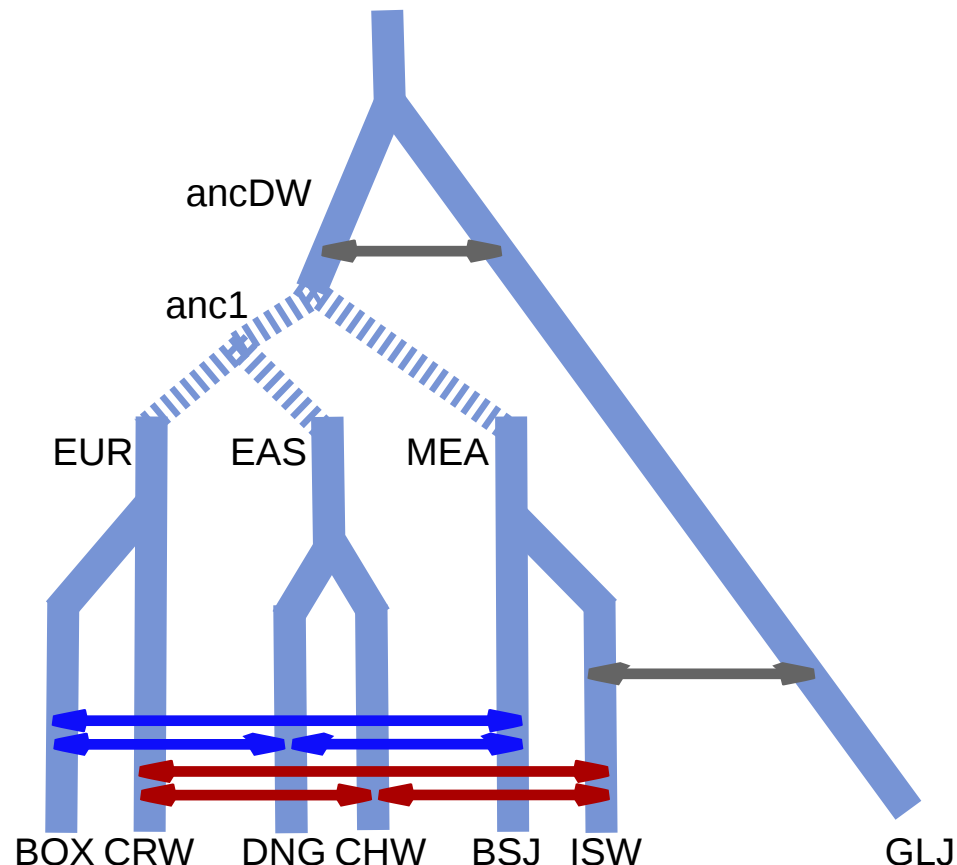**B**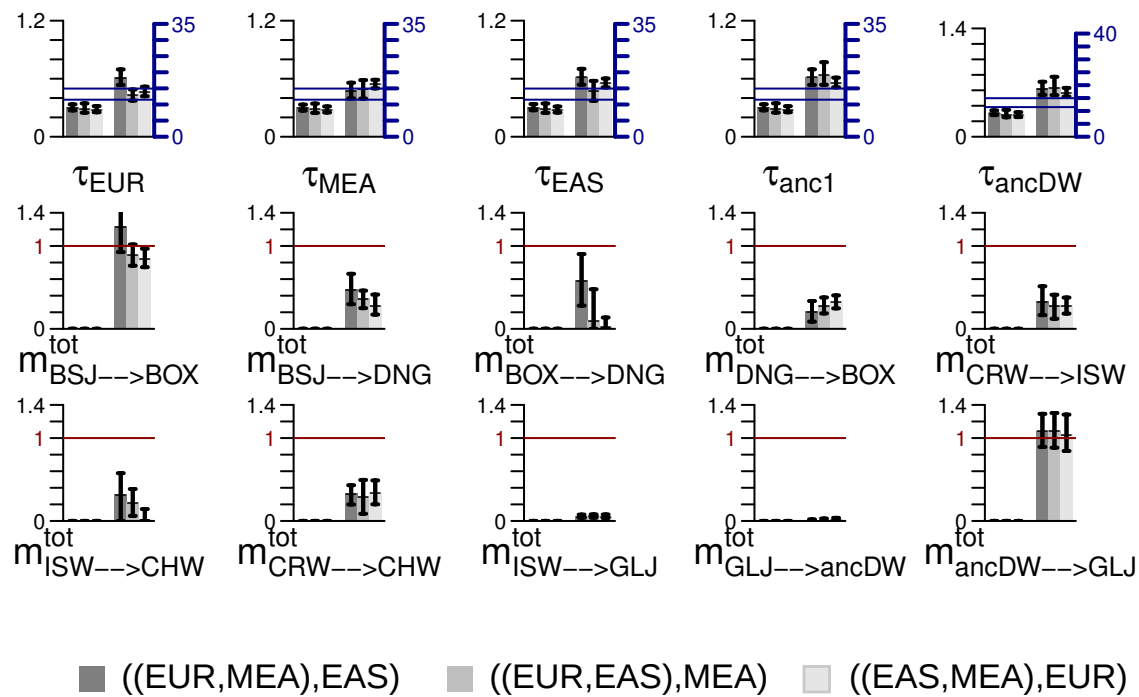

Supplement: Figure S4 — Regional origins for dogs. (A) A population phylogeny for dogs and wolves describing a demographic scenario in which dogs have been domesticated separately in each geographic region. There are three possible topologies describing such scenarios; each of the three is determined by the topology over the three ancestral populations, EUR, EAS, and MEA (dashed). We considered post-divergence gene flow between wolf populations (red) and between dog populations (blue), as well as gene flow with golden jackal (gray). (B) Estimates and 95% Bayesian credible intervals for select demographic parameters under the three topologies consistent with regional origins. Each bar plot describes estimates for a given parameter obtained by G-PhoCS in six different runs: three runs without any migration band (left three bars), and three runs with the 16 migration bands shown in panel A (right three bars). Raw estimates, scaled by mutation rate (×104), are shown (left axis) next to calibrated estimate (right axis) (see Text S9. for details on calibration). Estimates of τancDOG and τancDW obtained in our main analysis are shown for comparison (horizontal blue bars). (PDF) [file pgen.1004016.s004.pdf]

**A**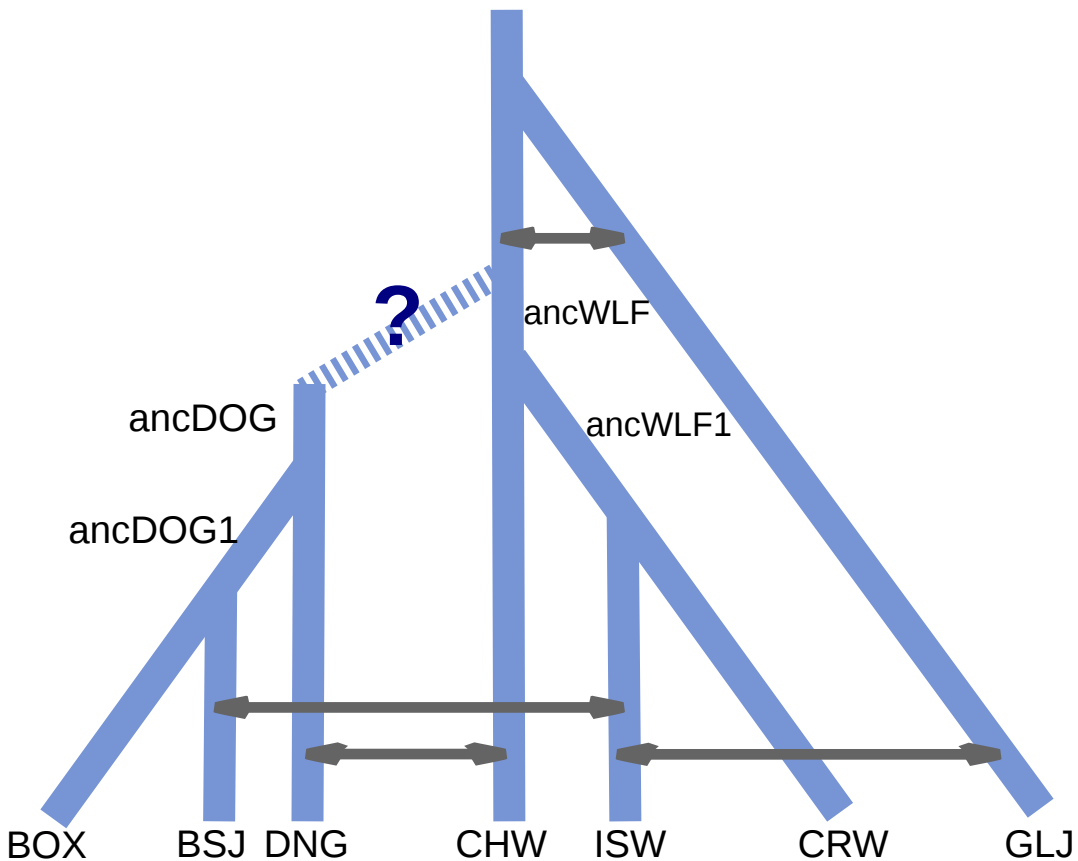**B**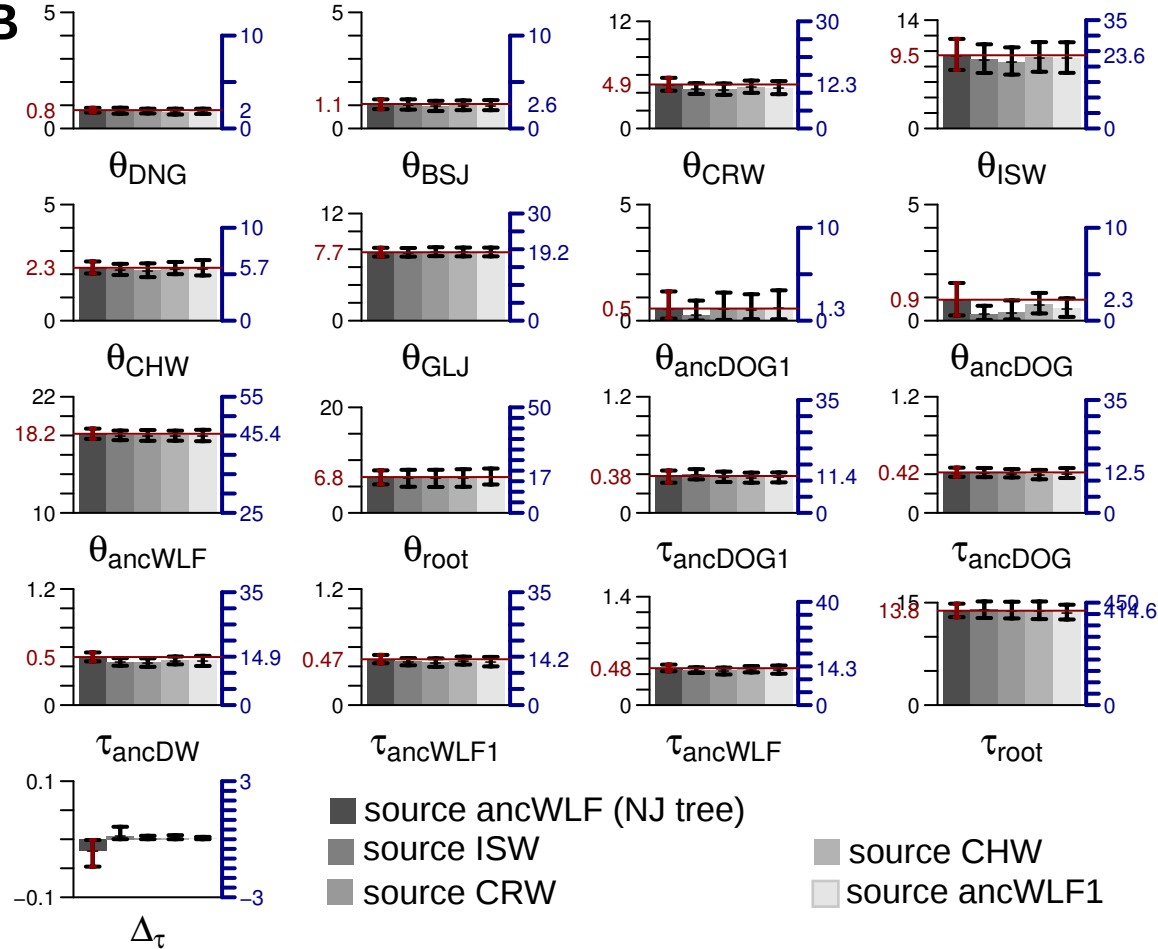

Supplement: Figure S5 — Alternative hypotheses for origin for dog clade. (A) Five possible branches in the wolf sub-phylogeny were considered as a sister branch to the root of the dog clade (ancDOG): ancWLF, ISW, CRW, CHW, and ancWLF1. The tree inferred by neighbor joining suggests that the sister branch of the dog clade is the one at the root of the wolf clade (ancWLF). We ran G-PhoCS assuming each of the other four alternative topologies with the eight migration bands assumed in our main analysis (gray). (B) Estimates and 95% Bayesian credible intervals for select demographic parameters under the five possible topologies. Estimates obtained using the default topology are highlighted (red). Raw estimates, scaled by mutation rate (×104), are shown (left axis) next to calibrated estimate (right axis) (see Text S9 for details on calibration). The estimated difference between divergence times, Δτ = τanc DW−τanc WLF, is also shown. (PDF) [file pgen.1004016.s005.pdf]

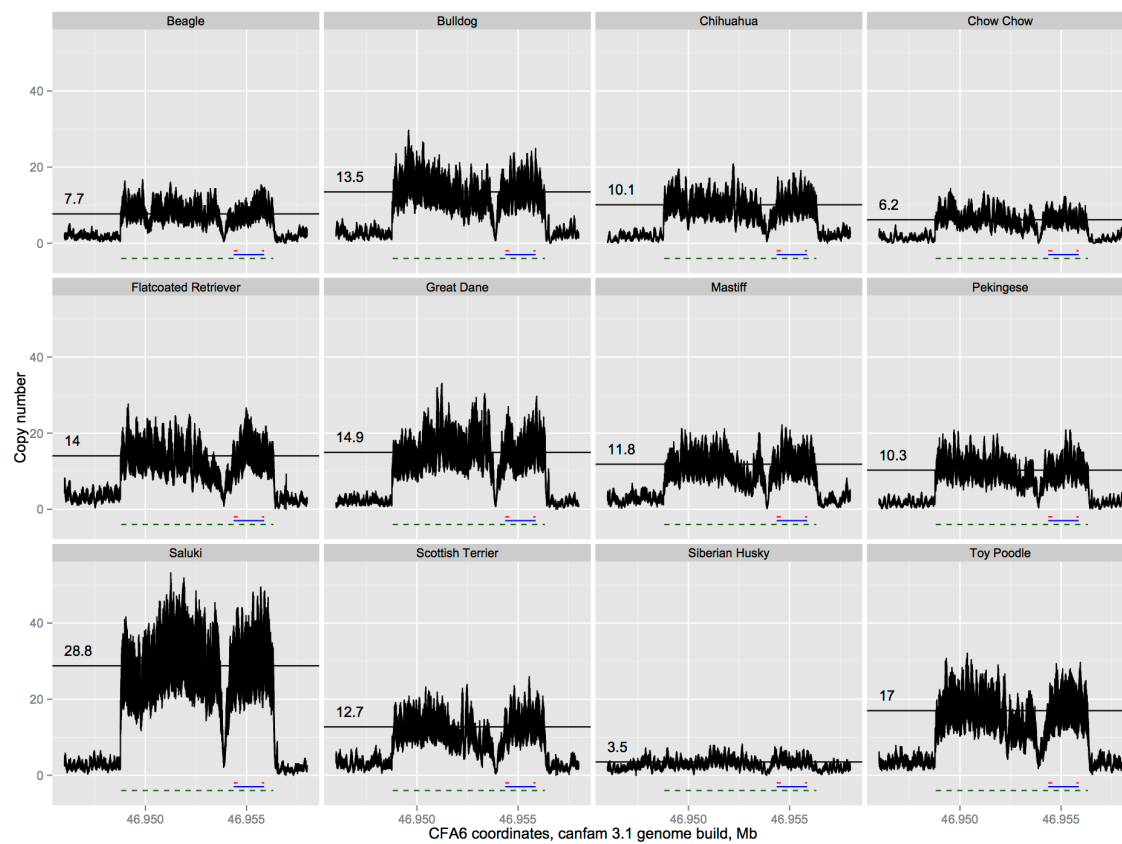

Supplement: Figure S6 — Copy number in 12 breed dogs around AMY2B exons on chr6. Copy number was calculated for each base and plotted on the y-axis. Red lines indicate the syntenic positions of the human AMY2B transcript ENST00000361355. The blue line indicates the region across which average copy number was measured. Average copy number is indicated by the horizontal line and printed value. The dotted green line indicates the approximate boundaries of the copied sequence. (PDF) [file pgen.1004016.s006.pdf]
