# Supplementary material for: Genome Sequencing Highlights the Dynamic Early History of Dogs
Source: PLoS Genet. 2014 Jan 16;10(1):e1004016. doi: 10.1371/journal.pgen.1004016 (PMC3894170; doi:10.1371/journal.pgen.1004016)
Supplement: Table S1 — Sequence data generated, rate of PCR duplicates, alignment statistics, and mean depth of coverage per sample. (PDF) [file pgen.1004016.s007.pdf]

Table S1. Sequence data generated, rate of PCR duplicates, alignment statistics, and mean depth of coverage per sample.

	**Basenji**	**Dingo**	**Israeli wolf**	**Chinese wolf**	**Croatian wolf**	**Golden jackal**
Total reads[a]	969.0	2194.5	1803.5	694.0	2180.8	3365.8
Reads aligned[a]	816.6	1602.2	1249.5	652.7	1464.5	2437.8
% Reads aligned	84.3	73.0	69.3	94.0	67.2	72.4
% PCR duplicates	28.5	28.5	20.2	3.2	16.0	51.5
Uniquely aligned reads[a]	581.3	1145.5	997.3	632.0	1229.8	1181.8
Uniquely aligned bases (Gb)	28.1	70.7	52.7	61.3	62.4	59.5
Mean coverage	12.6x	25.75x	21.6x	24.6x	25.3x	23.8x

[a] Millions of reads
