# Supplementary material for: Genome Sequencing Highlights the Dynamic Early History of Dogs
Source: PLoS Genet. 2014 Jan 16;10(1):e1004016. doi: 10.1371/journal.pgen.1004016 (PMC3894170; doi:10.1371/journal.pgen.1004016)
Supplement: Table S2 — Percentage of genome genotyped and containing variants. (PDF) [file pgen.1004016.s008.pdf]

Table S2. Percentage of genome genotyped and containing variants.

	Unfiltered		GF1 [b]		GF2 [c]		GF3 [d]		SF [e]		GF1+SF		GF2+SF		GF3+SF	
**Sample**	**All** [a]	**SNV** [f]	**All** [a]	**SNV** [f]	**All** [a]	**SNV** [f]	**All** [a]	**SNV** [f]	**All** [a]	**SNV** [f]	**All** [a]	**SNV** [f]	**All** [a]	**SNV** [f]	**All** [a,g]	**SNV** [f]
Basenji	96.81	0.21	54.89	0.14	67.13	0.14	68.61	0.19	74.63	0.18	43.95	0.13	54.43	0.13	55.31	0.17
Dingo	97.44	0.22	55.29	0.14	67.58	0.15	69.09	0.19	87.81	0.18	51.31	0.12	63.23	0.13	64.37	0.17
Israeli wolf	97.45	0.30	55.33	0.20	67.60	0.20	69.13	0.27	91.10	0.26	52.62	0.18	64.66	0.18	65.89	0.24
Croatian wolf	95.98	0.28	54.56	0.20	66.79	0.20	68.22	0.26	86.33	0.25	50.33	0.18	62.16	0.18	63.21	0.24
Chinese wolf	98.37	0.30	55.72	0.20	68.05	0.20	69.61	0.27	95.59	0.25	54.59	0.17	66.72	0.17	68.22	0.24
Golden jackal	96.88	0.46	55.18	0.32	67.45	0.32	68.95	0.43	88.11	0.38	51.42	0.27	63.37	0.28	64.50	0.37

[a] Proportion of the genome measured with respect to all 2194412237 non-'N' bases in the reference.

[b] Genome level filter, hard masking all repeats identified with RepeatMasker, CNVs, and CpG sites.

[c] Genome level filter, excluding CNVs, young repeat elements, and CpG sites.

[d] Genome level filter allowing CpG sites, used for analyses of genic regions.

[e] Sample level filter: minimum genotype quality=20, max. depth of coverage≤ 2x the mean

[f] Percentage of sites passing the filter that contain a non-reference allele. Additional filters exclude SNVs within 5bp of another, and SNVs within 5bp of indels.

[g] Proportions of GF3 masked sites genotyped are 78.76% (Basenji), 91.67% (Dingo), 93.84% (Israeli wolf), 90.03% (Croatian wolf), 97.16% (Chinese wolf), and 91.85% (Golden jackal).
