# Supplementary material for: Genome Sequencing Highlights the Dynamic Early History of Dogs
Source: PLoS Genet. 2014 Jan 16;10(1):e1004016. doi: 10.1371/journal.pgen.1004016 (PMC3894170; doi:10.1371/journal.pgen.1004016)
Supplement: Table S3 — Counts of variant site configurations at sites with no missing data. (PDF) [file pgen.1004016.s009.pdf]

Table S3. Counts of variant site configurations at sites with no missing data.

Site Configuration	Count
Shared between dogs and wolves	1524761
Fixed between dogs and wild canids	16604
Fixed between dogs and wolves[a]	7525
In dogs but not wolves	428339
In wolves but not dogs	867656
Basenji-specific	274063
Dingo-specific	261352
Israeli wolf-specific	473669
Croatian wolf-specific	460000
Chinese wolf-specific	451104
Golden jackal-specific	2205599
Total	6970672

[a] Jackal either fixed or polymorphic for the dog allele.
