# Supplementary material for: Genome Sequencing Highlights the Dynamic Early History of Dogs
Source: PLoS Genet. 2014 Jan 16;10(1):e1004016. doi: 10.1371/journal.pgen.1004016 (PMC3894170; doi:10.1371/journal.pgen.1004016)
Supplement: Table S4 — Concordance between high-quality Illumina array genotypes and genotypes obtained from genotyping pipeline. Proportions are normalized by row, reflecting concordance conditional on the chip genotype, based upon sites that passed GF3 and SF. (PDF) [file pgen.1004016.s010.pdf]

Table S4. Concordance between high-quality Illumina array genotypes and genotypes obtained from genotyping pipeline. Proportions are normalized by row, reflecting concordance conditional on the chip genotype, based upon sites that passed GF3 and SF.

Rows: Array Genotypes; Columns: Sequencing Genotypes

Basenji	0/0	0/1	1/1	Total
0/0	0.9997	0.0003	0.0000	38412
0/1	0.0020	0.9966	0.0013	16734
1/1	0.0001	0.0022	0.9976	30330

Dingo	0/0	0/1	1/1	Total
0/0	0.9998	0.0002	0.00002	47010
0/1	0.0008	0.9990	0.0002	11766
1/1	0.0003	0.0007	0.9990	38932

Israeli wolf	0/0	0/1	1/1	Total
0/0	0.9993	0.0006	0.0001	39245
0/1	0.0010	0.9985	0.0002	26115
1/1	0.0003	0.0029	0.9967	32263

Croatian wolf	0/0	0/1	1/1	Total
0/0	0.9994	0.0005	0.0001	40510
0/1	0.0017	0.9982	0.0001	21156
1/1	0.0003	0.004	0.9955	33582

Chinese Wolf	0/0	0/1	1/1	Total
0/0	0.9997	0.00024	0.00002	42234
0/1	0.0012	0.9987	0.00005	18466
1/1	0.0004	0.0005	0.9992	37006

Golden jackal	0/0	0/1	1/1	Total
0/0	0.9995	0.0003	0.0002	47779
0/1	0.0032	0.9942	0.0026	6906
1/1	0.0005	0.0042	0.9953	40606
