# Supplementary material for: Genome Sequencing Highlights the Dynamic Early History of Dogs
Source: PLoS Genet. 2014 Jan 16;10(1):e1004016. doi: 10.1371/journal.pgen.1004016 (PMC3894170; doi:10.1371/journal.pgen.1004016)
Supplement: Table S5 — Estimated heterozygote discordance rates and reference bias in genotypes called from sequencing relative to genotypes called on the Illumina CanineHD BeadChip. (PDF) [file pgen.1004016.s011.pdf]

Table S5. Estimated heterozygote discordance rates and reference bias in genotypes called from sequencing relative to genotypes called on the Illumina CanineHD BeadChip.

Sample	Heterozygote discordance per bp	Reference bias		
		Overall[a]	0/1 to 0/0 only[b]	Towards/Away[c]
Basenji	3.4×10^{-4}	0.0023	0.0021	3.24
Dingo	1.0×10^{-4}	0.0010	0.0009	4.08
Israeli wolf	1.5×10^{-4}	0.0024	0.0013	4.34
Croatian wolf	1.3×10^{-4}	0.0034	0.0017	6.93
Chinese wolf	1.8×10^{-4}	0.0010	0.0012	4.50
Golden jackal	5.8×10^{-4}	0.0045	0.0032	5.35

[a] Frequency of all errors made in the direction of the reference, excluding errors in the opposite direction.
[b] Only considering errors towards the reference made at sites called heterozygous on the chip.
[c] Ratio of errors towards reference to errors away from reference.
