# Supplementary material for: Genome Sequencing Highlights the Dynamic Early History of Dogs
Source: PLoS Genet. 2014 Jan 16;10(1):e1004016. doi: 10.1371/journal.pgen.1004016 (PMC3894170; doi:10.1371/journal.pgen.1004016)
Supplement: Table S6 — Autosomal heterozygosity for six canid genomes. (PDF) [file pgen.1004016.s012.pdf]

Table S6. Autosomal heterozygosity for six canid genomes.

**Sample**	***N***	**Heterozygosity**[a]
Basenji	1213620321	$0.00086 \pm 1.65 * 10^{-6}$
Dingo	1412511207	$0.00057 \pm 1.25 * 10^{-6}$
Israeli wolf	1445995509	$0.00162 \pm 2.07 * 10^{-6}$
Croatian wolf	1387203642	$0.00143 \pm 1.99 * 10^{-6}$
Chinese wolf	1497066162	$0.00123 \pm 1.78 * 10^{-6}$
Golden jackal	1415330381	$0.00148 \pm 2.01 * 10^{-6}$

[a] Normal approximation binomial confidence interval.
