# Supplementary material for: Genome Sequencing Highlights the Dynamic Early History of Dogs
Source: PLoS Genet. 2014 Jan 16;10(1):e1004016. doi: 10.1371/journal.pgen.1004016 (PMC3894170; doi:10.1371/journal.pgen.1004016)
Supplement: Table S7 — Genome-wide pairwise sequence divergence, estimated using equation E8.1 (see Text S8) using all the genomic sites that passed the genomic quality filters outlined in Text S8. (PDF) [file pgen.1004016.s013.pdf]

**Table S7**. Genome-wide pairwise sequence divergence, estimated using equation E8.1 (see Text S8) using all the genomic sites that passed the genomic quality filters outlined in Text S8.

| | **Boxer** | **Basenji** | **Dingo** | **Israeli wolf** | **Croatian wolf** | **Chinese wolf** |
|---|---|---|---|---|---|---|
| **Boxer** | | | | | | |
| **Basenji** | 0.00087 | | | | | |
| **Dingo** | 0.00094 | 0.00097 | | | | |
| **Israeli wolf** | 0.00111 | 0.00105 | 0.00111 | | | |
| **Croatian wolf** | 0.00113 | 0.00110 | 0.00112 | 0.00101 | | |
| **Chinese wolf** | 0.00114 | 0.00111 | 0.00111 | 0.00106 | 0.00105 | |
| **Golden jackal** | 0.00211 | 0.00211 | 0.00212 | 0.00209 | 0.00209 | 0.00210 |
