# Supplementary material for: Genome Sequencing Highlights the Dynamic Early History of Dogs
Source: PLoS Genet. 2014 Jan 16;10(1):e1004016. doi: 10.1371/journal.pgen.1004016 (PMC3894170; doi:10.1371/journal.pgen.1004016)
Supplement: Table S8 — Genome-wide pairwise sequence divergence, estimated using equation E8.2 (see Text S8) using all the genomic sites that passed the genomic quality filters outlined in Text S8. (PDF) [file pgen.1004016.s014.pdf]

Table S8. Genome-wide pairwise sequence divergence, estimated using equation E8.2 (see Text S8) using all the genomic sites that passed the genomic quality filters outlined in Text S8.

	**Boxer**	**Basenji**	**Dingo**	**Israeli wolf**	**Croatian wolf**	**Chinese wolf**
**Boxer**						
**Basenji**	0.00087					
**Dingo**	0.00094	0.00100				
**Israeli wolf**	0.00111	0.00112	0.00116			
**Croatian wolf**	0.00113	0.00117	0.00116	0.00115		
**Chinese wolf**	0.00114	0.00117	0.00115	0.00118	0.00115	
**Golden jackal**	0.00211	0.00214	0.00214	0.00214	0.00214	0.00214
