# Supplementary material for: Genome Sequencing Highlights the Dynamic Early History of Dogs
Source: PLoS Genet. 2014 Jan 16;10(1):e1004016. doi: 10.1371/journal.pgen.1004016 (PMC3894170; doi:10.1371/journal.pgen.1004016)
Supplement: Table S9 — Estimates of the number of ABBA/BABA/BBAA sites in the six canid genomes. For each cell and each quartet comparison we report the number of ABBA/BABA/BBAA sites followed by the frequency of those three types of sites given that the site is bi-allelic with the two alleles found in two species each. The golden jackal was used as an outgroup in all comparisons. (PDF) [file pgen.1004016.s015.pdf]

Table S9. Estimates of the number of ABBA/BABA/BBAA sites in the six canid genomes. For each cell and each quartet comparison we report the number of ABBA/BABA/BBAA sites followed by the frequency of those three types of sites given that the site is bi-allelic with the two alleles found in two species each. The golden jackal was used as an outgroup in all comparisons.

P1	P2	P3	ABBA Sites	BABA Sites	BBAA Sites
Basenji	Dingo	Croatian wolf	164211; 28.43%	162364; 28.11%	250958; 43.45%
Basenji	Dingo	Israeli wolf	158610; 27.18%	179656; 30.78%	245329; 42.04%
Boxer	Basenji	Croatian wolf	144942; 24.82%	146113; 25.02%	292896; 50.16%
Boxer	Basenji	Israeli wolf	157007; 26.71%	147991; 25.17%	282873; 48.12%
Boxer	Dingo	Croatian wolf	177485; 27.15%	176031; 26.93%	300095; 45.91%
Boxer	Dingo	Israeli wolf	176511; 26.50%	189294; 28.42%	300201; 45.07%
Croatian wolf	Israeli wolf	Boxer	226123; 34.16%	210897; 31.86%	224971; 33.98%
Croatian wolf	Israeli wolf	Dingo	213742; 32.78%	212876; 32.65%	225351; 34.56%
Croatian wolf	Israeli wolf	Basenji	205695; 35.29%	182191; 31.26%	194909; 33.44%
Basenji	Dingo	Chinese wolf	173366; 29.45%	162030; 27.52%	253270; 43.02%
Boxer	Basenji	Chinese wolf	149172; 24.91%	147273; 24.59%	302448; 50.50%
Boxer	Dingo	Chinese wolf	192400; 28.40%	175946; 25.97%	309223; 45.64%
Croatian wolf	Chinese wolf	Boxer	216145; 32.52%	219859; 33.08%	228675; 34.40%
Croatian wolf	Chinese wolf	Dingo	221737; 33.97%	212060; 32.49%	218959; 33.54%
Croatian wolf	Chinese wolf	Basenji	190706; 32.79%	191336; 32.90%	199502; 34.31%
Chinese wolf	Israeli wolf	Boxer	242452; 35.42%	222327; 32.48%	219803; 32.11%
Chinese wolf	Israeli wolf	Dingo	223003; 33.37%	232071; 34.73%	213209; 31.90%
Chinese wolf	Israeli wolf	Basenji	216213; 36.43%	191475; 32.26%	185855; 31.31%
Basenji	Dingo	Boxer	179362; 32.42%	216634; 39.16%	157265; 28.43%
Chinese Wolf	Croatian Wolf	Israeli Wolf	230181; 34.70%	208597; 31.44%	224601; 33.86%
