# Supplementary material for: Genome Sequencing Highlights the Dynamic Early History of Dogs
Source: PLoS Genet. 2014 Jan 16;10(1):e1004016. doi: 10.1371/journal.pgen.1004016 (PMC3894170; doi:10.1371/journal.pgen.1004016)
Supplement: Table S10 — Estimates of the number of ABBA/BABA/BBAA sites in the three G-PhoCS models analyzed. For each cell and each quartet comparison we report: 1) The number of ABBA/BABA/BBAA sites; 2) The frequency of those three types of sites given that the site is bi-allelic with the two alleles found in two species each and 3) the difference of that frequency in the simulations minus what is estimated in the data (when this difference is bigger than 1.5%, we highlight the cell in bold). The lower row of the table indicates the fit of the model to the data as estimated by equation 8.7 in Text S8. The golden jackal was used as an outgroup in all comparisons. (PDF) [file pgen.1004016.s016.pdf]

Table S10. Estimates of the number of ABBA/BABA/BBAA sites in the three *G-PhoCS* models analyzed. For each cell and each quartet comparison we report: 1) The number of ABBA/BABA/BBAA sites; 2) The frequency of those three types of sites given that the site is bi-allelic with the two alleles found in two species each and 3) the difference of that frequency in the simulations minus what is estimated in the data (when this difference is bigger than 1.5%, we highlight the cell in bold). The lower row of the table indicates the fit of the model to the data as estimated by equation 8.7 in Text S8. The golden jackal was used as an outgroup in all comparisons.

**Taxa**			**Fig. 5A model (Model where the dogs and wolves are each a separate clade)**			**Fig. 5B model (Regional domestication model)**			**Fig. 5C model (Origin of dogs from the Israeli wolf)**		
**P1**	**P2**	**P3**	**ABBA Sites**	**BABA Sites**	**BBAA Sites**	**ABBA Sites**	**BABA Sites**	**BBAA Sites**	**ABBA Sites**	**BABA Sites**	**BBAA Sites**
Basenji	Dingo	Croatian wolf	177596;<br>28.53%;<br>0.10%	180202;<br>28.95%;<br>0.84%	264624;<br>42.52%;<br>-0.94%	178773;<br>28.94%;<br>0.50%	177186;<br>28.68%;<br>0.57%	261870;<br>42.39%;<br>-1.07%	178434;<br>28.88%;<br>0.45%	177152;<br>28.67%;<br>0.56%	262289;<br>42.45%;<br>-1.00%
Basenji	Dingo	Israeli wolf	173506;<br>27.87%;<br>0.69%	191296;<br>30.72%;<br>-0.06%	257817;<br>41.41%;<br>-0.63%	173256;<br>27.83%;<br>0.65%	192556;<br>30.93%;<br>0.15%	256705;<br>41.24%;<br>-0.80%	173222;<br>27.82%;<br>0.64%	188792;<br>30.32%;<br>-0.46%	260580;<br>41.85%;<br>-0.18%
Boxer	Basenji	Croatian wolf	157926;<br>25.24%;<br>0.42%	158158;<br>25.28%;<br>0.26%	309616;<br>49.48%;<br>-0.67%	155013;<br>24.78%;<br>-0.04%	156346;<br>24.99%;<br>-0.03%	314275;<br>50.23%;<br>0.08%	158543;<br>25.42%;<br>0.60%	158872;<br>25.47%;<br>0.45%	306268;<br>49.11%;<br>-1.05%
Boxer	Basenji	Israeli wolf	168735;<br>26.93%;<br>0.23%	155221;<br>24.78%;<br>-0.40%	302524;<br>48.29%;<br>0.17%	165943;<br>26.52%;<br>-0.19%	155130;<br>24.79%;<br>-0.38%	304670;<br>48.69%;<br>0.57%	167349;<br>26.80%;<br>0.09%	155402;<br>24.89%;<br>-0.29%	301725;<br>48.32%;<br>0.20%
Boxer	Dingo	Croatian wolf	172541;<br>27.69%;<br>0.53%	175379;<br>28.14%;<br>1.21%	**275228;**<br>**44.17%;**<br>**-1.75%**	**148908;**<br>**23.69%;**<br>**-3.47%**	**148654;**<br>**23.64%;**<br>**-3.29%**	**331136;**<br>**52.67%;**<br>**6.76%**	172536;<br>27.92%;<br>0.76%	171583;<br>27.76%;<br>0.83%	**273917;**<br>**44.32%;**<br>**-1.59%**
Boxer	Dingo	Israeli wolf	173388;<br>27.77%;<br>1.27%	177664;<br>28.45%;<br>0.03%	273358;<br>43.78%;<br>-1.30%	**147173;**<br>**23.27%;**<br>**-3.24%**	**155660;**<br>**24.61%;**<br>**-3.82%**	**329753;**<br>**52.13%;**<br>**7.05%**	171562;<br>27.53%;<br>1.03%	175185;<br>28.11%;<br>-0.31%	276446;<br>44.36%;<br>-0.72%
Croatian wolf	Israeli wolf	Boxer	205879;<br>33.27%;<br>-0.89%	201724;<br>32.60%;<br>0.74%	211157;<br>34.13%;<br>0.14%	208604;<br>33.71%;<br>-0.44%	200215;<br>32.36%;<br>0.50%	209921;<br>33.93%;<br>-0.06%	208423;<br>33.80%;<br>-0.36%	**207350;**<br>**33.62%;**<br>**1.76%**	200941;<br>32.58%;<br>-1.40%
Croatian wolf	Israeli wolf	Dingo	203877;<br>32.96%;<br>0.18%	201160;<br>32.53%;<br>-0.13%	213431;<br>34.51%;<br>-0.06%	202216;<br>32.78%;<br>0.00%	202568;<br>32.84%;<br>0.19%	212020;<br>34.37%;<br>-0.19%	205800;<br>33.35%;<br>0.57%	209303;<br>33.92%;<br>1.27%	**201941;**<br>**32.73%;**<br>**-1.84%**
Croatian wolf	Israeli wolf	Basenji	215597;<br>34.74%;<br>-0.56%	197696;<br>31.85%;<br>0.59%	207361;<br>33.41%;<br>-0.03%	216547;<br>34.95%;<br>-0.35%	196012;<br>31.63%;<br>0.37%	207051;<br>33.42%;<br>-0.03%	216467;<br>35.11%;<br>-0.19%	**203118;**<br>**32.94%;**<br>**1.68%**	197038;<br>31.95%;<br>-1.49%
Basenji	Dingo	Chinese wolf	188009;<br>30.16%;<br>0.71%	177552;<br>28.49%;<br>0.96%	**257728;**<br>**41.35%;**<br>**-1.67%**	188470;<br>30.47%;<br>1.02%	174988;<br>28.29%;<br>0.77%	**254996;**<br>**41.23%;**<br>**-1.79%**	185253;<br>29.98%;<br>0.53%	173424;<br>28.06%;<br>0.54%	259312;<br>41.96%;<br>-1.06%
Boxer	Basenji	Chinese wolf	160801;<br>25.64%;<br>0.74%	158007;<br>25.20%;<br>0.61%	308245;<br>49.16%;<br>-1.34%	156840;<br>25.13%;<br>0.22%	155804;<br>24.97%;<br>0.37%	311426;<br>49.90%;<br>-0.60%	157369;<br>25.29%;<br>0.38%	159053;<br>25.56%;<br>0.97%	305845;<br>49.15%;<br>-1.35%

Boxer	Dingo	Chinese wolf	184167; 29.48%; 1.09%	170916; 27.36%; 1.40%	**269545; 43.15%; -2.48%**	**159174; 25.32%; -3.08%**	**144656; 23.01%; -2.96%**	**324831; 51.67%; 6.03%**	178856; 28.94%; 0.55%	168711; 27.30%; 1.33%	**270441; 43.76%; -1.88%**
Croatian wolf	Chinese wolf	Boxer	203311; 32.95%; 0.43%	202091; 32.76%; -0.32%	211562; 34.29%; -0.11%	200348; 32.66%; 0.14%	198041; 32.28%; -0.80%	215078; 35.06%; 0.66%	204468; 33.45%; 0.93%	203864; 33.35%; 0.27%	202947; 33.20%; -1.20%
Croatian wolf	Chinese wolf	Dingo	213747; 34.53%; 0.57%	196438; 31.74%; -0.75%	208747; 33.73%; 0.18%	209895; 34.22%; 0.25%	193324; 31.52%; -0.97%	210107; 34.26%; 0.71%	210931; 34.50%; 0.53%	201135; 32.90%; 0.41%	199265; 32.60%; -0.95%
Croatian wolf	Chinese wolf	Basenji	205710; 33.27%; 0.48%	201464; 32.58%; -0.32%	211167; 34.15%; -0.15%	201556; 32.84%; 0.05%	196880; 32.08%; -0.82%	215250; 35.07%; 0.77%	203801; 33.28%; 0.49%	204552; 33.41%; 0.50%	203964; 33.31%; -1.00%
Chinese wolf	Israeli wolf	Boxer	**208018; 33.51%; -1.91%**	205083; 33.04%; 0.56%	207667; 33.45%; 1.35%	210840; 34.03%; -1.38%	204758; 33.05%; 0.58%	203911; 32.91%; 0.81%	210065; 34.00%; -1.42%	209596; 33.92%; 1.45%	198217; 32.08%; -0.03%
Chinese wolf	Israeli wolf	Dingo	200720; 32.34%; -1.03%	215312; 34.69%; -0.04%	204645; 32.97%; 1.07%	200301; 32.34%; -1.03%	217224; 35.07%; 0.34%	201859; 32.59%; 0.69%	204194; 33.06%; -0.31%	217493; 35.21%; 0.49%	195969; 31.73%; -0.18%
Chinese wolf	Israeli wolf	Basenji	**216436; 34.81%; -1.62%**	202781; 32.61%; 0.35%	202571; 32.58%; 1.27%	217724; 35.14%; -1.29%	201865; 32.58%; 0.32%	199982; 32.28%; 0.96%	218547; 35.38%; -1.05%	204447; 33.10%; 0.84%	194752; 31.53%; 0.21%
Basenji	Dingo	Boxer	190695; 31.36%; -1.06%	242304; 39.85%; 0.69%	175036; 28.79%; 0.36%	**244189; 40.10%; 7.69%**	**219636; 36.07%; -3.08%**	**145058; 23.82%; -4.60%**	192265; 31.81%; -0.60%	237327; 39.27%; 0.12%	174739; 28.91%; 0.49%
Chinese Wolf	Croatian Wolf	Israeli Wolf	208874; 33.63%; -1.06%	203245; 32.73%; 1.28%	208912; 33.64%; -0.22%	206703; 33.38%; -1.32%	198457; 32.05%; 0.61%	214034; 34.57%; 0.71%	204824; 33.27%; -1.43%	200458; 32.56%; 1.12%	210316; 34.16%; 0.31%
		**Absolute Error:**		**0.4298**			**0.8219**			**0.4668**	
