# Supplementary material for: Genome Sequencing Highlights the Dynamic Early History of Dogs
Source: PLoS Genet. 2014 Jan 16;10(1):e1004016. doi: 10.1371/journal.pgen.1004016 (PMC3894170; doi:10.1371/journal.pgen.1004016)
Supplement: Table S11 — Estimation of post-divergence gene flow using the D Statistic [18]. The outgroup in all comparisons is the golden jackal. Statistical significance is evaluated using a two-tailed Z test, with the additional requirement that that absolute value of the Z-score to be ≥3. Significant tests and sample pairs showing evidence for post-divergence gene flow are shown in bold. (PDF) [file pgen.1004016.s017.pdf]

Table S11. Estimation of post-divergence gene flow using the D Statistic [18]. The outgroup in all comparisons is the golden jackal. Statistical significance is evaluated using a two-tailed Z test, with the additional requirement that that absolute value of the Z-score to be ≥3. Significant tests and sample pairs showing evidence for post-divergence gene flow are shown in bold.

P1	P2	P3	ABBA Sites	BABA Sites	D (%)	SE (%)	Z	p-value
Basenji	Dingo	Croatian wolf	164211	162364	0.57%	0.40%	1.42	0.16
**Basenji**	Dingo	**Israeli wolf**	158610	179656	-6.22%	0.51%	**-12.21**	**$2.79x10^{-34}$**
Boxer	Basenji	Croatian wolf	144942	146113	-0.40%	0.46%	-0.88	0.38
Boxer	**Basenji**	**Israeli wolf**	157007	147991	2.96%	0.52%	**5.64**	**$1.67x10^{-8}$**
Boxer	Dingo	Croatian wolf	177485	176031	0.41%	0.44%	0.94	0.35
**Boxer**	Dingo	**Israeli wolf**	176511	189294	-3.49%	0.52%	**-6.71**	**$1.96x10^{-11}$**
Croatian wolf	**Israeli wolf**	**Boxer**	226123	210897	3.48%	0.65%	**5.33**	**$9.86x10^{-8}$**
Croatian wolf	Israeli wolf	Dingo	213742	212876	0.20%	0.54%	0.38	0.71
Croatian wolf	**Israeli wolf**	**Basenji**	205695	182191	6.06%	0.62%	**9.74**	**$1.99x10^{-22}$**
Basenji	**Dingo**	**Chinese wolf**	173366	162030	3.38%	0.45%	**7.49**	**$6.76x10^{-14}$**
Boxer	Basenji	Chinese wolf	149172	147273	0.64%	0.41%	1.54	0.12
Boxer	**Dingo**	**Chinese wolf**	192400	175946	4.47%	0.44%	**10.14**	**$3.77x10^{-24}$**
Croatian wolf	Chinese wolf	Boxer	216145	219859	-0.85%	0.42%	-2.02	$4.32x10^{-2}$
Croatian wolf	**Chinese wolf**	**Dingo**	221737	212060	2.23%	0.44%	**5.10**	**$3.48x10^{-7}$**
Croatian wolf	Chinese wolf	Basenji	190706	191336	-0.16%	0.39%	-0.42	0.68
Chinese wolf	**Israeli wolf**	**Boxer**	242452	222327	4.33%	0.68%	**6.41**	**$1.43x10^{-10}$**
**Chinese wolf**	Israeli wolf	**Dingo**	223003	232071	-1.99%	0.56%	**-3.58**	**$3.48x10^{-4}$**
Chinese wolf	**Israeli wolf**	**Basenji**	216213	191475	6.07%	0.64%	**9.50**	**$2.02x10^{-21}$**
