# Supplementary material for: Genome Sequencing Highlights the Dynamic Early History of Dogs
Source: PLoS Genet. 2014 Jan 16;10(1):e1004016. doi: 10.1371/journal.pgen.1004016 (PMC3894170; doi:10.1371/journal.pgen.1004016)
Supplement: Table S12 — Main set of parameter estimates in the G-PhoCS analysis. (PDF) [file pgen.1004016.s018.pdf]

Table S12. Main set of parameter estimates in *G-PhoCS* analysis.

Parameter	Raw estimate ×10^4	Calibrated[a]	Parameter	Raw estimate ×10^4	Calibrated[a]
N_{BSJ}	1.1 (0.9–1.2)	2,639 (2,322–2,942)	$T_{ancDOG1}$	0.40 (0.36–0.44)	12,102 (10,932–13,074)
N_{DNG}	0.8 (0.7–0.8)	1,914 (1,760–2,080)	T_{ancDOG}	0.43 (0.39–0.46)	12,795 (11,736–13,701)
N_{ISW}	10.4 (8.6–12.3)	26,092 (21,611–30,732)	$T_{ancWLF1}$	0.45 (0.39–0.50)	13,389 (11,688–14,982)
N_{CRW}	4.6 (3.9–5.2)	11,427 (9,835–12,968)	T_{ancWLF}	0.45 (0.39–0.50)	13,455 (11,766–15,060)
N_{CHW}	2.2 (1.9–2.4)	5,426 (4,733–6,087)	T_{ancDW}	0.50 (0.46–0.53)	14,874 (13,875–15,900)
N_{GLJ}	7.8 (7.5–8.1)	19,446 (18,746–20,139)	T_{root}	13.3 (12.7–13.8)	398,262 (381,927–415,086)
$N_{ancDOG1}$	0.3 (0.0–0.8)	793 (66–1,990)	$m_{ISW\text{->}BSJ}$	0.18 (0.12–0.24)	
N_{ancDOG}	0.8 (0.3–1.3)	1,999 (701–3,200)	$m_{BSJ\text{->}ISW}$	0.07 (0.06–0.09)	
$N_{ancWLF1}$	0.6 (0.0–1.3)	1,393 (44–3,327)	$m_{CHW\text{->}DNG}$	0.03 (0.00–0.06)	
N_{ancWLF}	5.1 (0.4–10.0)	12,627 (981–24,991)	$m_{DNG\text{->}CHW}$	0.04 (0.02–0.06)	
N_{ancDW}	18 (17.7–18.3)	44,993 (44,205–45,812)	$m_{GLJ\text{->}ISW}$	0.00 (0.00–0.00)	
N_{root}	7.3 (6.5–8.0)	18,169 (16,311–19,990)	$m_{ISW\text{->}GLJ}$	0.05 (0.04–0.06)	
			$m_{GLJ\text{->}ancDW}$	0.02 (0.01–0.03)	
			$m_{ancDW\text{->}GLJ}$	0.99 (0.89–1.10)	

[a] Absolute parameter values are obtained by assuming an average mutation rate of m=1.0 ×10^{-8} mutations per site per generation and an average generation time of three years (see Text S9).
