# Supplementary material for: Genome Sequencing Highlights the Dynamic Early History of Dogs
Source: PLoS Genet. 2014 Jan 16;10(1):e1004016. doi: 10.1371/journal.pgen.1004016 (PMC3894170; doi:10.1371/journal.pgen.1004016)
Supplement: Table S13 — qPCR results for copy number at AMY2B for 52 breed dogs, 6 Dingoes, and 40 wolves representing their global distribution. (PDF) [file pgen.1004016.s019.pdf]

Table S13. qPCR results for copy number at *AMY2B* for 52 breed dogs, 6 Dingoes, and 40 wolves representing their global distribution.

Breed/Population	SampleID	RKW#	CN Calculated	CN Predicted	Min CN	Max CN	CN Range	Replicates Analyzed
Afghan Hound	ODL13172	14137	10.58	10	9.64	11.69	2.05	3
Afghan Hound	ODL10621	14117	7.80	8	7.62	7.99	0.37	2
Afghan Hound	ODL10626	14118	19.43	19	14.73	33.15	18.42	3
Afghan Hound	ODL10623	4385	12.91	13	12.07	14.09	2.02	3
Afghan Hound	ODL10624	4386	14.30	14	14.30	14.30	0.00	1
Africanis	9328	4156	12.47	12	10.16	15.31	5.16	2
Africanis	9334	4157	14.03	14	14.03	14.03	0.00	1
Akita	ODL12758	14130	7.66	8	5.08	9.58	4.50	3
Akita	ODL1103	4343	5.92	6	5.47	6.25	0.78	3
Akita	ODL1131	4344	6.42	6	6.13	6.72	0.58	2
Basenji	6968	4176	13.19	13	12.25	14.20	1.94	2
Basenji	ODL4222	4353	18.38	18	18.38	18.38	0.00	1
Basenji	ODL4226	4354	8.27	8	7.21	9.48	2.27	2
Basenji	Basenji1	5361	7.18	7	6.26	7.96	1.71	3
Basenji		13477	16.87	17	13.07	18.98	5.91	2
Beagle	5 Erica		5.54	5	5.11	5.95	0.84	3
Bulldog	8 Erica		14.18	14	12.60	15.96	3.36	2
Canaan Dog	11916	4168	11.38	11	11.12	11.65	0.52	2
Canaan Dog	11910	4163	6.28	6	5.14	7.26	2.12	3
Chihuahua	2 Erica		9.87	10	9.77	9.97	0.2	3
Chinese Crested	Sizzle	2701	15.47	15	15.47	15.47	0.00	1
Flat-coated Retrieved	4 Erica		11.94	12	10.64	13.83	3.18	3
Great Dane	11 Erica		17.49	17	16.44	18.61	2.17	2
Ibizan Hound	ODL1228	4349	17.69	18	15.32	20.43	5.11	2
Kuvasz	ODL7526	4368	17.30	17	16.81	17.81	1.00	2
Kuvasz	ODL7525	4367	7.39	7	6.25	9.71	3.46	3
Mastiff	ODL2297	4351	8.48	8	7.04	12.26	5.22	3

Mastiff	ODL2298	4390	11.56	11	9.95	13.94	3.99	3
Mastiff	6 Erica		7.99	8	6.81	8.90	2.09	3
New Guinea Singing Dog	Hali	4155	11.09	11	10.04	12.25	2.21	2
New Guinea Singing Dog	Tilley 8 & 15	4186	10.91	11	8.86	12.27	3.41	3
Pekingese	ODL1219	4348	15.21	15	11.26	19.16	7.90	3
Pekingese	ODL1212	4347	12.21	12	10.33	14.42	4.09	2
Pekingese	9 Erica		10.04	10	7.11	13.57	6.46	3
Phu Quoc	VII Phu Quoc	5363	14.82	15	12.81	17.14	4.33	2
Saluki	1 erica		22.64	23	22.64	22.64	0	1
Saluki	493	4182	16.64	17	16.64	16.64	0.00	1
Saluki	1052	4183	33.50	34	33.50	33.50	0.00	1
Samoyed	ODL14507	14141	5.35	5	5.20	5.63	0.43	3
Samoyed	7402-5	5358	12.62	13	11.39	13.99	2.6	2
Samoyed	ODL14503	14140	4.50	4	2.89	6.94	4.05	3
Samoyed	ODL14535	14142	17.15	17	17.15	17.15	0.00	1
Samoyed	SSAM-6	5356	14.46	14	14.46	14.46	0.00	1
Scottish Terrier	7 Erica		8.28	8	6.92	9.91	2.99	2
Shar Pei	ODL11439	14123	12.64	13	11.94	13.53	1.59	3
Shar Pei	ODL11440	14124	7.99	8	7.30	9.50	2.20	3
Shar Pei	ODL11448	14126	14.17	14	12.45	15.41	2.96	3
Shar Pei	ODL11441	14125	16.59	16	12.90	21.35	8.46	2
Siberian husky	ODL11489	14127	7.37	7	6.43	8.32	1.88	3
Siberian husky	3 Erica		2.53	3	2.05	3.05	0.99	3
Thai Dog	VI Thai dog	5362	8.84	9	7.52	10.55	3.03	3
Toy Poodle	10 Erica		9.94	10	9.60	10.59	0.99	3
Dingo	Yindi	5317	5.29	5	4.61	5.89	1.28	3
Dingo	FC-4164	5316	2.51	2	1.98	3.60	1.62	3
Dingo	Pickle	5318	1.77	2	1.09	2.74	1.64	3
Dingo	Mustard	5319	2.04	2	1.90	2.29	0.39	3
Dingo	N723		1.94	2	1.47	2.41	0.95	3
Dingo		13760	2.72	3	2.52	2.91	0.39	3
Wolf (China)	BK14	14170	2.96	3	2.59	3.65	1.05	3
Wolf (China)	BK5	14172	2.94	3	2.34	3.40	1.06	3

Wolf (China)	12	645	4.09	4	3.64	4.70	1.06	3
Wolf (China)	bk05	13448	2.23	2	1.95	2.73	0.78	3
Wolf (China)		3916	1.89	2	1.76	2.02	0.25	2
Wolf (India)		3942	2.75	3	2.50	3.06	0.56	3
Wolf (Israel)	B	1413	4.26	4	2.71	7.94	5.23	3
Wolf (Israel)	1	1415	1.69	2	1.12	2.14	1.02	3
Wolf (Israel)	44	1420	4.51	4	4.50	4.51	0.00	2
Wolf (Israel)	11122	2580	2.02	2	1.93	2.11	0.18	3
Wolf (Italy)	W525	2785	1.78	2	1.42	2.02	0.60	3
Wolf (Italy)	W794	2794	1.88	2	1.81	2.02	0.20	3
Wolf (Italy)	W755	2799	1.87	2	1.69	2.03	0.34	3
Wolf (Italy)	W509	2781	2.03	2	1.74	2.24	0.50	3
Wolf (Italy)	ITA50		1.96	2	1.64	2.48	0.84	3
Wolf (Russia)	W295	1983	2.94	3	2.35	3.81	1.46	3
Wolf (Russia)	W780	1992	1.88	2	1.63	2.08	0.45	3
Wolf (Russia)	W523	1997	2.48	2	1.88	2.87	0.99	3
Wolf (Russia)	W437	2004	2.69	3	2.16	3.55	1.39	3
Wolf (Russia)	W289	2006	1.92	2	1.78	2.00	0.23	3
Wolf (Russia)	W620	1974	1.60	2	1.52	1.68	0.16	3
Wolf (Spain)	WIB11086A		2.03	2	1.92	2.27	0.35	3
Wolf (Spain)	WIB11090		3.54	4	3.19	3.89	0.7	3
Wolf (Spain)	WIB13		1.83	2	1.60	2.02	0.42	3
Wolf (Spain)	WIB18		2.64	2	2.13	3.32	1.19	3
Wolf (Spain)	WIB19		2.44	2	1.90	3.51	1.61	3
Wolf (Spain)	WIB21		2.62	2	2.43	2.91	0.48	3
Wolf (Spain)	WIb57		1.85	2	1.67	2.09	0.42	3
Wolf (Spain)	WIb60		1.60	2	1.31	2.06	0.75	3
Wolf (Spain)	WIB64		1.78	2	1.42	2.12	0.7	3
Wolf (Spain)	WIb74a		3.72	4	3.53	3.86	0.33	3
Wolf (Spain)	WIB98		7.76	8	6.43	8.83	2.40	3
Wolf (Sweden)	3220		1.47	2	1.34	1.61	0.27	2
Wolf (Yellowstone)	260F	921	3.92	4	3.20	4.50	1.30	3
Wolf (Yellowstone)	292M	931	4.66	4	3.62	5.54	1.93	3

Wolf (Yellowstone)	383M	960	5.51	5	4.57	6.27	1.70	3
Wolf (Yellowstone)	040F	969	2.15	2	1.88	2.47	0.58	3
Wolf (Yellowstone)		1547	2.92	3	2.09	4.10	2.01	3
Wolf (Yellowstone)		1548	2.31	2	1.62	3.42	1.80	3
Wolf (Great Lakes)		2455	3.29	3	3.00	3.48	0.47	3
Red Wolf		7874	2.24	2	2.13	2.47	0.34	3
Coyote		2430	2.55	2	2.25	3.09	0.85	3
