# Supplementary material for: Genome Sequencing Highlights the Dynamic Early History of Dogs
Source: PLoS Genet. 2014 Jan 16;10(1):e1004016. doi: 10.1371/journal.pgen.1004016 (PMC3894170; doi:10.1371/journal.pgen.1004016)
Supplement: Text S1 — Information on samples chosen for genome sequencing. (PDF) [file pgen.1004016.s020.pdf]

S1 Samples

Rena M. Schweizer[1], Adam H. Freedman[1], Holly Beale[2], Elaine Ostrander[2], Robert K. Wayne[1], John Novembre[1]

[1]University of California, Los Angeles
Department of Ecology and Evolutionary Biology
Los Angeles, California, United States of America

[2]National Institutes of Health
Cancer Genetics Branch
National Human Genome Research Institute
Bethesda, Maryland, United States of America.

S1.1 Samples for High Coverage Sequencing

We chose specific samples for each lineage based upon our ability to obtain high molecular weight genomic DNA containing low levels of protein and RNA. Following phenol-chloroform extraction for a panel of samples for each lineage, we estimated DNA concentrations with a Nano-Drop 2000 spectrophotomer, selecting samples with >200 ng/uL concentration and a 260/280 ratio within an ideal range that indicates minimal protein contamination. We also estimated DNA quality and quantity with a Quant-iT PicoGreen dsDNA assay (Life Technologies). Gel electrophoresis was used to run out all selected samples on 1% agarose gels, after which we selected a sample containing a high molecular weight band (>1500bp, indicating intact genomic DNA), or the sample with the least amount of smearing. In order to eliminate DNA fragments <1000 bp in length, each sample was subjected to Ampure Bead cleanup following manufacturer protocol (Beckman Coulter Genomics). Following this step, we concentrated the DNA via ethanol precipitation, and repeated the Nano-Drop, PicoGreen, and electrophoresis protocols.

The final samples selected for our study (see Table S1.1) were then genotyped with a species-diagnostic SNP panel in order to rule out the possibility that any were interspecies hybrids. We have developed a panel of 26 diagnostic SNP genetic markers that are able to distinguish between the gray wolf (*Canis lupus*), the dog (*Canis lupus familiaris*), the Coyote (*Canis latrans*), and their first and second generation hybrids [1]. These 26 SNPs (17 resolving wolf vs. dog, 2 resolving dog vs. coyote, and 7 resolving coyote vs. wolf) were identified and validated in a panel of 832 dogs, 180 gray wolves and 53 coyotes analyzed on the Affymetrix Canine SNP v2. microarray of 127,000 SNP markers [2,3]. The 26 SNP markers were assayed for the final sample set of DNA samples using high resolution melting (HRM) SNP detection analysis [4] on a Roche 480 LightCycler quantitative PCR instrument, along with sets of pure gray wolf, domestic dog, and coyote control standards that were homozygotes for the specific SNP diagnostic alleles. The HRM results for each of our final samples were compared to the control standards for each SNP. The basenji, dingo, Israeli wolf, Croatian wolf, and Chinese wolf did not have any alleles that suggested they were interspecies hybrids (results not shown). The golden jackal was further tested by HRM genotyping of 7 SNPs that distinguish golden jackals and coyotes from wolves (as above), and sequencing of cytb [5] (Koepfli, in preparation). No wolf SNP alleles were observed in the golden jackal (results not shown).

Table S1.1. Sample origins, and sequencing effort by sample, platform, and library.

Sample	Sample ID	Sample Origin	Sex	SOLiD LMP[a]	SOLiD fragment	HiSeq[b]
Basenji	RKW 13764	Bethesda, MD, USA	M	1	—	1
Dingo	RKW13760	Bargo Dingo Sanctuary, Australia	M	1	2[c]	1
Israeli wolf	RKW13759	Neve Ativ, Golan Heights, Israel	F	1	1[d]	1
Chinese wolf	RKW13451	San Diego Zoo, CA, USA	F	—	—	3
Croatian wolf	RKW 3919	Perković, Croatia	F	1	1[d]	1
Golden jackal	RKW 1332	Tel Aviv, Israel	F	2	1.75[d]	1

[a] Number of slides, long mate pair, 1.5kb insert, 50bp per end

[b] Number of lanes, paired end 400bp insert, 100bp per end

[c] Number of slides, 75bp

[d] 50bp

The golden jackal cytb sequence was compared to the Indian golden jackal sequence in Genbank (accession no. AY291433) and against sequences for pure gray wolves, dogs, coyotes, Ethiopian wolves, side-striped and blacked jackals and African wild dogs. It grouped closely with the Indian golden jackal. The sex of each sample whose sex was previously unknown or only suggested from field observation was tested in a PCR reaction using the DBX6 and DBY7 markers from [6]. The basenji, whose sex was known, was included as a control.

S1.2 12 Dog Breeds for Moderate Coverage Illumina Sequencing

We utilized data from an ongoing companion study (Beale et al., unpublished data) in the form of moderate coverage sequencing of 12 additional dog breeds, selected to represent the phenotypic and phylogenetic ranges of contemporary domestic dog breeds. Sequenced breeds were: Beagle, Bulldog, Chihuahua, Chow Chow, Flatcoated Retriever, Great Dane, Mastiff, Pekingese, Saluki, Scottish Terrier, Siberian Husky, and Toy Poodle. Three of these breeds—Chow Chow, the Siberian Husky, and Saluki—were previously found to be basal in phylogenetic studies [3,7]. Blood samples were obtained with the consent of dog owners at American Kennel Club (AKC)-sanctioned dog shows, specialty events, breed clubs, and veterinary clinics using a protocol approved by the NIH Institutional Animal Care and Use Committee (IACUC).

Briefly, DNA was extracted as previously described [8], and libraries were prepared and sequenced on an Illumina Hi-Seq 2000 according to manufacturer protocols, producing 72-108 million reads per sample. Reads were aligned to Canfam3.1 with BWA version #0.5.9-r16 [9] and alignments were refined according to GATK best practices, using GATK version 1.5-11 (Best Practice Variant Detection with the GATK v3, 2012) [10]. Randomly selected reads were removed from alignments in all samples to normalize coverage, which reduced the original depths (ranging from 5.2x to 8.3x per dog) to an average of 5.3x (5.1x-5.5x per dog).

References

1. VonHoldt BM, Pollinger JP, Earl DA, Parker HG, Ostrander EA, et al. (2013) Identification of recent hybridization between gray wolves and domesticated dogs by SNP genotyping. Mamm Genome 24: 80-88.
2. vonHoldt BM, Pollinger JP, Earl DA, Knowles JC, Boyko AR, et al. (2011) A genome-wide perspective on the evolutionary history of enigmatic wolf-like canids. Genome Res 21: 1294-1305.
3. vonHoldt BM, Pollinger JP, Lohmueller KE, Han EJ, Parker HG, et al. (2010) Genome-wide SNP and haplotype analyses reveal a rich history underlying dog domestication. Nature 464: 898-902.
4. Wittwer CT, Reed GH, Gundry CN, Vandersteen JG, Pryor RJ (2003) High-resolution genotyping by amplicon melting analysis using LCGreen. Clin Chem 49: 853-860.
5. Irwin DM, Kocher TD, Wilson AC (1991) Evolution of the Cytochrome-B Gene of Mammals. J Mol Evol 32: 128-144.
6. Seddon JM (2005) Canid-specific primers for molecular sexing using tissue or non-invasive samples. Conserv Genet 6: 147-149.
7. Parker HG, Kim LV, Sutter NB, Carlson S, Lorentzen TD, et al. (2004) Genetic structure of the purebred domestic dog. Science 304: 1160-1164.
8. Parker HG, Kukekova AV, Akey DT, Goldstein O, Kirkness EF, et al. (2007) Breed relationships facilitate fine-mapping studies: A 7.8-kb deletion cosegregates with Collie eye anomaly across multiple dog breeds. Genome Research 17: 1562-1571.
9. Li H, Durbin R (2009) Fast and accurate short read alignment with Burrows-Wheeler transform. Bioinformatics 25: 1754-1760.
10. McKenna A, Hanna M, Banks E, Sivachenko A, Cibulskis K, et al. (2010) The Genome Analysis Toolkit: A MapReduce framework for analyzing next-generation DNA sequencing data. Genome Res 20: 1297-1303.
