# Supplementary material for: Genome Sequencing Highlights the Dynamic Early History of Dogs
Source: PLoS Genet. 2014 Jan 16;10(1):e1004016. doi: 10.1371/journal.pgen.1004016 (PMC3894170; doi:10.1371/journal.pgen.1004016)
Supplement: Text S2 — Details concerning multi-platform library construction and sequencing strategy, read alignment statistics, and sequencing depth of coverage. (PDF) [file pgen.1004016.s021.pdf]

S2 Sequencing

Adam H. Freedman[1], Kevin M. Squire[2], Vasisht Tadigotla[3], Clarence Lee[3], Timothy Harkins[3], Stanley F. Nelson[2], Robert K. Wayne[1], John Novembre[1]

[1]University of California, Los Angeles
Department of Ecology and Evolutionary Biology
Los Angeles, California, United States of America

[2]University of California, Los Angeles
Department of Human Genetics
Los Angeles, California, United States of America

[3]Life Technologies
Foster City, California, United States of America

S2.1 General Strategy

The goal of our sequencing strategy was to obtain ≥ 20x coverage across the mappable regions of the genome. Such coverage has been seen to give good resolution for genotyping heterozygous sites within single individual samples (e.g., 1000 Genomes Project Consortium 2010), and this proved to be true in our sample, as we found by validating our sequence-based genotype calls with array-based genotypes (Text S5). For our first 5 samples (Basenji, Dingo, Israeli wolf, Croatian wolf, Golden jackal), we generated short read data on primarily the SOLiD platform, and added at least one lane per sample of Illumina HiSeq. Subsequent to this sequencing, we took advantage of available high coverage data generated for the Chinese wolf (but only using the HiSeq platform), because of the benefits of adding a sample representing of an additional hypothesized domestication center.

S2.2 Library Construction and Sequencing

S.2.2.1 SOLiD 4 Library Preparation (non-ECC)

In all cases, the manufacturers protocols were closely followed, either using the following protocol guides, or earlier versions. For SOLiD protocols, we refer the reader to the Applied Biosystems SOLiD 4 System Library Preparation Guide (4445673 Rev. A, March 2010), and Applied Biosystems SOLiD 4 System Templated Bead Preparation Guide (4448378 Rev. B, March 2010). For EZ Bead-based template bead preparation protocols, see the EZ Bead user guides for the Applied Biosystems SOLiD EZ Bead Emulsifier (4441486 Rev. E, October 2011), Applied Biosystems SOLiD EZ Bead Amplifier (4443494 Rev. E, October 2011), and the Applied Biosystems SOLiD EZ Bead Enricher (4443496 Rev. E, October 2011).

SOLiD LMP library preparation—For the long mate-paired library, we followed the 2x50 mate-paired library protocol from the Applied Biosystems SOLiD 4 System Library Preparation Guide (March 2010). The same protocol was used for all LMP libraries. For each sample, we started with 20 ug of genomic DNA. The DNA was sheared with a HydroShear Standard Shearing Assembly at speed code 5 (SC5) for 20 cycles, for a target fragment size of 1-2kb. The sample was purified using PureLink columns from a SOLiD Library Column Purification Kit. The

purified DNA was end-repaired with End Polishing Enzymes 1 and 2 to convert the ends to 5'-phosphorylated blunt-ended DNA. LMP CAP adapters from the SOLiD Mate-Paired Library Oligos Kit were ligated the end-repaired DNA, and the DNA was column purified again. The LMP CAP adapter is missing a 5' phosphate from one of its oligonucleotides, which causes a nick on each DNA strand when the DNA is circularized. To remove unbound CAP adaptors, the DNA was run on a 1% agarose gel. The gel was cut to select 1.5Kb DNA fragments and purified using a SOLiD Library Quick Gel Extraction Kit. The DNA fragments were then circularized with a biotinylated internal adaptor from the SOLiD Mate-Paired Library Oligos Kit., and column purified. Plasmid-Safe ATP-Dependent DNase was used to eliminate uncircularized DNA, and resulting DNA was column purified again. All libraries consisted of more than 100 ng of circularized product at this point. The circularized DNA was treated with E. coli DNA polymerase I to translate the nick into the genomic DNA region and column purified. After nick-translation, the DNA was digested with T7 exonuclease (to create a single strand gap around the nick) and S1 nuclease (to cleave most of the library molecule from the circularized template), and column purified. The DNA was again treated with End Polishing Enzyme 1 and 2 for end-repair and phosphorylation of the 5' ends for subsequent ligation. To purify the library, the DNA molecules were bound to Dynabeads MyOne Streptavidin C1 beads, which binds specifically to the biotin-labeled internal adapter, and then separated with a Dynal magnet. P1 and P2 adapters from the SOLiD Mate-Paired Library Oligos Kit were ligated to the end-repaired DNA, and the molecules bound to streptavidin beads were washed and purified from ligation side-product. Again, the DNA was nick-translated with DNA polymerase I. The library was then trial amplified using Library PCR Primers 1 and 2 with the Platinum PCR Amplification Mix, and run on a 2% E-Gel EX Gel to determine the required number of PCR amplifications. All libraries required between 12 and 16 amplifications. The resulting library was size selected to between 250 and 350 bp using the SOLiD Library Size Selection gel, and was extracted and desalted using columns. The libraries were then quantitated using the SOLiD Library TaqMan Quantitation Kit, using a qPCR run on the MJ Research DNA Engine Opticon 2 Real-Time Cycler.

SOLiD Fragment Library Preparation—Fragment library preparation is much simpler than LMP library preparation. We started with 5ug of DNA, which was sheared to a range of 150-180 bp (before adapter ligation) using a Covaris S2 System. As above, DNA ends were repaired using End Polishing Enzymes 1 and 2, and column purified. P1 and P2 adapters were ligated, followed by another column purification. The DNA was run on a SOLiD Library Size Selection gel, and a section corresponding to the post-ligation size of 200-230bp was cut. The DNA was nick-translated with DNA Polymerase I, amplified for 2 PCR cycles, and then column purified. qPCR quantification showed a yield of 50-150ng of DNA.

SOLiD Templated Bead Preparation—Most next-generation technologies have a DNA template immobilization strategy, followed by template amplification. The strategy employed by SOLiD sequencing is bead-based template immobilization, followed by emulsion PCR. Here we describe bead preparation. In early 2011, Life technologies introduced their EZ Bead system, which greatly simplified this step of sequencing preparation. However, we were not able to take advantage of this for our early LMP libraries, so we describe both bead preparation steps below.

Manual Template Bead Preparation—Bead preparation was conducted according to the macro (4 ePCR Reaction) protocol described in the Applied Biosystems SOLiD4 System Templated Bead Preparation Guide (March 2010). Bead preparation consists of emulsion PCR (ePCR), followed by a wash, template enrichment, and 3' end modification. For the ePCR step, the oil phase, aqueous phase (DNA template), and beads (SOLiD P1 DNA Beads) for the emulsion were prepared separately according to the full-scale ePCR reaction protocol for 2x50 or fragment library, and the emulsion was performed using a ULTRA-TURRAX Tube Drive from IKA and transferred to a 96 well plate for ePCR thermal cycling. After thermal cycling, the emulsion was broken and the beads were washed with Bead Wash Buffer and TEX Buffer according to protocol. Beads were then quantitated using NanoDrop. Depending on the library, between 800 million and 1 billion beads were produced. Next, beads with full length templates were isolated via oligo hybridization using the P2 primer. The P2-enriched beads were extended with a Bead Linker and Terminal Transferase enzyme according to library protocol. These were quantitated with a Nanodrop ND-1000 and the bead concentration was calculated using a Work Flow Analysis (WFA).Bead yield was around 700-900 million templated beads per library.

SOLiD EZ Bead-based Preparation—The EZ Bead system automates the emulsion, amplification, and enrichment steps above with three instruments (the EZ Bead Emulsifier, EZ Bead Amplifier, and EZ Bead Enricher). We used this system for SOLiD fragment library construction, and found the preparation much easier and the bead yield similar.

S2.2.2 SOLiD Sequencing (non-Exact Call Chemistry)

The library templated beads were loaded onto a 1-well flow-cell at a density of roughly 750,000 beads/ul, and run on a SOLiD 4 Sequencer with SOLiD 4 sequencing reagents using 50+50 LMP sequencing, 75+50 ECC LMP sequencing, or 50+35 Fragment sequencing protocols.

S2.2.3 SOLiD 4 Library Preparation (ECC)

Fragment and long mate pair libraries were prepared for both Basenji and Dingo DNA following prescribed protocols for the SOLiD 4 system library preparation guide, which will be briefly described here.

All DNA was quantified using the Qubit dsDNA HS assay. Fragment libraries were constructed using 1 microgram of genomic DNA for each species. The DNA is sheared sonically into small fragments using a Covaris S2 system, with a mean fragment size of ~150 to 180 bp. The sheared DNA is end-repaired and purified, prior to ligation of SOLiD P1 and P2 library adaptors. The ligated DNA with 200-230 bp length is subsequently isolated using an Invitrogen E-Gel SizeSelect agarose gel. The selected DNA is nick translated and PCR amplified using appropriate primers for 9 cycles. The resulting fragment libraries were purified and quantified using an Agilent Bioanalyzer and the corresponding high sensitivity DNA kit.

Long mate pair libraries were generated from 25-30 micrograms of genomic DNA. Genomic DNA was sheared using a Digilab Hydroshear with a standard shearing assembly. The DNA is end-repaired and purified prior to ligation of LMP CAP adaptors, which are missing a 5' phosphate. The adapted DNA is purified and size-selected for a 1.5 kb insert size by running the DNA in a 1% agarose gel and excising the appropriate length as determined by using a 1 kb DNA ladder. The size-selected DNA is extracted from the agarose cut and circularized by ligating a biotinylated internal adaptor. Plasmid-Safe ATP-

Dependent DNase is used to isolate the desired circularized DNA product. Because of the missing 5' phosphates in the CAP adaptors, circularization of the DNA results in a nick on each strand. A nick translation is performed followed by digestion with T7 exonuclease and S1 nuclease, resulting in library molecules with the desired mate pair tags being cleaved from the circularized DNA template. DNA molecules were again end-repaired, and the desired DNA molecules with internal adaptors were isolated using Dynabeads MyOne Streptavidin C1 beads. SOLiD P1 and P2 adaptors were ligated onto the end-repaired DNA. As with the fragment libraries, the ligated DNA underwent nick translation and PCR amplified using appropriate primers, and quantified.

The clonal amplification of libraries through emulsion PCR was performed using the SOLiD EZBead system as prescribed.

S2.2.4 SOLiD ECC Sequencing

Sequencing was done using the SOLiD 4 system with Exact Call Chemistry (ECC), generating 75 bp and 2x50 bp reads for the fragment and mate par libraries respectively. The principles of ECC are based on standard techniques used in communication and data storage system to minimize measurement error through redundancy and employing different encoding schemes. Due to its ligation-based approach, SOLiD sequencing can leverage the use an additional probe set that complements the standard two-base encoding scheme.

S2.2.5 Illumina Paired-End Sample Preparation and Sequencing

For Illumina library preparation protocols, see the Illumina Paired-End Sample Preparation Guide (1005063 Rev. E, February 2011). For all samples except the Chinese wolf, genomic paired-end sequencing libraries with average insert size of 300-500 bp were constructed according the manufacturer's recommended protocol. Briefly, ~5 μg of purified genomic DNA was fragmented by sonication using the Covaris Adaptive Focused Acoustics (AFA) System. 3' and 5' overhangs of the recovered genomic DNA fragment were converted into blunt ends using T4 DNA polymerase and Klenow enzyme (New England Biolabs). After end repair, an 'A' base was added to the blunt phosphorlated DNA fragments using Klenow 3'->5' Exo- (New England Biolabs). The standard paired-end adaptors were ligated to the 'A' tailed DNA fragments using a Quick DNA ligation kit (New England Biolabs). The ligated products were separated on a 2% argrose gel and the desired DNA fragments were recovered from the gel by the QIAquick Gel Extration kit (Qiagen). After the initial denaturation at 98°C for 30 seconds, the PCR reaction was carried out for 8 cycles of 98°C for 10 sec, 65°C for 30 sec, and 72°C for 30 sec using Phusion DNA polymerase (Finnzymes). The final extension was for 5 min at 72°C. Libraries were sequenced on an Illumina HiSeq2000 following the manufacturer's standard cluster generation with a V2 Paired End Cluster generation kit, and sequencing protocol with TruSeq SBS sequencing reagents. Base calling was performed with the on-instrument computer using RTA version 1.7. For the Chinese wolf sample (carried out at BGI), the same protocols were carried out except for an additional library QC step using the Agilent 2100 Bioanlayzer and ABI StepOnePlus Real-Time PCR system.

S2.2.6 Data Generation

Sequencing runs were distributed across instruments, sequencing centers and dates, such that the chances for cross-contamination would be minimized (Table S2.2.1). This is important because

one of our objectives was to assess admixture between wild and domestic lineages, and such contamination has the potential to create spurious signals of gene flow.

Combining SOLiD and Illumina sequencing reads, we generated between 969 and 3366 million reads per sample (Table S1). We generated the most reads for the golden jackal, in order to achieve >20x coverage despite a high PCR duplication rate indicative of library simplification (probably due to partial degradation of the tissue sample). The smallest data set and lowest coverage were generated for the basenji, as downstream analyses belatedly revealed issues with a SOLiD single-end fragment library generated for that sample undetected by standard quality control metrics, forcing us to exclude an additional >10x of poor quality sequencing data. Overall, we were able to align 69 - 94% of reads to the boxer reference, leading 20 ~ 28 Gb (Basenji) to 71 Gb (Dingo) of uniquely aligned bases (Table S1). With the exception of the basenji, all samples were genotyped to > 20x coverage (Table S1), such that (with the exception of the basenji), > 80% and 60% of the genome was covered by at least 10 or 20 reads, respectively (Figure S2.2.1).

Table S2.2.1. Dates and institutions where sequencing was carried out.

Sample	Library	Library Location	Sequencing Dates	Sequencing Location
Basenji	LMP[a]	LT	02.17.11	LT
Basenji	HiSeq	UCLA	12.10.10	UCLA
Dingo	FRAG[a]	LT	01.14.11	LT
Dingo	LMP[a]	LT	02.15.11	LT
Dingo	HiSeq	UCLA	12.10.10	UCLA
Israeli wolf	FRAG	UCLA	08.27.10	UCLA
Israeli wolf	LMP	UCLA	06.25.10	UCLA
Israeli wolf	HiSeq	UCLA	09.08.10	Stanford
Croatian wolf	FRAG	UCLA	11.19.10	UCLA
Croatian wolf	LMP	UCLA	12.02.10	UCLA
Croatian wolf	HiSeq	UCLA	08.15.10	UCLA
Chinese wolf	HiSeq	BGI	03.02.12	BGI
Golden jackal	FRAG	UCLA	09.13.10; 01.20.11	UCLA
Golden jackal	LMP-1	UCLA	07.28.10	UCLA
Golden jackal	LMP-2	UCLA	11.23.10	UCLA
Golden jackal	HiSeq	UCLA	12.10.10	UCLA

[a] Samples sequenced using Exact Call Chemistry (see Text S2 for details).

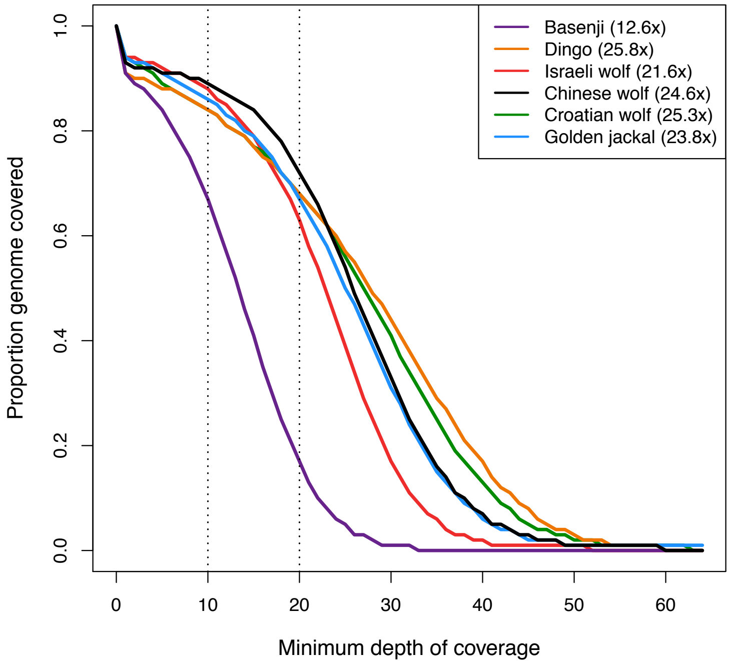

Figure S2.2.1. Proportion of the genome covered as a function of raw minimum depth of coverage. The vertical dashed lines at 10x and 20x are provided to aid interpretation.
