# Supplementary material for: Genome Sequencing Highlights the Dynamic Early History of Dogs
Source: PLoS Genet. 2014 Jan 16;10(1):e1004016. doi: 10.1371/journal.pgen.1004016 (PMC3894170; doi:10.1371/journal.pgen.1004016)
Supplement: Text S3 — Information on sequence alignment and genotyping pipeline methods. (PDF) [file pgen.1004016.s022.pdf]

S3 Genotyping Pipeline

Adam H. Freedman[1], Vasisht Tadigotla[2], Robert K. Wayne[1], John Novembre[1]

[1]University of California, Los Angeles
Department of Ecology and Evolutionary Biology
Los Angeles, California, United States of America

[2]Life Technologies
Foster City, California, United States of America

S3.1 Pipeline Design

We implemented a sequencing alignment and genotyping pipeline customized for combining SOLiD and Illumina HiSeq short read data (Figure S3.1), using aligners tailored to the specific platforms, then post-processing alignments using the Picard (http://picard.sourceforge.net) and Genome Analysis Toolkit (GATK) toolsets [1]. This pipeline converted short read raw data to .bam format alignment files [2], and from bam files to genotype files in .vcf format (http://www.1000genomes.org/node/101).

S3.1.1 Sequence Alignment

All short read data were aligned to the most current assembly of the dog genome (CanFam 3.0), generated from a boxer breed individual. CanFam 3.0 represents an early release of the update from CanFam 2.0, that was made publicly available by the Broad Institute but not added to NCBI or the UCSC Genome Browser as available downloads. CanFam 3.0 differs from the currently available CanFam 3.1 only in the length of N buffers at the beginning of each chromosome (3MB for autosomes in CamFam 3.0), and similarly, the length of those buffers between scaffolds assembled into chromsomes. To maximize the probability of proper alignment of short reads, data generated by the SOLiD and Illumina HiSeq platforms were each aligned using different alignment algorithm.

For SOLiD ECC reads, the ECC decoding pipeline was run offline using the image files (.spch) generated by the SOLiD instrument to generate corrected csfasta and qual files. The algorithm for generating corrected color calls is described at http://www3.appliedbiosystems.com/cms/groups/global_marketing_group/documents/generaldocuments/cms_091372.pdf

Both SOLiD ECC and non-ECC reads were aligned to Canfam 3.0 using the mapping and pairing modules in the BioScope1.2 pipeline. For the former, since this protocol used an early version of the ECC decoding the quality values (QV) were not properly calibrated in the mapped BAM files. QVs that were greater than 40 were reduced to 40. All Illumina reads were aligned to CanFam 3.0 using novoalign (version 2.07.11) (www.novocraft.com), with soft-clipping turned on.

Aligned reads from both sequencing platforms were merged and stored in bam format [2]. Reads corresponding to PCR duplicates were marked (and later removed) with Picard MarkDuplicates (picard.sf.net). Additional processing steps described below were then applied to the merged .bam files.

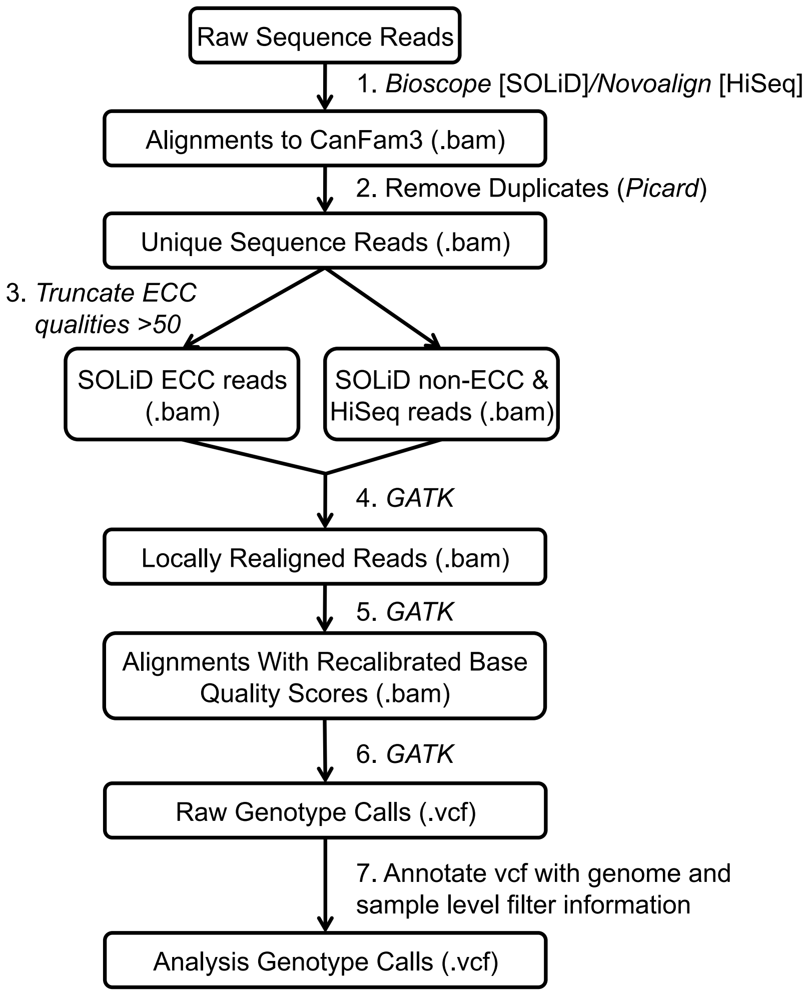

Figure S3.1. Schematic of sequence alignment and genotyping pipeline carried out separately for each lineage.

S3.1.2 Local Realignment

Short read alignment algorithms operate on each read independently, with the result that false SNVs can be detected in regions where repeated alignment errors occur across overlapping reads. A large proportion of such regions contain indels, with misalignment occurring most frequently for reads overlapping the indel near the read start or end. We used the GATK IndelRealigner [1] to perform local multiple alignment leading to a consensus indel call, and reducing the occurrence of false positive SNV sites. This three-step process entails first identifying suspicious intervals that may require realignment, followed by local realignment within these intervals, then 'rescuing' the mate pairing lost during the local realignment process, using the program Picard. Specific generic command lines are:

1) Interval detection

```
java -Xmx4g -Djava.io.tmpdir=GATKtemp  -jar Path/To/GenomeAnalysisTK.jar -T
RealignerTargetCreator -rf BadCigar -I Path/To/Infile/Infile.bam -L
ChromosomeName -o Path/To/IntervalsOutfile/intervals -R Canfam3.fa
```

2) Local realignment

```
java -Xmx4g -Djava.io.tmpdir=GATKtemp -jar
Path/To/GenomeAnalysisTK.jar -T IndelRealigner -I
Path/To/File/To/Realign/file.bam -o RealignedFile.bam -R Canfam3.fa  -
targetIntervals Path/To/Suspicious/Intervals/File/IntervalsFileName
```

3) Fix mate pair information

```
java -Xmx4g -Djava.io.tmpdir=GATKtemp -jar FixMateInformation.jar
INPUT=Path/To/Infile/infile.bam OUTPUT=Path/To/Outfile/Realigned_infile.bam
SO=coordinate VALIDATION_STRINGENCY=SILENT
```

S3.1.3 Base Quality Recalibration

Quality scores assigned to individual base calls are intended to reflect confidence in the specified nucleotide, but these scores may be weakly correlated with the actual probabilities of erroneous base calls. Important with respect to our study, the range of possible quality scores and the nature of quality score assignment differ substantially between SOLiD and Illumina sequencing platforms. To standardize quality scores across sequencing runs, libraries, and technologies, we performed empirical quality score recalibration using GATK. Recalibration involves three steps: 1) liberally defining a set of SNV-containing sites that are excluded from subsequent steps, 2) for all other sites, tabulating the frequency of base calls that are correct (i.e. consistent with homozygous-reference genotype) vs. incorrect as a function of covariates reflecting features of the underlying sequence context stratified by library/sequencing run, and 3) replacing the instrument-assigned quality scores with the genome-wide empirical error rates conditional on each unique covariate set. Step 1 was undertaken by genotyping in the same manner as below (see S3.1.4), but only requiring that genotypes containing an SNV had a genotype quality score $\geq$10.We used the three default covariates: read group (i.e. library), dinucleotide context, and position within the read. For SOLiD reads, reference bias introduced due to reference correction was removed by using the --solid_recal_mode SET_Q_ZERO and --solid_nocall_strategy PURGE_READ options to the walker. Specific generic command lines are:

1) Create recalibration table:

```
java -Xmx4g -Djava.io.tmpdir=GATKtemp -jar PathTo/GenomeAnalysisTK.jar -l
INFO -T CountCovariates -cov ReadGroupCovariate -cov CycleCovariate -cov
DinucCovariate --default_platform solid -I PathToInfile/infile.bam -
B:mask,VCF  PathToVariatnSitesToExclude/rod_file.vcf -R Canfam3.fa -recalFile
RecalibrationTable.csv --solid_recal_mode SET_Q_ZERO --solid_nocall_strategy
LEAVE_READ_UNRECALIBRATED
```

2) Generate recalibrated .bam file:

```
java -Xmx4g -Djava.io.tmpdir=GATKtemp -jar Path/To/GenomeAnalysisTK.jar -l
INFO -T TableRecalibration --default_platform solid -I
PathToInfile/infile.bam  --out outfile.bam -R Canfam3.fa -recalFile
RecalibrationTable.csv --doNotWriteOriginalQuals --solid_recal_mode
SET_Q_ZERO --solid_nocall_strategy PURGE_READ
```

S3.1.4 Base and Indel Genotyping with GATK

To call genotypes for our five novel canid genomes, we used the GATK Unified Genotyper (UG). UG employs a Bayesian genotype likelihood model that takes as input the base calls and associated quality scores for a locus, and emits the most likely genotype, the posterior probabilities that the locus is segregating and for the three possible genotypes. Only three genotype calls are possible because UG makes the simplifying assumption that a site is bi-allelic [1]. Although UG has multi-sample genotyping capabilities that enable estimation of population allele frequency across a set of samples, our focus on comparative genomics amongst evolutionarily distinct lineages (rather than, for example, variant discovery within a population of interest) led us to genotype each lineage separately. In addition, separate genotyping runs allowed us to specify separate priors on heterozygosity for each lineage, in keeping with known differences among wild and domestic canids. Specifically, priors were set based upon the heterozygosity estimates obtained by [3]. Because only one golden jackal was sampled in that study, for our golden jackal we used the value provided for wolves. To evaluate the sensitivity of our genotype calling to the assumed priors, we calculated the proportion of heterozygous genotype calls at these values, as well as for separate genotyping runs with values ± 50%. Priors had little effect on the frequency of heterozygous calls regardless of any hard threshold for minimum genotype quality (Figure S3.2). As a result, for each lineage we took as our final priors the average across the three runs, at genotype quality=20, the value we used as a sample-level hard filter (see Text S4). An example generic command line is as follows:

```
java -Xmx3g -jar Path/To/GenomeAnalysisTK.jar -T UnifiedGenotyper -l INFO --
genotyping_mode DISCOVERY --output_mode EMIT_ALL_CONFIDENT_SITES  -I $file -L
<chromosome_name> --min_base_quality_score 20 --
standard_min_confidence_threshold_for_emitting 0.0 --
standard_min_confidence_threshold_for_calling 0.0 --heterozygosity <lineage-
specific prior> -A GCContent -o outfile.vcf -metrics outfile.vcf_metrics.txt
-R Path/To/Canfam3.fa -dt NONE
```

Because we implemented our own conservative set of filters post-genotyping, we set both standard minimum confidence thresholds to zero.

Accurate calling and discovery of indel variants from next-generation sequencing is still subject to considerable uncertainty, and little prior information is available concerning the distribution of indels in the dog genome, let alone for other wild canid species. Furthermore, we had no way to validate indel calls at the genome-wide scale in a manner comparable to that available for SNV calls (see Text S4). Thus, we called indels only for use in the filtering out of SNVs proximate to them that might be false positives (see Text S4), accepting that the indel calls are only approximations. Our generic command line for indel calling using UG employed default settings

for indel_heterozygosity, min_indel_count_for_genotyping, and other indel-calling specific settings, as follows:

```
java -Xmx4g -jar Path/To/GenomeAnalysisTK.jar -T UnifiedGenotyper -l INFO -
glm INDEL --indel_heterozygosity 0.000125 --min_indel_count_for_genotyping 5
--genotyping_mode DISCOVERY --output_mode EMIT_ALL_CONFIDENT_SITES --
min_base_quality_score 20 --standard_min_confidence_threshold_for_emitting
0.0 --standard_min_confidence_threshold_for_calling 0.0 --heterozygosity
<lineage-specific prior> -I infile.bam -L <chromosome name> -o
indel_outfile.vcf  -metrics outfile.vcf_indel_metrics.txt -R
Path/To/Canfam3.fa
```

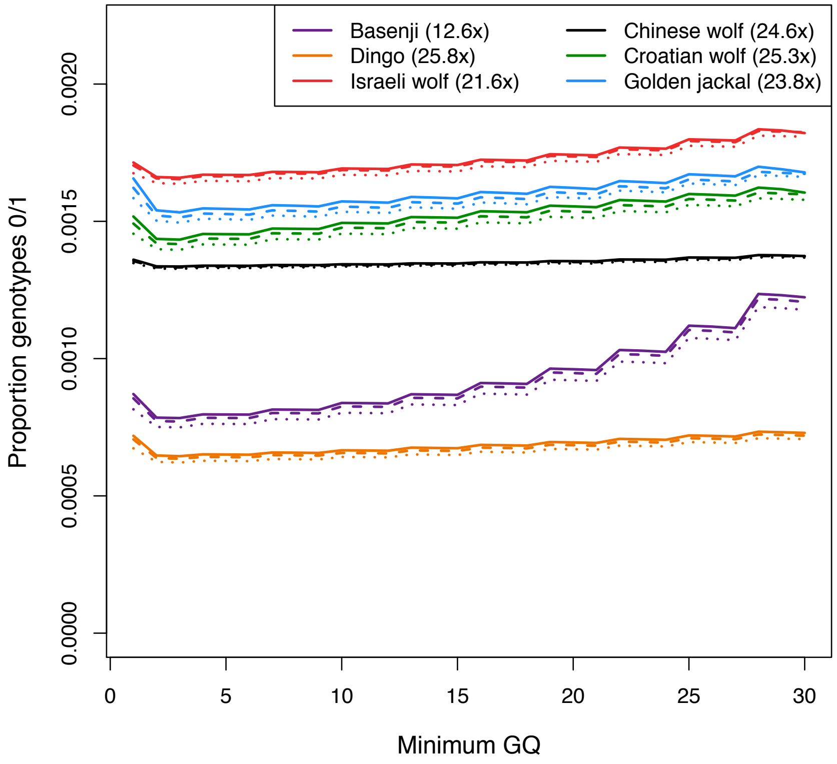

Figure S3.2. Proportion of genotypes typed as heterozygous using three different heterozygosity priors with UG, plotted against minimum genotype quality. Dashed, dotted and solid lines represent priors set at the mean, mean -50%, and mean +50% of nucleotide diversity estimates from Gray et al. [3].

References

1. DePristo MA, Banks E, Poplin R, Garimella KV, Maguire JR, et al. (2011) A framework for variation discovery and genotyping using next-generation DNA sequencing data. Nat Genet 43: 491-498.
2. Li H, Handsaker B, Wysoker A, Fennell T, Ruan J, et al. (2009) The Sequence Alignment/Map format and SAMtools. Bioinformatics 25: 2078-2079.

3. Gray MM, Granka JM, Bustamante CD, Sutter NB, Boyko AR, et al. (2009) Linkage Disequilibrium and Demographic History of Wild and Domestic Canids. Genetics 181: 1493-1505.
