# Supplementary material for: Genome Sequencing Highlights the Dynamic Early History of Dogs
Source: PLoS Genet. 2014 Jan 16;10(1):e1004016. doi: 10.1371/journal.pgen.1004016 (PMC3894170; doi:10.1371/journal.pgen.1004016)
Supplement: Text S4 — Information concerning quality filters applied to genotype data. (PDF) [file pgen.1004016.s023.pdf]

S4 Quality Filtering

Adam H. Freedman[1], Pedro Silva[2], Marco Galaverni[3], Robert K. Wayne[1], John Novembre[1]

[1]University of California, Los Angeles
Department of Ecology and Evolutionary Biology
Los Angeles, California, United States of America

[2]University of Porto
CIBIO-UP - Research Center in Biodiversity and Genetic Resources
Porto, Portugal

[3] Istituto Superiore per la Protezione e la Ricerca Ambientale
Laboratorio di Genetica
Ozzano dell'Emilia, Italy

S4.1.1 Filtering Conventions

In line with previous studies utilizing next-generation sequencing data, we developed a series of conservative data quality filters, implemented post-genotyping. Filters served two purposes. First, we sought to minimize the effects of sequencing and alignment errors that might bias downstream analyses [1,2]. Second, we sought to exclude regions of the genome that, irrespective of such errors, might show accelerated rates of evolution for reasons other than positive selection on the dog lineage, and might falsely appear as outliers in our selection scans; such regions might also be prone to misalignment of short reads. We established sets of criteria with which to filter at both the level of genomic position and individual lineages. *Genome feature filters* were applied to genomic positions based upon intrinsic features of the reference (Canfam3) and polymorphism across samples (i.e. tri-allelic and CpG sites), while *sample feature filters* were applied to individual lineage genotypes based upon features of the data underlying the corresponding genotype call. We annotated our VCF files according to whether genomic positions and samples passed the respective filtering criteria.

S4.1.2 Genome feature filters

Genomic positions in a VCF file were flagged as not passing the genome feature filter according to the following criteria.

1. *Repeat Regions.* We identified all genomic positions falling within repeat regions of the reference genome identified with RepeatMasker [3] and Tandem Repeat Finder (TRF) [4]. We annotated our VCF file according to the class of repeat detected, collapsing the output repeat classes into a reduced set of 14 classes: SINE, LINE, LTR, DNA, RNA, rRNA, scRNA, snRNA, srpRNA, tRNA, Satellite, Simple_repeat, Low complexity sequence, and Unknown. Because ancient repeats can make up a substantial portion of genomes, and because these regions will have diverged enough to allow accurate read mapping with short read alignment algorithms, we sought to retain these, and only mask out younger repeats prone to sequence misalignment. We considered that erroneous mapping of short reads to these regions should lead to increased frequency of heterozygous genotype calls, and plotted the frequency of heterozygote genotype

calls against divergence from the repeat libraries employed by RepeatMasker (Figure S4.1.1). We conservatively chose 25% divergence as our minimum repeat divergence threshold, as repeats in this interval show no increase in heterozygosity with decreasing repeat age.

2. *CpGs*. Mutation rates at CpG sites are substantially higher than non-CpG sites [5], so that regions enriched for CpGs may display elevated diversity and/or divergence leading to outliers in window-based analyses, independent from any demographic or selective forces germane to our investigation of domestication. If in any of our six lineages, a nucleotide that otherwise passed filter fell within a CpG dinucleotide, because at least some proportion of our data fell into that hyper-mutable site category, we flagged the genomic position.

3. *Copy Number Variants (CNVs).* When true CNVs are not included in a reference genome assembly, or when samples mapped to the reference contain novel CNVs, misalignment of paralogous reads is more probably, and can lead to false positive SNVs that can bias estimated

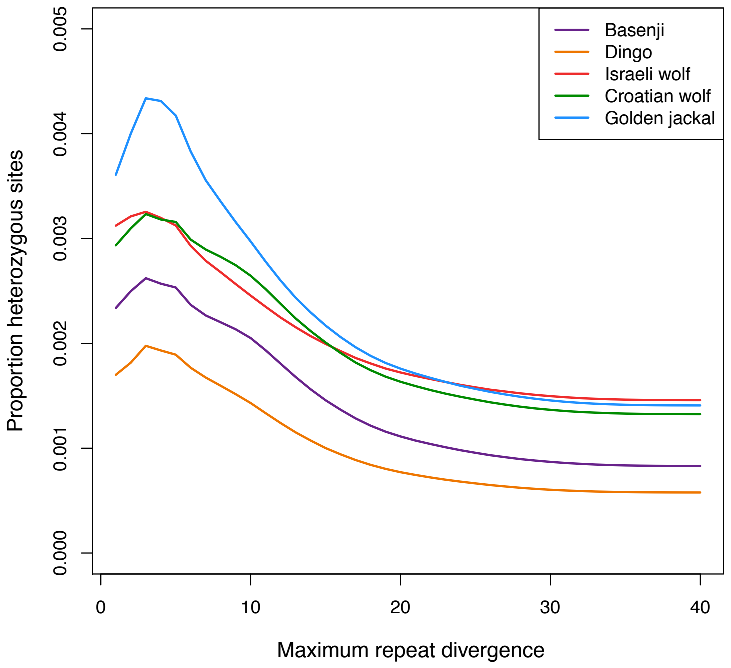

Figure S4.1.1. Proportion of heterozygous sites genotyped in repeat regions, as a function of the maximum divergence (of all repeats intersecting the genomic position of interest) between the observed repeat and the matching repeat motif used by RepeatMasker and Tandem Repeat Finder.

levels of polymorphism and divergence. To minimize the effects of such misalignment, we constructed a set of CNV regions to exclude from downstream analyses, by combining a set of

previously discovered CNVs reported in a diverse panel of dog breeds [6], and those we discovered directly from the short read data generated for our six canid lineages. See Text S5 regarding CNV detection methods.

4. *Triallelic sites.* Preliminary comparisons of genotypes from sequencing with those from the Illumina CanineHD BeadChip (see S.4.1 below), indicated triallelic sites were more prone to genotyping errors, and so these sites, while making up a relatively small fraction of the genome, were excluded.

We created genome feature filters at two levels: more stringent, using filters from all fours of the above categories, and less stringent, using only RM/TRF, CNV, and triallelic site filters. We used the more stringent filter for window-based analyses. We implemented the less stringent filtering for analyses of coding positions, as filtering out CpGs would a priori exclude a fraction of amino acids containing the CpG dinucleotide. We also reasoned that, because coding sequence is likely under evolutionary constraints, those constraints should reduce the disparity between mutation rates at CpG vs. non-CpG sites.

S4.1.3 Sample Feature Filters

1. *Proximity to Indel.* Short reads generated by next-generation sequencing platforms are prone to misalignment near indels, and attempts at local realignment around indels may not fully rectify this problem. As a result, these indel-proximate misaligned regions may be enriched for false positive SNVs. To account for this potential source of bias, for each sample we excluded any genotype containing an alternative allele relative to Canfam3 that was within 5bp (either up or downstream) of another SNV containing genotype within the same sample.

2. *Genotype Quality.* Genotype quality (GQ) metrics output by the GATK Unified Genotyper (UG) represent phred-scaled probabilities that the called genotype does not match the true underlying genotype, i.e $-10*\log_{10}(P[error])$. We chose a hard minimum GQ threshold of 20 (P[error]=0.01) based upon two considerations. First, we sought to minimize genotyping errors as measured by discordance with an independent, high quality genotype data set from the Illumina SNP chip (see S4.1). Second, we sought to balance the competing goals of retaining maximum genomic coverage while being able to correctly identify specific mutations of functional significance, particularly those fixed between dogs and wild canid species. Hard genotype quality thresholds may lead to undercalling of heterozygotes in samples with low or moderate coverage, but works well with those at >20x coverage [2]. All but one of our canid lineages were sequenced at >20x. Two additional lines of evidence support our use of a hard GQ threshold. First, the majority of all emitted genotypes have GQ >20 (Basenji 83.1%, Dingo 93.5%, Israeli wolf 95.6%, Croatian wolf 93.2%, Chinese wolf 98.9%, golden jackal 93.7%). Second, for our lowest coverage sample, the basenji, filtering on GQ appears to exclude more low quality homozygous genotypes, as the proportion of heterozygous calls shows an increasing trend with GQ above GQ=20 (Figure S3.2).

3. *Excess Depth of Coverage.* Extremely high depth of coverage relative to the genome-wide average likely indicates misalignment of reads generated from paralogous positions in the genome, particularly those containing CNVs. Indeed, excess depth of coverage is a typical metric used to define CNV regions, but CNV filtering alone will fail to detect finer-resolution CNV

signatures. Thus, we conservatively filtered all sites where depth of coverage exceeded twice the mean depth of coverage recorded for each lineage. GATK UG filters out reads that fail to meet certain criteria (see above). As a result, post-GATK filtering, depth of coverage may fall below our 2x threshold, even when the GATK filtering of hundreds of reads would indicate a region that may intrinsically be prone to read misalignment. Thus, our filtering on depth of coverage is based upon the number of reads overlapping a genomic position prior to imposition of the UG's internal filters.

4. *Clustered SNVs.* Within any sample, we excluded all SNV-containing genotypes falling within 5 bp of another SNV-containing genotype. In identifying clustered SNVs, to be conservative we required that proximate SNVs only have a minimum genotype quality of 10, rather than the 20 employed in our downstream evolutionary analyses.

Sample-level filters were employed as hard filters. For analyses involving estimation of genome-wide patterns of diversity, we used combinations of filters designated GF2 and SF (Table S5.1.1). For quantifying the number of dog and wolf specific variants, and for analysis of functional regions where the potential for elevated mutation rates at CpG sites should be constrained by functional consequences, we included CpG sites, equivalent to filters GF3 and SF (Table S5.1.1).

References

1. Jordan G, Goldman N (2012) The Effects of Alignment Error and Alignment Filtering on the Sitewise Detection of Positive Selection. Mol Biol Evol 29: 1125-1139.
2. Nielsen R, Paul JS, Albrechtsen A, Song YS (2011) Genotype and SNP calling from next-generation sequencing data. Nat Rev Genet 12: 443-451.
3. Smit AFA, Hubley R, Green P (1996-2010) RepeatMasker Open-3.0
4. Benson G (1999) Tandem repeats finder: a program to analyze DNA sequences. Nucleic Acids Res 27: 573-580.
5. Hodgkinson A, Eyre-Walker A (2011) Variation in the mutation rate across mammalian genomes. Nat Rev Genet 12: 756-766.
6. Nicholas TJ, Baker C, Eichler EE, Akey JM (2011) A high-resolution integrated map of copy number polymorphisms within and between breeds of the modern domesticated dog. BMC Genomics 12:414.
