# Supplementary material for: Genome Sequencing Highlights the Dynamic Early History of Dogs
Source: PLoS Genet. 2014 Jan 16;10(1):e1004016. doi: 10.1371/journal.pgen.1004016 (PMC3894170; doi:10.1371/journal.pgen.1004016)
Supplement: Text S5 — Methods and results related to the validation of genotype calls made from whole genome sequencing data for our six canid samples. (PDF) [file pgen.1004016.s024.pdf]

S5 Validation of Genotype Calls

Adam H. Freedman[1], Rena M. Schweizer[1], Pedro Silva[2], Marco Galaverni[3], Robert K. Wayne[1], John Novembre[1]

[1]University of California, Los Angeles
Department of Ecology and Evolutionary Biology
Los Angeles, California, United States of America

[2]University of Porto
CIBIO-UP - Research Center in Biodiversity and Genetic Resources
Porto, Portugal

[3] Istituto Superiore per la Protezione e la Ricerca Ambientale
Laboratorio di Genetica
Ozzano dell'Emilia, Italy

S5.1 Effective Genomic Coverage and Number Variants Discovered

Despite the fact that coverage for the basenji was substantially less than for the other canid samples, a large proportion of the genome was still genotyped with genotype qualities (GQ) ≥ 20. 77% of the genome was covered for the basenji at GQ ≥ 20, while coverage for the other five genomes ranged from 88% to 97% (Figure S5.1.1). The precipitous decline of coverage with increasing GQ for the basenji presumably reflects the difficulty for genotype callers of making high confidence calls with fewer reads.

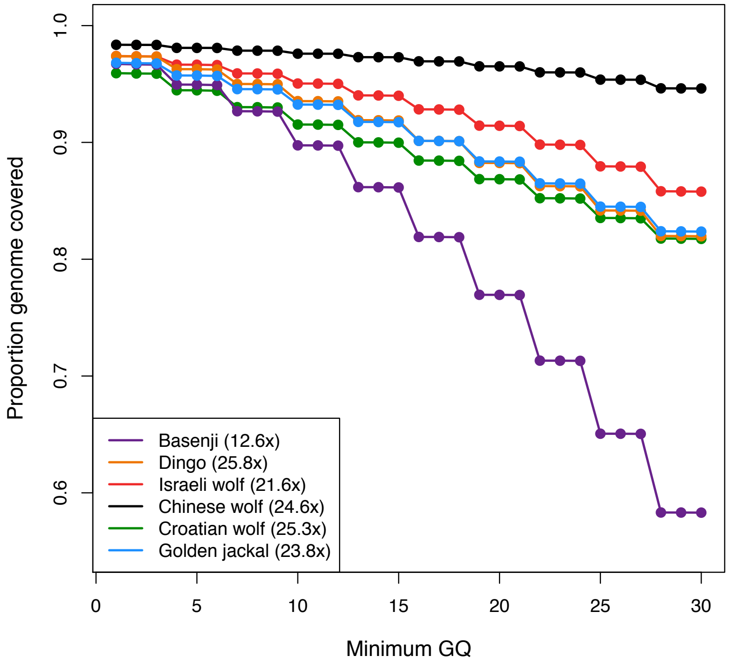

Figure S5.1.1. Genomic coverage per sample as a function of genotype quality, before imposing additional genome and sample level filters.

Imposing genome-level filters led to a large reduction in the amount of the genome available for genotyping. In order to access more sites in the boxer reference, instead of imposing a hard filter on repeat-masked sites, we chose to only filtered out young repeats to which it would be challenging to unambiguously align reads. This allowed us to gain an additional ~10% of the genome (GF1 vs. GF2, Table S2). Nevertheless, without filtering CpGs, genome level filtering (GF3) enabled us to genotype 68-69% of the genome for all six samples (Table S2). Using our combination of genome and sample level filters (GF3+SF), genomic coverage was 55% for the basenji and 63 - 68% for the other canids. Our filtering scheme reduced the discrepancy in coverage between the Basenji and the other samples observed from filtering on genotype quality alone (Figure S.5.1.1). This is not surprising, as difficult to align repeat regions that we filtered out would be harder to genotype effectively with lower coverage, as mapping qualities for individual reads in these regions would, on average, be lower than for more unique regions of the genome.

At present, high coverage genome sequences are not available for any wolf lineage. Looking across out novel wolf genomes, without any genome feature filtering we cover 94% of the boxer reference with at least one wolf genotype call, and even with such filters in place are able to provide genotypes for >60% of the non-N positions in the boxer reference for at least two different wolves (Figure S5.1.2).

After implementing genome and sample level filters (Text S4), we detected a large number of SNV sites relative to the boxer reference, ranging from 2.1-2.4 million for the 2 novel dog samples, 3.3-3.6 million for the 3 wolf samples, and 5.2 million for the golden jackal sample. (Table S2). Unsurprisingly, the golden jackal contained more unique SNV sites relative to the boxer reference than either dogs or wolves, and while wolves contained more unique SNV sites than dogs, a large portion of SNV sites were shared between dogs and wolves (Figure S5.1.3). Sites with no missing data in any lineage allowed us to classify variants as private to dogs, wolves, wild canids, or individual lineages, including 428,339 variants found in dogs but not in wolves, 867,656 variants in wolves that were absent in dogs, 1,524,761 variants shared between dogs and wolves, and 16,604 variants that were fixed between dogs and wild canids (Table S3).

S5.2 Comparison with Illumina Chip-Based Calls

The Illumina CanineHD BeadChip consists of >170,000 markers evenly spaced throughout the dog genome, ascertained from a diverse panel of dog breeds, and selected primarily from the Dog Genome Project 2.5 million SNP set. With call rates >99% and error rates less that 0.1%, the BeadChip data provide a high-quality benchmark against which to compare sequencing-based genotypes generated with our pipeline. Despite the high quality of the chip genotypes, we imposed an additional set of filters aimed at further reducing error rates. Specifically, based upon prior genotyping of a panel of 96 dogs, we only included positions with minor allele frequency >0, call rate ≥ 90%, and SNP heterozygosity < 120% of the expectation under Hardy-Weinberg equilibrium. A small number of positions were also excluded because they either a) produced a homozygous non-reference genotype for the original boxer used to build the reference genome (Canfam2), or b) produced genotypes inconsistent with those generated with the Affymetrix canine SNP array (v2). Because genomic positions on the chip are based upon the earlier Canfam2 reference, prior to comparison with our genotypes, we converted all chip coordinates to

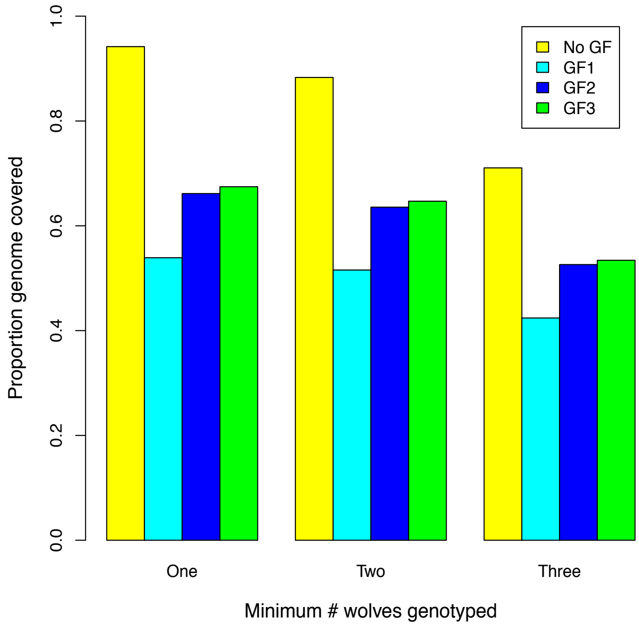

Figure S.5.1.2. Proportion of non-N bases in the boxer reference covered by ≥1 wolf as a function of different genome filters, and with the sample level filters in effect.

homologous coordinates on Canfam3.0 using the *liftover* utility available from the UCSC genome browser website (http://hgdownload.cse.ucsc.edu/admin/exe/).

Because all downstream analyses filter out, at a bare minimum, genotypes that do not pass genome (GF3) and sample (SF) level filters, we only compared filter-passing genotypes with those from the BeadChip. This comparison revealed a high degree of concordance (Table S4). An important benchmark with respect to genotype quality is the error rate at known heterozygous positions, as heterozygous genotype calls are more difficult to call correctly, and typically require higher coverage to do so, relative to either homozygous reference or homozygous non-reference positions. Heterozygote discordance rates were low and typical of other sequencing studies of similar coverage (Dingo, 0.010%, Israeli wolf 0.015%, Croatian wolf 0.018%, Chinese wolf 0.013%, Basenji 0.034%, Table S4). Although the heterozygous site error rate was higher for the golden jackal (0.058%), the frequency of chip heterozygous positions in the jackal was nearly half that of the lowest frequency observed in the other samples (jackal 7.0%; dogs and wolves, 12.0–26.8%), reducing the effect of such errors on downstream evolutionary analyses. The observed discordances showed evidence of bias towards the reference genome (Table S5), with errors towards the reference being anywhere from three to seven times higher than errors away from the reference. However, given the heterozygote discordance rates indicate the overall error rates are very low, and that the reference bias is similar across samples (Tables S4-S5), the effects on analyses should be small.

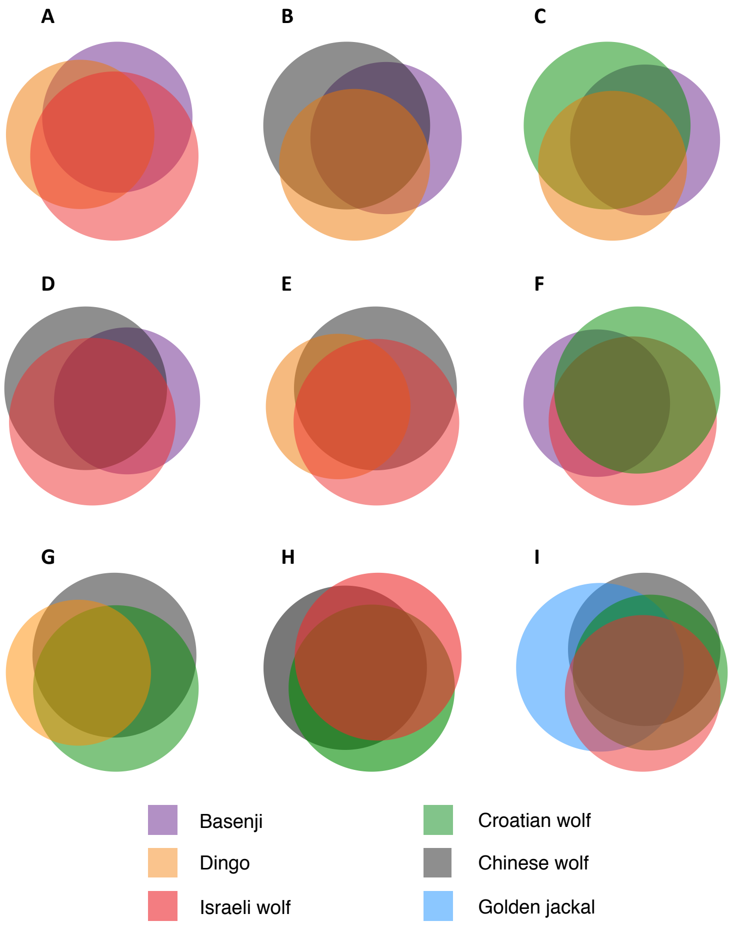

Figure S5.1.3. Euler diagrams showing relative frequency and proportional overlap of autosomal genomic positions containing an SNV. Size of circles is proportional to the number of genomic positions containing a variant allele relative to the boxer reference. As expected, wolves harbor more SNV sites than dogs, with much of the dog polymorphism overlapping with that in wolves, and jackals harbor a disproportionately large number of private alleles.

S5.3 Ti:Tv

The Ti/Tv ratio has been shown to be an important metric for evaluating the specificity of SNV calls, and the 1000 Genomes and other large-scale sequencing studies show a consistent ratio for whole genome data of ~ 2 [1-3]. Ti:tv for all six canid samples was close to the expected value of ~2 with a mean of 2.16 (range: 2.12 – 2.20; Table S5.3.1)

Table S5.3.1. Ti:Tv ratios for autosomes of 6 canid genomes.

Sample	Transitions	Transversions	Ti:Tv
Basenji	1934897	910072	2.126
Dingo	1995944	941029	2.121
Israeli wolf	2861812	1307555	2.189
Croatian wolf	2685632	1235497	2.174
Chinese wolf	2806363	1288970	2.177
Golden jackal	4422539	2015001	2.195

S5.4 Comparison of NGS Samples to CanMap Reference Samples

S5.4.1 SNP Selection

To assist in validating that our 6 NGS samples were accurate representatives of their respective taxon, we compared the genotypes of our sequenced samples to those from previous SNP genotyping studies, as follows. We chose a set of 10 Boxers, 13 Basenjis, 6 Dingoes, 22 Middle Eastern wolves, 9 European wolves, 10 Chinese wolves, and two golden jackals that were previously genotyped on the Affymetrix Canine version 2 genome-wide SNP mapping array ("CanMap" genotypes; see [4-6] for methods details). We chose a subset of 14,655 SNP positions that overlapped between the two array platforms and were validated using genotype data from our Croatian wolf sample, since it was genotyped on both platforms. This subset of SNPs was further selected so that all SNP positions passed our genome filter GF3, and SNP positions had to be non-missing in the basenji, since that was our lowest coverage sample. This final set of 11,763 SNPs (hereto referred to as the 12K data set) was extracted from our 6 NGS samples and coded as missing if the position did not pass filters (GF3, SF). Thus, for our 6 NGS samples, we constructed a set of genotypes extracted from sequences ("seq-based"), and a set of genotypes from the Illumina array ("Illumina array-based"). For some analyses, we used Illumina array-based genotype data from a second golden jackal individual. All genotypes were converted to PLINK format for subsequent analysis [7].

S5.4.2 Heterozygosity

Individual heterozygosity was calculated using PLINK [7] for the 12K SNP data set of CanMap samples and both the seq-based and Illumina array-based genotypes for the 6 focal samples of this study. The Illumina array-based genotypes for the second Israeli golden jackal were included. The heterozygosity of sequence-based and array-based genotypes of our 6 focal taxa agree very closely (Figure S5.4.1). The heterozygosity levels of the focal taxa are concordant with that of the CanMap samples, though Canmap samples of Basenjis and Middle Eastern wolves appear less heterozygous than the focal samples for this study.

S5.4.3 Principal Components Analysis

Principal components analysis was performed on the 12K data set to visualize the predominant patterns of genetic differentiation, and to verify that our 6 NGS samples clustered within the appropriate species or breed group, in accordance with findings from previous analyses [4,5]. Given that we wanted each individual represented only once within the PCA, we chose the sequencing-based genotypes for the 6 NGS samples, the Illumina array-based genotype data for the second golden jackal, and the CanMap data set. The first 15 PCs were analyzed using the SMARTPCA package within the EIGENSTRAT software [8].

Principal components analysis of individual SNPs separates Boxers, Basenjis, and Dingoes from the wild canids on the first two axes, which together explain over 30% of the variation (Figure S5.4.2). These groupings, and those on subsequent PCs (Figure S5.4.2) confirm previous findings [4,5]. In general, our 6 NGS samples are placed correctly within their respective taxon grouping for the first 15 PC axes. One exception occurs in PC9, which separates our golden jackal from the two CanMap golden jackals. This is most likely due to population-level differentiation within the golden jackals, since the CanMap samples are from Kenya and our sample is from Israel. We also found evidence for differentiation within the CanMap Chinese wolves on PC6, with the NGS samples clustering with one subgroup of the CanMap Chinese samples.

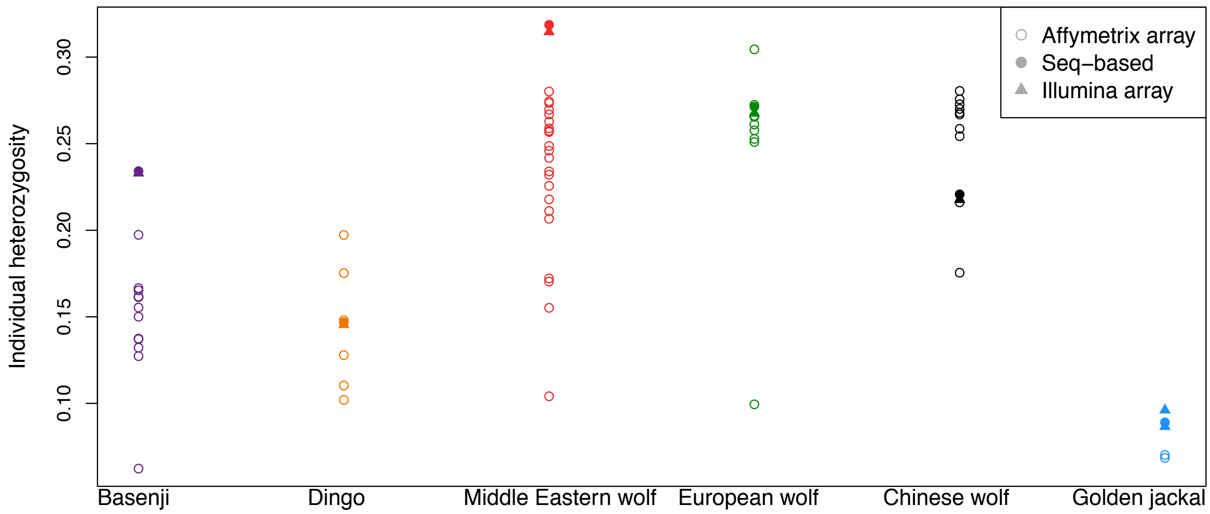

Figure S5.4.1. Heterozygosity estimates by individual for 12K SNP positions overlapping between sequencing-based genotype calls, and those from the Affymetrix and Illumina BeadChip SNP arrays. Sequencing-based genotypes show no apparent bias in heterozygosity. In all 6 taxonomic groups, the same individual was genotyped both via sequencing and the Illumina BeadChip platform, and the corresponding heterozygosity estimates are nearly identical in every instance.

S5.5 Comparison of Pairwise Distances to Previously Published Values

As a test of our data quality we compared pairwise genetic distances computed from our sample to those of a previous study of 12 exons in an overlapping set of lineages [9]. Lindblad-Toh *et al.* [9] defined a set of 12 exons that were used to reconstruct a phylogeny of 31 canid species. Those exons were selected based on the high percentage of bases that were informative in a phylogeny that included humans, dog, mouse and rat; that the mammal phylogeny reconstructed using those 4 mammalian species was consistent with their known phylogeny and that the exons could be amplified in the 31 canid lineages. Using those 12 exonic regions, they computed a matrix of pairwise distances, and reported pairwise distances for three species examined in our study. Those distances were: golden jackal - gray wolf (0.00062), golden jackal – domestic dog (0.00062) and gray wolf – domestic dog (0.00037).

From our sequencing data, we computed genetic distances among our six canid lineages for the same exonic regions (see Text S9.1 and Table S5.5.1). Mean pairwise distances computed from our samples were similar to the ones obtained by Lindblad-Toh *et al.* [9]: golden jackal - gray wolf (0.00077), golden jackal – domestic dog (0.00067) and gray wolf – domestic dog (0.00018). This concordance further supports the quality of our genotype calls.

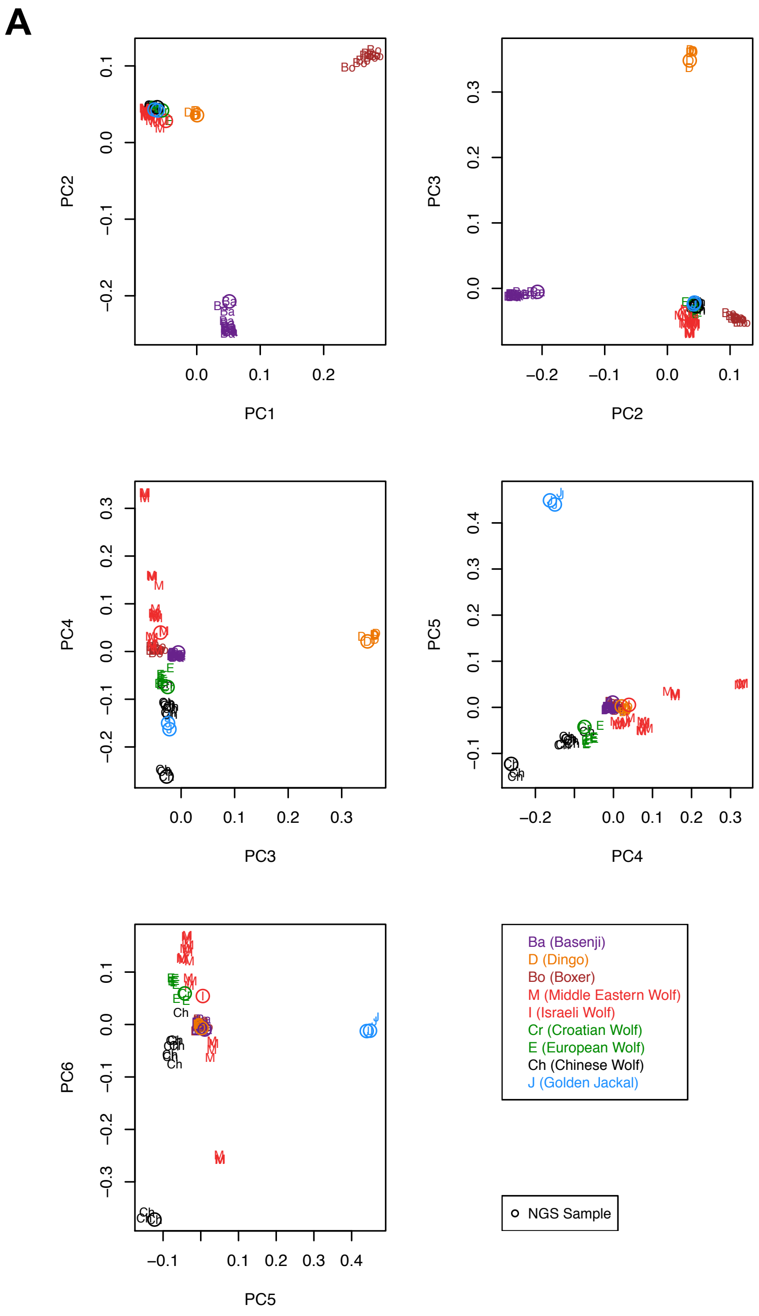
A
PC1
PC2
PC3
PC4
PC5
PC6
Ba (Basenji)
D (Dingo)
Bo (Boxer)
M (Middle Eastern Wolf)
I (Israeli Wolf)
Cr (Croatian Wolf)
E (European Wolf)
Ch (Chinese Wolf)
J (Golden Jackal)
NGS Sample

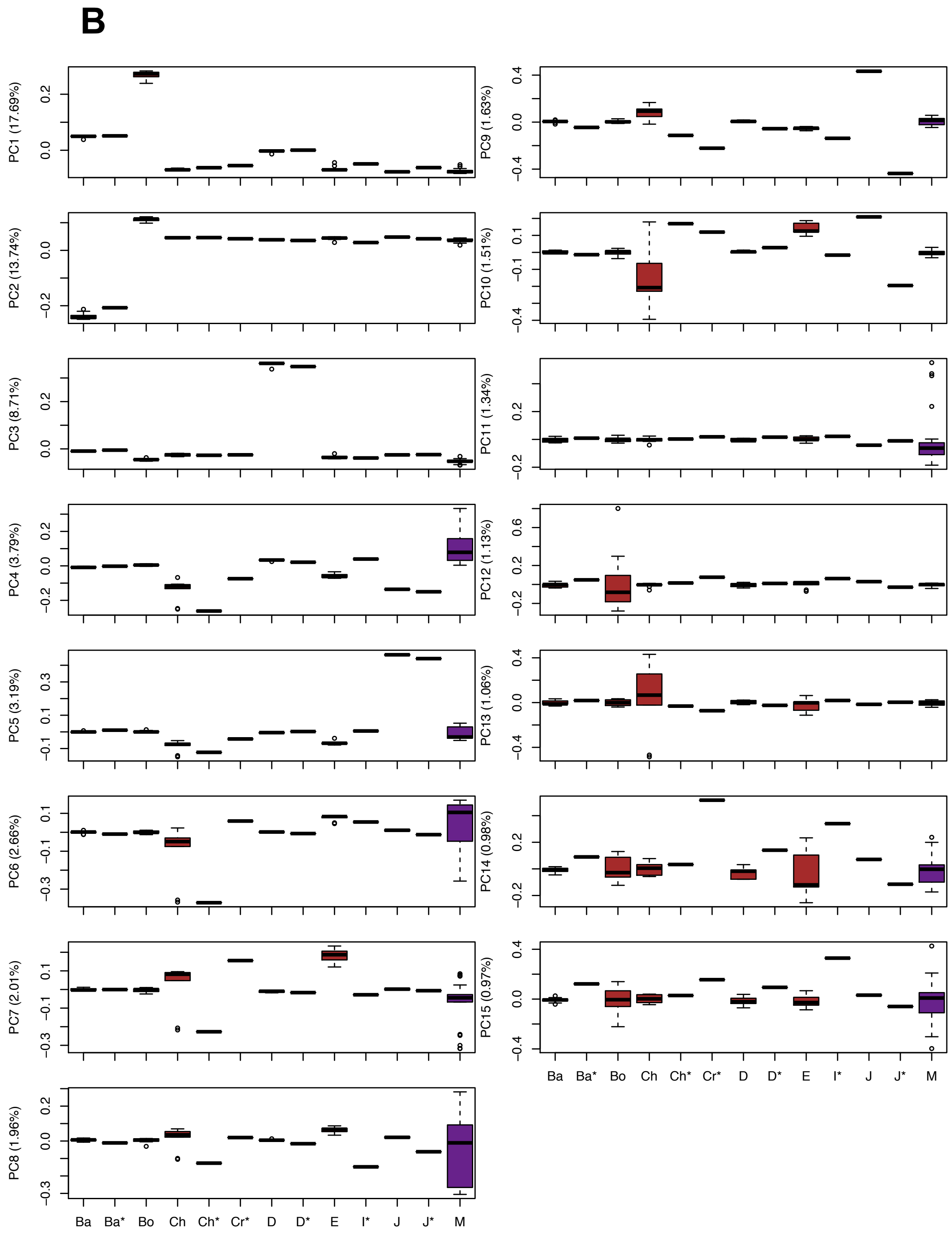

Figure S5.4.2. (A) PCA plot of ~ 12,000 overlapping SNPs between CanMap samples genotyped on the Affymetrix canid array, representing taxonomic groupings within which our NGS samples fall, compared to the same genotypes obtained from our sequencing data. (B) Individual boxplots of PCs 1-15, with asterisks indicating genotypes from next-generation sequencing.

.

Table S5.5.1. Pairwise sequence divergence for exonic regions sequenced by Lindblad-Toh et al. (2005).

	Boxer	Basenji	Dingo	Israeli wolf	Croatian wolf	Chinese wolf	Golden jackal
**Boxer**							
**Basenji**	0.00000						
**Dingo**	0.00011	0.00011					
**Israeli wolf**	0.00021	0.00021	0.00032				
**Croatian wolf**	0.00000	0.00000	0.00011	0.00021			
**Chinese wolf**	0.00021	0.00021	0.00032	0.00042	0.00021		
**Golden jackal**	0.00063	0.00063	0.00074	0.00084	0.00063	0.00084	

S5.6. Genome-wide Heterozygosity

To quantify genome-wide patterns of heterozygosity, for each genome we calculated the frequency of heterozygous genotype calls across all positions that passed the GF3 and SF filters (see Text S4). As expected, autosomal heterozygosity in dogs was lower in dogs than in wild canids, with levels in the former approximately half of those observed in the latter (Table S6.). Within dogs, the lower heterozygosity in the Dingo likely reflects the historical isolation and strong bottleneck experienced by that lineage. Conversely, the higher heterozygosity in the Basenji is due, in part, to admixture in wild canids (see main text and Text S8.4, S9). The low mean heterozygosity observed in the Chinese wolf relative to other wild canids was produced, in part, by large apparent runs of homozygosity revealed by analyses of 5MB-wide sliding windows (Figure S5.5.1). We formally identify runs of homozygosity (Figure S5.5.1) with the program PLINK v1.07 [8] using the following command line:

```
plink --tfile <input tfile> --homozyg --homozyg-snp 200 \
--homozyg-kb 10000  --homozyg-window-missing 30 \
--homozyg-window-het 10 --dog
```

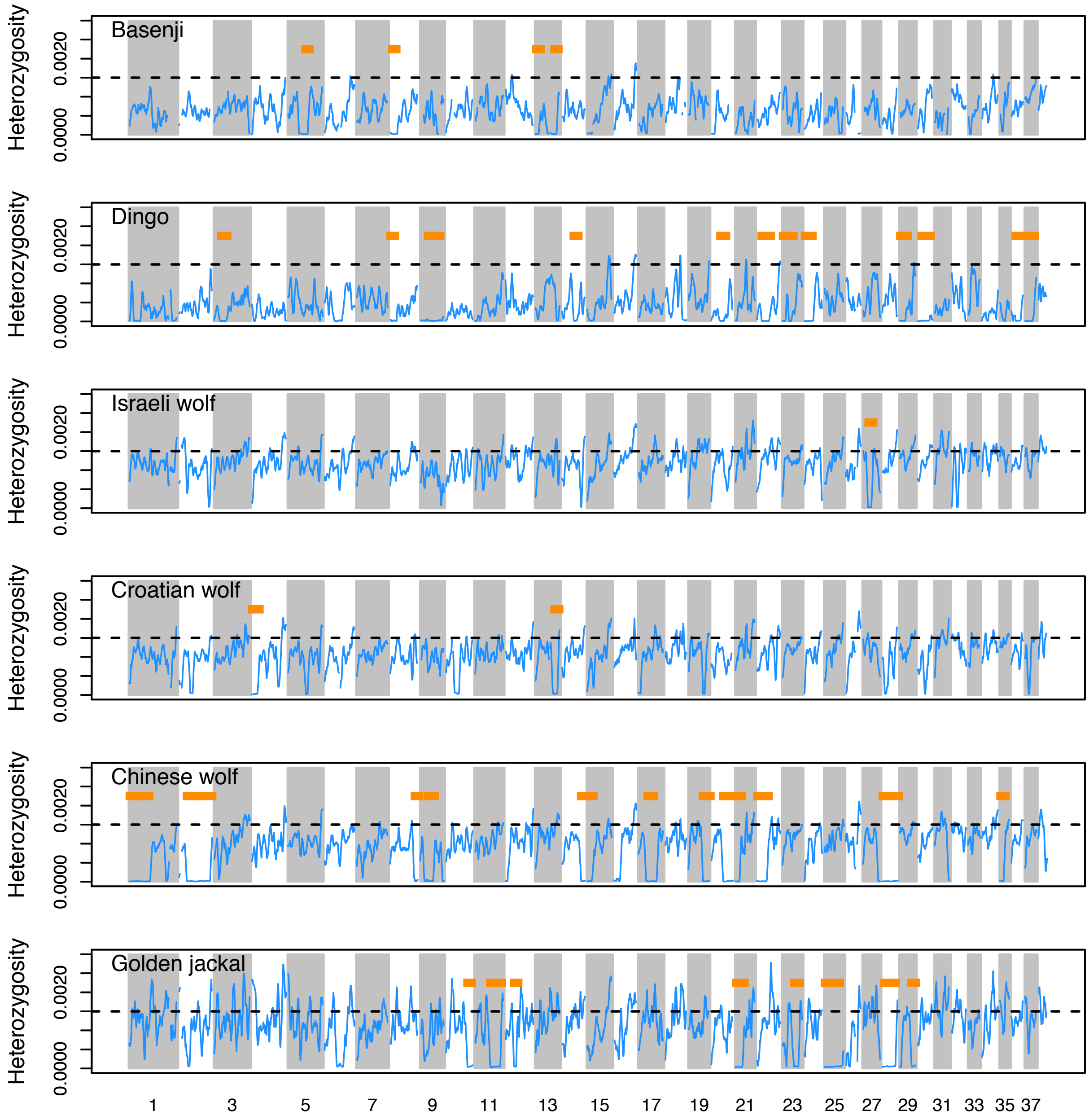

Figure S5.6.1. Genome-wide patterns of mean heterozygosity per sample in 5MB windows, sliding 1MB per step. A minimum of 2MB of genotypes passing GF3 and SF filters were required in order for a window to be displayed. Orange bars indicate runs of homozygosity detected using PLINK.

References

1. Ebersberger I, Metzler D, Schwarz C, Paabo S (2002) Genomewide comparison of DNA sequences between humans and chimpanzees. Am J Hum Genet 70: 1490-1497.
2. DePristo MA, Banks E, Poplin R, Garimella KV, Maguire JR, et al. (2011) A framework for variation discovery and genotyping using next-generation DNA sequencing data. Nat Genet 43: 491-498.

3. Altshuler D, Durbin RM, Abecasis GR, Bentley DR, Chakravarti A, et al. (2010) A map of human genome variation from population-scale sequencing. Nature 467: 1061-1073.
4. vonHoldt BM, Pollinger JP, Lohmueller KE, Han EJ, Parker HG, et al. (2010) Genome-wide SNP and haplotype analyses reveal a rich history underlying dog domestication. Nature 464: 898-902.
5. vonHoldt BM, Pollinger JP, Earl DA, Knowles JC, Boyko AR, et al. (2011) A genome-wide perspective on the evolutionary history of enigmatic wolf-like canids. Genome Res 21: 1294-1305.
6. Boyko AR, Quignon P, Li L, Schoenebeck JJ, Degenhardt JD, et al. (2010) A Simple Genetic Architecture Underlies Morphological Variation in Dogs. Plos Biology 8: e1000451.
7. Purcell S, Neale B, Todd-Brown K, Thomas L, Ferreira MAR, et al. (2007) PLINK: A tool set for whole-genome association and population-based linkage analyses. Am J Hum Genet 81: 559-575.
8. Price AL, Patterson NJ, Plenge RM, Weinblatt ME, Shadick NA, et al. (2006) Principal components analysis corrects for stratification in genome-wide association studies. Nat Genet 38: 904-909.
9. Lindblad-Toh K, Wade CM, Mikkelsen TS, Karlsson EK, Jaffe DB, et al. (2005) Genome sequence, comparative analysis and haplotype structure of the domestic dog. Nature 438: 803-819.
