# Supplementary material for: Genome Sequencing Highlights the Dynamic Early History of Dogs
Source: PLoS Genet. 2014 Jan 16;10(1):e1004016. doi: 10.1371/journal.pgen.1004016 (PMC3894170; doi:10.1371/journal.pgen.1004016)
Supplement: Text S6 — Methods and supporting results for structural variant calling. (PDF) [file pgen.1004016.s025.pdf]

S6 Structural Variant Calling

Belen Lorente-Galdos[1], Farhad Hormozdiari[2], Can Alkan[3], Tomas Marques-Bonet[1,4]

[1]Institut de Biologia Evolutiva (UPF-CSIC)
Departament de Ciències Experimental i de la Salut
Parc de Recerca Biomèdica de Barcelona
Dr. Aiguader 88, 08003 Barcelona

[2]University of California, Los Angeles
Department of Computer Science
4732 Boelter Hall
Los Angeles, CA 90095

[3]Bilkent University
Department of Computer Engineering
Ankara, 06800 Turkey

[4]ICREA. Institut Catala de Recerca i Estudis Avançats.
Catalonia, Spain

S6.1 Genome-wide Structural Variant Detection

We used whole-genome shotgun paired-end sequence data generated with both Illumina and Applied Biosystems SOLiD platforms from the genomes of six canid samples (including a additional Basenji only sequenced to low coverage on the Illumina platform, but excluding the Chinese wolf), to estimate the fraction of the genome with segmental duplications. Our goal was to determine potentially duplicated regions to filter out for the final SNP call set.

We identified the segmental duplication (SD) content in these genomes using the Whole-genome Shotgun Sequence Detection (WSSD) approach [1]. This strategy is based on determining regions with a significant excess of depth of coverage. Briefly, WGS reads are allowed to map to multiple locations to a reference genome, and therefore we expect that paralogous copies map into all locations. Highly identical duplicated genomic regions would be detected with an excess of depth of coverage. In our case, we used the dog assembly (canFam2) downloaded from the UCSC Genome Browser. Repeats detected by RepeatMasker and simple tandem repeats with period smaller than 12 detected by the Tandem Repeat Finder were pre-masked. We aligned the Illumina reads allowing 94% of sequence identity using mrFAST v2.0.0.5 [2] and SOLID reads with drFAST v0.0.0.3 [3].

We calculated the absolute copy numbers of non-overlapping windows of 1 kb of unmasked sequence using mrCaNaVaR version 0.31 (http://mrcanavar.sourceforge.net/). We identified SDs as regions with at least 5 consecutive windows with a copy number higher than 2.5. We detected between 1,379 and 1,413 SD segments larger than 10 kb in the five genomes we analyzed. These regions comprise 52.77 to 55.01 Mb in total that correspond to 2.09% to 2.17% of the reference assembly (Table S6.1.1).

The results are highly similar to the results we obtained using the SOLiD data with the same analysis protocols described above (Table S6.1.2). However, these results are likely to be conservative compared to a previous study [4] where 4.11% of the dog

Table S6.1.1. Segmental duplications detected as regions with at least 5 consecutive non-overlapping windows with a copy number higher than 2.5 from Illumina reads.

		**>10kb**		**>20kb**	
	**Sample ID**	**# Intervals**	**# bps**	**# Intervals**	**# bps**
**Basenji 1**[a]	RKW 13764	1,402	53,445,975	784	44,666,307
**Basenji 2**	1756	1,409	53,142,107	761	43,921,743
**Croatian wolf**	RKW 3919	1,413	54,845,287	817	46,428,462
**Dingo**	RKW13760	1,386	53,042,846	776	44,439,961
**Golden Jackal**	RKW 1332	1,379	52,769,259	774	44,155,006
**Israeli wolf**	RKW13759	1,368	55,014,821	796	46,875,363

[a] Basenji used for all other analyses throughout the paper.

genome was reported as being duplicated with the same method. In that study, Nicholas et al. [4] used Sanger capillary reads from the same dog that was also used to build the canFam2 reference genome. This difference is likely due to different treatment of repeat sequences in Sanger vs. next-generation sequencing datasets.

Table S6.1.2. Segmental duplications detected as regions with at least 5 consecutive non-overlapping windows with a copy number higher than 2.5 from SOLiD reads. The overlapping bps with the predicted SDs from the Illumina dataset are also shown.

	**>10kb**			**>20kb**		
	**# Intervals**	**# bps**	**Intersection with Illumina data**	**# Intervals**	**# bps**	**Intersection with Illumina data**
**Basenji**	1453	54,580,095	45,605,293[a]	810	45,406,265	38,384,115[a]
**Croatian wolf**	1276	51,410,183	49,612,023	753	43,912,580	42,457,271
**Dingo**	1422	54,604,031	48,165,059	808	41,228,862	41,228,862
**Golden Jackal**	1234	49,330,533	47,072,480	740	42,241,860	40,316,493
**Israeli wolf**	1289	52,032,514	49,980,206	743	44,192,703	42,796,663

[a] For Basenji, we took the intersection of the two Illumina lanes from the two individuals.

To help reduce potential false negatives of the conservative approach outlined above, we applied an alternative strategy for SD calling that is highly similar to the one used for Sanger reads. We identified SDs as regions having a higher read depth than the mean coverage plus 4 standard deviation in at least 6 out of 7 overlapping windows of 5 kb of unmasked and non-gapped sequence. We predicted between 7,456 and 8,202 regions as SDs longer than 10 kb representing between 4.93% to 5.63% of the reference assembly (Table S6.3). We also added the

WSSD regions from the reference genome to this dataset [4] and the final list was used to exclude paralogous regions in the SNP calling (Figure S6.1.1). We note that the conservative strategy may have higher rate of false negatives, while this alternative method potentially has a higher false positive rate.

Table S6.1.3. Segmental duplications detected with at least 6 out of 7 5kb overlapping windows showing a read depth higher than the 4 standard deviations above the average, detected using Illumina reads.

		**>10kb**		**>20kb**	
**Sample**	**Sample ID**	**# Intervals**	**# bps**	**# Intervals**	**# bps**
**Basenji 1[a]**	RKW 13764	7,597	126,674,600	5,506	99,205,302
**Basenji 2**	1756	8,202	142,461,157	5,966	112,954,332
**Croatian wolf**	RKW 3919	7,632	128,344,367	5,551	100,981,951
**Dingo**	RKW13760	8,043	140,574,072	5,798	110,892,809
**Golden Jackal**	RKW 1332	7,456	124,758,677	5,397	97,652,991
**Israeli wolf**	RKW13759	7,724	129,350,771	5,545	100,780,739

[a] Basenji used for all other analyses throughout the paper.

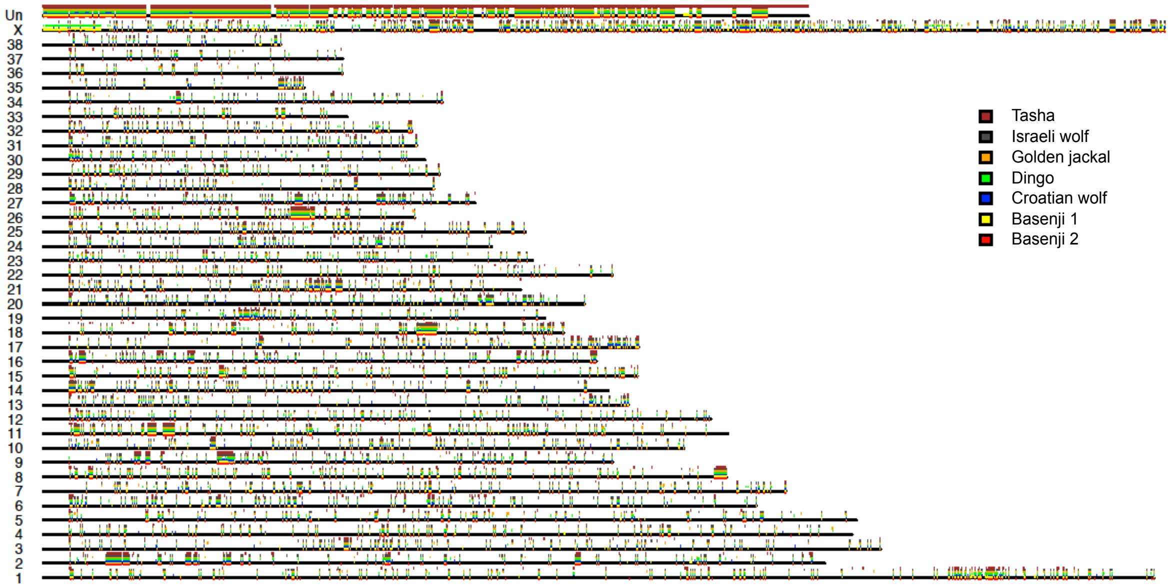

Figure S6.1.1. SD distribution on the dog genome (CanFam2). Each horizontal line refers to a chromosome in the dog assembly. Tasha refers to the duplications detected in the reference [4].

S6.2 Copy Number Variation at the Amylase (*AMY2B*) Locus

The amylase activity, which cleaves the starch into maltose, has been affected by gene duplication events in the recent history of both humans and dogs [5,6]. In dogs the *AMY2B* gene, that encodes the alpha-2B-amylase enzyme, is present with a high variety of copy number states while in wolves has always being found as single copy. To date, this gene model in dogs has been predicted by Ensembl (http://www.ensembl.org/) and is localized in three regions on the Unknown chromosome of CanFam2. Moreover, there is a partial or unresolved copy of this gene on chromosome 6 detected using BLAT (see Table S6.2.1, Figure S6.2.1).

Table S6.2.1. Location of *AMY2B* in CanFam2.

Chromosome	Start	End	Length	Strand	Gene ID*
ChrUn	4,462,782	74,468,964	6,183	+	ENSCAFG00000032684
ChrUn	62,479,904	62,496,097	16,194	+	ENSCAFG00000030588
ChrUn	6,712,667	46,719,782	7,116	+	ENSCAFG00000031239
Chr6	50,008,123	50,014,414	6,292	-	

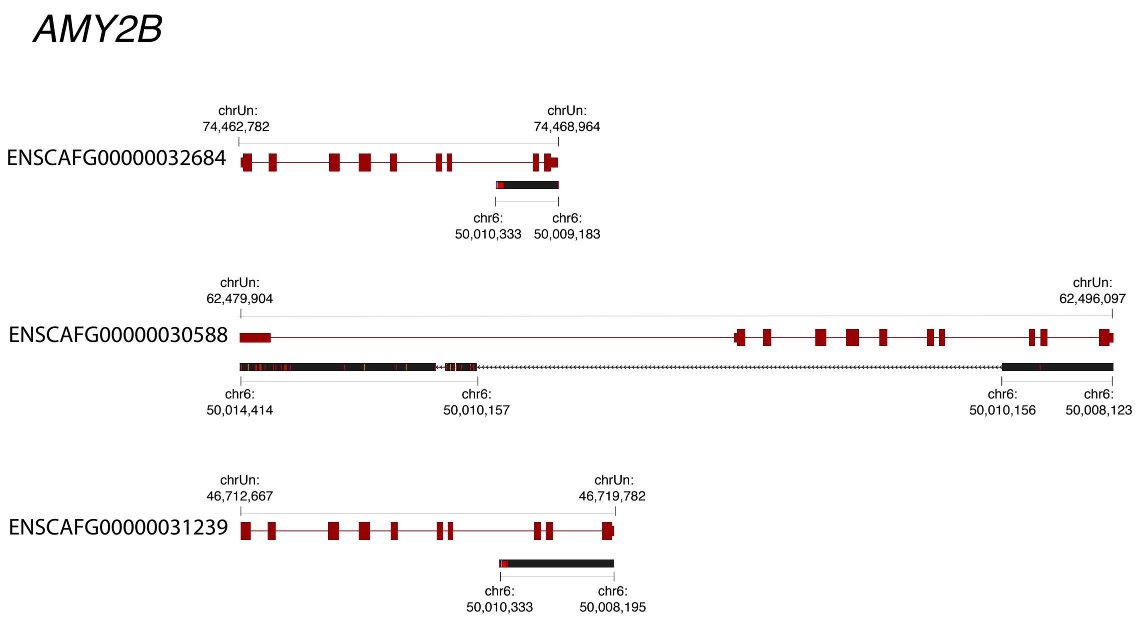

Figure S6.2.1 Region of chromosome 6 mapped to *AMY2B* genes in CanFam2. A duplication with a deleted fragment or a bad representation of this region in the assembly might explain the data.

To determine the duplication status of the amylase gene in our samples we calculated the read depth on contiguous 1kb windows of non-repetitive sequence. The number of copies of any window is estimated by dividing between the average coverage in the genome. In such a way, the expected value for diploid single-copy regions would be around 2. For *AMY2B* this is the case in all our samples except for Basenji, for which the copy number is higher than 2. The average of the windows containing the gene

(ENSCAFG00000032684 and ENSCAFG00000031239) in Basenji is around 9 copies. In the case of the predicted gene ENSCAFG00000030588, the first exon, which is non-coding, might be single-copy, as can be inferred from its copy number. This fragment is also shown in the region of chromosome 6 where the gene is partially represented (Figures S6.2.2 and S6.2.3, Table S6.2.2).

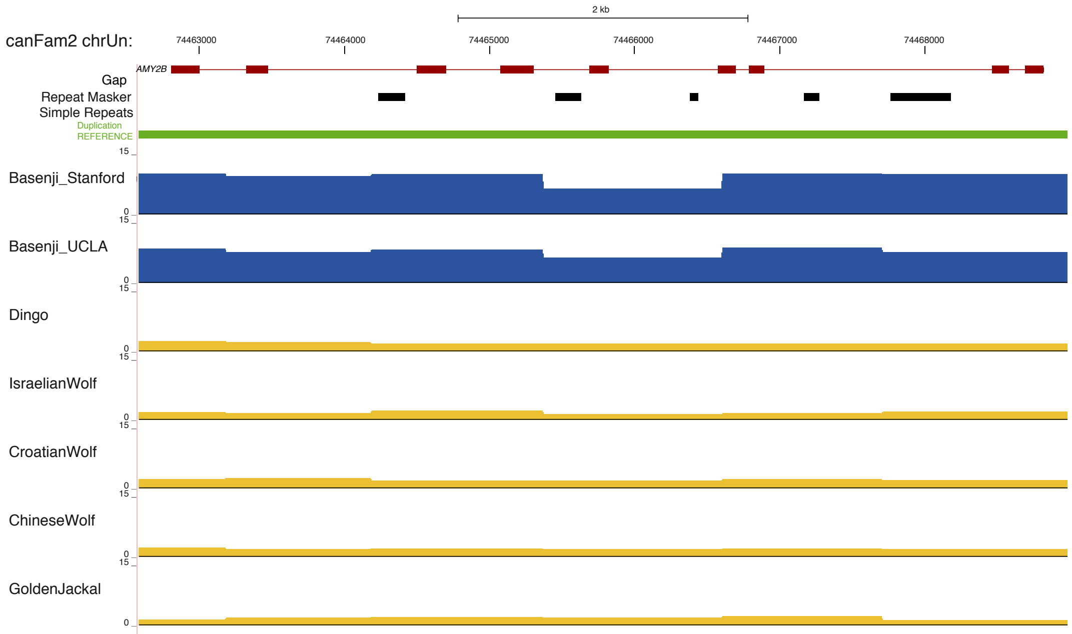

Figure S6.2.2 Average copy number on 1kb windows in the region of ENSCAFG00000032684 (in red) in chrUn. In yellow, expected copy number of single copy regions. In blue, higher copy numbers. In green we represented a duplicated region according to the dog reference genome assembly (Tasha) [4]. Repeats and gaps of the region are also shown.

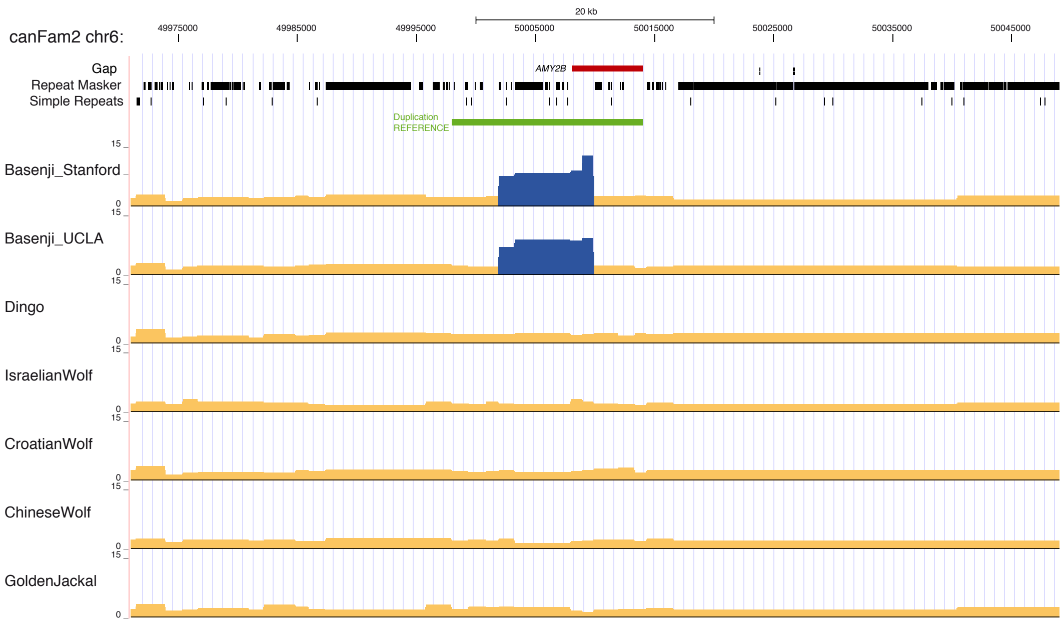

Figure S6.2.3. Average copy number on 1kb windows in the region of chromosome 6 where *AMY2B* is partially represented. The putative location of the gene was determined by aligning into this region the sequence of the predicted genes, and the maximum region (that corresponds to ENSCAFG00000030588) is shown here. In yellow, expected copy number of single copy regions. In blue, regions with higher copy number.

Table S6.2.2 Copy number on windows containing *AMY2B* for Basenji samples. Windows showing evidence for gene duplications are highlighted in green.

	Windows			Copy Number	
	**Chr**	**Start**	**End**	**Basenji_ Stanford**	**Basenji_ UCLA**
ENSCAFG00000032684 (ChrUn:74,462,782-74,468,964)	chrUn	74,462,186	74,463,186	10.17	8.51
	chrUn	74,463,186	74,464,186	9.48	7.63
	chrUn	74,464,186	74,465,370	9.81	8.29
	chrUn	74,465,370	74,466,604	6.41	6.19
	chrUn	74,466,604	74,467,705	10.02	8.55
	chrUn	74,467,705	74,469,120	9.90	7.50
ENSCAFG00000030588 (ChrUn:62,479,904-62,496,097)	chrUn	62,473,212	62,480,658	6.42	4.64
	chrUn	62,480,658	62,481,658	2.52	2.21
	chrUn	62,481,658	62,482,870	1.86	1.71
	chrUn	62,482,870	62,485,453	1.92	2.02
	chrUn	62,485,453	62,488,822	6.54	5.58
	chrUn	62,488,822	62,489,822	9.70	8.51
	chrUn	62,489,822	62,490,822	9.44	6.89

	chrUn	62,490,822	62,491,999	9.25	8.06
	chrUn	62,491,999	62,493,056	7.59	6.68
	chrUn	62,493,056	62,494,572	9.45	8.72
	chrUn	62,494,572	62,495,572	12.42	9.26
	chrUn	62,495,572	62,507,548	6.37	6.47
ENSCAFG00000031239 (ChrUn:46,712,667-46,719,782)	chrUn	46,710,448	46,712,776	9.52	7.74
	chrUn	46,712,776	46,713,776	9.94	8.50
	chrUn	46,713,776	46,714,961	9.60	7.55
	chrUn	46,714,961	46,716,138	7.23	6.93
	chrUn	46,716,138	46,717,296	8.88	7.64
	chrUn	46,717,296	46,718,711	10.85	8.81
	chrUn	46,718,711	46,719,711	10.81	8.86
Chr6:50008123-50014,414	chr6	50,007,907	50,008,907	8.78	8.39
	chr6	50,008,907	50,009,907	12.51	9.13
	chr6	50,009,907	50,011,943	2.42	2.10
	chr6	50,011,943	50,013,300	2.36	2.25
	chr6	50,013,300	50,014,300	2.51	1.65
	chr6	50,014,300	50,016,584	2.48	1.95

S6.3 Validation of copy number of *AMY2B* by real-time quantitative PCR (qPCR)

We explore the variation in *AMY2B* copies using qPCR across additional breed dogs (n=52), dingoes (n=6) and a globally distributed panel of wolves (n=40) (Table S13). This new data improve specially the variability presented in wolves, with the analysis of samples from 9 wolf populations, 5 of them not previously explored. Also this data allow us to validate the copy number of *AMY2B* estimated based on whole genome sequencing (Table S6.3.1). Estimation of copy number was performed using the Multiplex TaqMan assays previously described by Axelsson et al. [5].The duplex reaction contained a reference assay designed to amplify *C7orf28B* that is known to exist in two copies in a canid genome (900 nM of forward and reverse primers, 250 nM VIC and TAMRA labeled probe, Applied Biosystems), and the *AMY2B* as a target gene (300 nM of forward and reverse primers, 250 nM FAM labeled MGB probe, Applied Biosystems) in genomic DNA. For each sample we performed three replicates.

Table S6.3.1. Amylase copy number in 10 dogs estimated by qPCR and genome sequencing.

Sample	Copy Number from qPCR	Copy Number from Genome Sequencing
Beagle	6	7
Bulldog	14	15
Chihuahua	10	10
Flat-coated retriever	12	10
Great dane	16	16
Mastiff	8	12
Pekingese	14	14
Saluki	23	29
Scottish terrier	8	9
Siberian husky	3	3

References

1. Bailey JA, Gu ZP, Clark RA, Reinert K, Samonte RV, et al. (2002) Recent segmental duplications in the human genome. Science 297: 1003-1007.
2. Alkan C, Kidd JM, Marques-Bonet T, Aksay G, Antonacci F, et al. (2009) Personalized copy number and segmental duplication maps using next-generation sequencing. Nat Genet 41: 1061-1067.
3. Hormozdiari F, Hach F, Sahinalp SC, Eichler EE, Alkan C (2011) Sensitive and fast mapping of di-base encoded reads. Bioinformatics 27: 1915-1921.
4. Nicholas TJ, Cheng Z, Ventura M, Mealey K, Eichler EE, et al. (2009) The genomic architecture of segmental duplications and associated copy number variants in dogs. Genome Res 19: 491-499.
5. Axelsson E, Ratnakumar A, Arendt M-J, Maqbool K, Webster MT, et al. (2013) The genomic signature of dog domestication reveals adaptation to a starch-rich diet. Nature 495:360-364.
6. Meisler MH, Ting CN (1993) The Remarkable Evolutionary History of the Human Amylase Genes. Crit Rev Oral Biol Med 4: 503-509.
