# Supplementary material for: Genome Sequencing Highlights the Dynamic Early History of Dogs
Source: PLoS Genet. 2014 Jan 16;10(1):e1004016. doi: 10.1371/journal.pgen.1004016 (PMC3894170; doi:10.1371/journal.pgen.1004016)
Supplement: Text S7 — Methods for annotation of genes and conserved non-coding regions. (PDF) [file pgen.1004016.s026.pdf]

S7 Gene Annotations

***Pedro Silva*[1]*, Marco Galaverni*[2]*, Rena M. Schweizer*[3]*, Adam H. Freedman*[3]*, Robert K. Wayne*[3]*, John Novembre*[3]**

[1] ***University of Porto***
CIBIO-UP - Research Center in Biodiversity and Genetic Resources
Porto, Portugal

[2] ***Istituto Superiore per la Protezione e la Ricerca Ambientale***
Laboratorio di Genetica
Ozzano dell'Emilia, Italy

[3] ***University of California, Los Angeles***
Department of Ecology and Evolutionary Biology
Los Angeles, California, United States of America

In order to construct a set of neutral regions for use in demographic analyses, we generated a set of annotations for regions likely evolving in a non-neutral fashion. These consisted of genic and other regions showing a high degree of conservation or that might otherwise play a functional role, and were identified as described below.

S7.1 Identification of Genes

In order to build a comprehensive set of annotated genes in the domestic dog, we compiled the available information from three different sources: the refGene file from the UCSC genome browser database [1] (downloaded from ftp://hgdownload.cse.ucsc.edu/goldenPath/canFam2/database/ on Aug 15, 2011); Ensembl [2] (all protein coding genes from Ensembl Release 63 downloaded Jul 26, 2011 via the BioMart MartView tool: http://www.ensembl.org/biomart/martview), and SeqGene files from the NCBI database (downloaded Mar 02, 2011 from ftp://ftp.ncbi.nih.gov/genomes/Canis_familiaris/mapview/). All downloaded information pertains to the May 2005 assembly of the dog genome (canFam2) [3]. The retrieved information included annotated gene names and symbols, genomic coordinates of coding exons and untranslated regions (UTRs) (including annotations for alternatively spliced transcripts) of both confirmed and predicted genes.

UCSC refGene contained 1,168 entries (transcripts) corresponding to 1,131 unique gene symbols, data from Ensembl yielded 30,914 transcripts from 24,660 different genes, and NCBI's seq_gene contained 33,636 transcripts from 19,758 genes. As currently available gene annotations are all provided with reference to genomic coordinates in canFam2, as with the Canine BeadChip genotypes (see Text S4.1), we used the command-line version of the UCSC *liftover* tool (http://genome.ucsc.edu/cgi-bin/hgLiftOver) to convert annotations to canFam 3.0 (see SI Text S3.1.1 for details concerning the reference) coordinates. 36 transcripts (27 genes) from RefGene, 736 (600 genes) from Ensembl and 1,368 (977 genes) from NCBI failed to be converted.

Many of the entries in NCBI's SeqGene file had provisional LOC codes as the only available information, so an effort was made to obtain gene symbols and descriptions for those loci. For this, we used NCBI's BatchEntrez tool (http://www.ncbi.nlm.nih.gov/sites/batchentrez) to query the 'Gene' database. Many of the records were already discontinued, so those entries were eliminated. The final SeqGene annotation from NCBI comprised 32,200 transcripts from 18,601 genes.

From the initial concatenated set of annotations from the three sources, we created a reduced set in which we merged entries that appeared to be duplicates. In order for any two annotated genes to be considered duplicates, they had to have overlapping coordinates and be transcribed on the same strand. In addition, they had to either possess similar symbols, similar gene descriptions, a symbol of one matching the transcript IDs of the other, or share ≥90% of their exonic sequence. While the last of these criteria is somewhat arbitrary, we chose it based upon the observation that only a very small percentage of annotations known to be unique displayed such a high degree of exonic overlap. The majority of annotation merges depending upon this criterion were sparsely annotated, typically falling into the "unknown gene" category. As an understanding of the functional role for many predicted genes in the dog is incomplete, we chose to retain such annotations. The final gene annotation set consisted of 28,805 genic regions with 63,510 associated transcripts.

We distinguished what appeared to be functional transcripts ('CDS OK'), containing properly positioned start and stop codons and a transcript length that is a multiple of 3 bp, from those that were not. Approximately 19% of transcripts from UCSC RefGene, 23% from Ensembl, and 89% from NCBI SeqGene satisfied these conditions. From these, we retained the longest transcript from each unique gene annotation, and used these to build our final transcript annotation set; in those cases where a gene contained more than one transcript with the same length, one was chosen randomly. This final transcript set ('CDS-OK longest transcripts') consisted of 19,910 transcripts. Figure S7.1 shows the provenance of the transcripts that constitute this final set.

The transcripts that did not pass the CDS filters in the boxer genome, probably due for the most part to improper annotation, were still retained and grouped in an additional annotation dataset ('CDS-fail transcripts' set of 23,079 transcripts).

S7.2 Identification of conserved non-coding regions

Recent research has indicated conserved non-coding elements can play an important role in modulating the regulation of gene expression [4,5]. In particular, such regions conserved across vertebrates, but showing acceleration on the human lineage (HARs), have been implicated in the rapid acquisition of traits unique to humans [4]. To identify conserved elements in dogs, we first identified conserved genomic regions in a set of mammals not including the dog, through examination of a multi-genome alignment of 11 species of the mammalian Euarchontoglires clade, using mouse as reference: mouse, rat, guinea pig, rabbit, human, chimpanzee, orangutan, rhesus macaque, marmoset, bushbaby and tree shrew. The Euarchontoglires (Supraprimates) represent a sister clade to the Laurasiatheria clade that includes carnivores and allows us to identify mammalian conserved regions of the genome without the influence of dog/canid specific changes.

We identified conserved non-coding elements (CNEs) using phastCons scores [6]

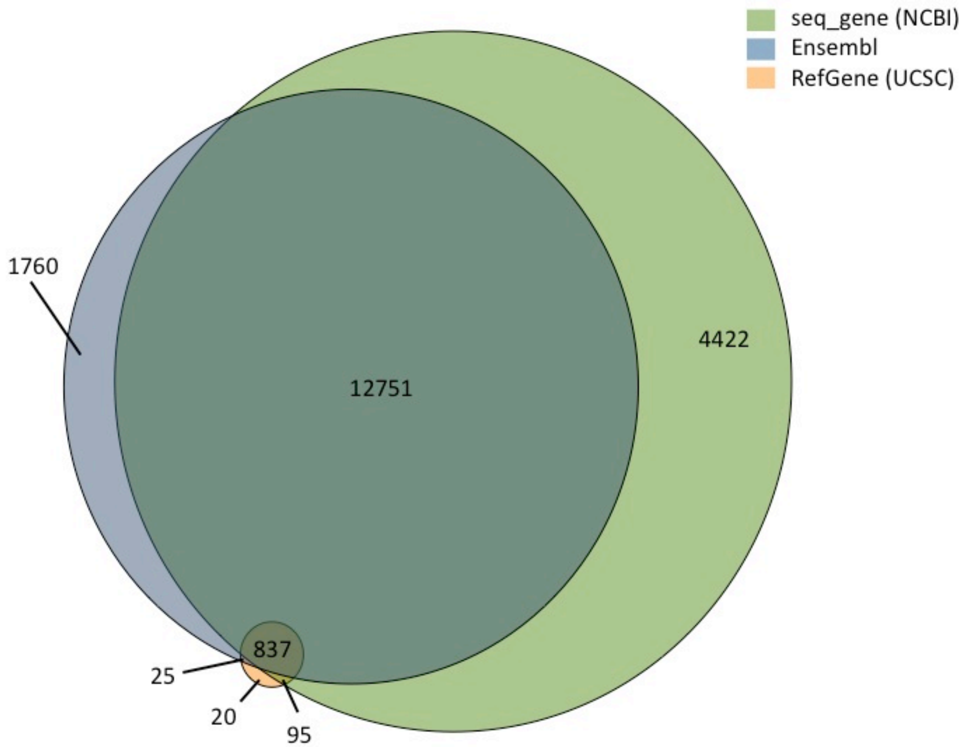

Figure S7.1. Origin of the transcripts in the final CDS OK transcript set. Numbers denote the amount of transcripts found in each database; intersections represent merged gene entries.

provided for the Euarchontoglires clade available on UCSC for the mouse genome (http://hgdownload.cse.ucsc.edu/goldenPath/mm9/phastCons30way/euarchontoglires/). Conserved regions of the mouse genome were defined as stretches of consecutive bases with phastCons scores > 0.7 longer than 50 bp. The 50 bp threshold was chosen because this approximates the lower size limit of miRNA genes (www.mirbase.org), and such genes have been previously discovered within HARs [4]. The genomic locations of these regions were then converted to the CanFam 3.0 assembly of the dog genome using the command-line version of the UCSC *liftover* tool (http://genome.ucsc.edu/cgi-bin/hgLiftOver). CNEs were then defined by the intersection of these conserved regions with the non-coding portion of CanFam 3.0.

S7.3 Regulatory Region Variation (UTR, Promoters, Dog-wolf Differences in Dog-Conserved Binding Motifs)

Given the possible effects of gene regulation on phenotypic traits that differentiate dogs from their wild ancestors, we classified regulatory regions flanking coding sequences including the 5' and 3' untranslated regions (UTRs) and promoter regions. These regions were defined based on our CDS OK annotation set. UTRs were defined as the regions between the annotated transcription start/end site and the first base of the initiation codon/last base of the stop codon. 5'UTR were defined for 8,427 (~42%) and 3'UTR for 11085 (~56%) of CDS OK transcripts. 6,581 (~33%) of the transcripts had both types of UTRs.

Promoter regions were considered as the 1Kb regions upstream of the transcription start site, considering strand orientation. Putative transcription factor binding sites (TFBS) were searched within the promoter regions using the profiles in the JASPAR PHYLOFACTS database

(http://jaspar.cgb.ki.se/) since this database contains count matrices of conserved motifs in human, mouse, rat and dog, originally identified by [7].The motifs were converted to probability weight matrices and used with the motif finding program FIMO [8], part of the MEME package (http://meme.sdsc.edu) to find matching occurrences in the promoter regions of the dog genome. A total number of 866,242 putative binding sites were identified.

References

1. Fujita PA, Rhead B, Zweig AS, Hinrichs AS, Karolchik D, et al. (2011) The UCSC Genome Browser database: update 2011. Nucleic Acids Rese 39: D876-D882.
2. Birney E, Andrews TD, Bevan P, Caccamo M, Chen Y, et al. (2004) An overview of ensembl. Genome Res 14: 925-928.
3. Lindblad-Toh K, Wade CM, Mikkelsen TS, Karlsson EK, Jaffe DB, et al. (2005) Genome sequence, comparative analysis and haplotype structure of the domestic dog. Nature 438: 803-819.
4. Pollard KS, Salama SR, Lambert N, Lambot MA, Coppens S, et al. (2006) An RNA gene expressed during cortical development evolved rapidly in humans. Nature 443: 167-172.
5. Lowe CB, Kellis M, Siepel A, Raney BJ, Clamp M, et al. (2011) Three Periods of Regulatory Innovation During Vertebrate Evolution. Science 333: 1019-1024.
6. Siepel A, Bejerano G, Pedersen JS, Hinrichs AS, Hou MM, et al. (2005) Evolutionarily conserved elements in vertebrate, insect, worm, and yeast genomes. Genome Res 15: 1034-1050.
7. Xie XH, Lu J, Kulbokas EJ, Golub TR, Mootha V, et al. (2005) Systematic discovery of regulatory motifs in human promoters and 3 ' UTRs by comparison of several mammals. Nature 434: 338-345.
8. Grant CE, Bailey TL, Noble WS (2011) FIMO: scanning for occurrences of a given motif. Bioinformatics 27: 1017-1018.
