# Supplementary material for: Genome Sequencing Highlights the Dynamic Early History of Dogs
Source: PLoS Genet. 2014 Jan 16;10(1):e1004016. doi: 10.1371/journal.pgen.1004016 (PMC3894170; doi:10.1371/journal.pgen.1004016)
Supplement: Text S8 — Methods and supporting results for demographic analyses using sequence divergence, ABBA/BABA tests and PSMC. (PDF) [file pgen.1004016.s027.pdf]

S8 Demographic Analyses Using Sequence Divergence, ABBA/BABA Tests and PSMC

Diego Ortega Del Vecchyo[1], Zhenxin Fan[2], Adam H. Freedman[1], Rena M. Schweizer[1], Pedro Silva[3], Robert K. Wayne[1], John Novembre[1]

[1]University of California, Los Angeles
Department of Ecology and Evolutionary Biology
Los Angeles, California, United States of America

[2]Sichuan University
Sichuan Key Laboratory of Conservation Biology on Endangered Wildlife, College of Life Sciences
Chengdu, People's Republic of China

[3]University of Porto
CIBIO-UP - Research Center in Biodiversity and Genetic Resources
Porto, Portugal

S8.1 Distance Matrices and Phylogenetic Tree Reconstruction

S.8.1.1 Distance Metrics

We computed a matrix with the pairwise genetic distances between each of the 6 canid genomes and the reference Boxer sequence using the genetic distance metric from Gronau et al. [1]:

$$d(X,Y) = \frac{1}{L}\sum_{i=1}^{L}[1 - \frac{1}{2}\ \max\ (\delta_{a_i c_i} + \delta_{b_i d_i}, \delta_{a_i d_i} + \delta_{b_i c_i})] \tag{E8.1}$$

where X and Y represent the two genomes being compared, L is the total number of sites utilized in the analysis, a_i and b_i are the two allele copies carried by individual X, c_i and d_i are the two allele copies carried by individual Y and δ_{jk} represents the Kronecker delta function (i.e. in this case equals one if allele j is identical to allele k and 0 otherwise).This measure represents a conservative estimate of the expected number of differences per site between individual chromosomes drawn (Gronau et al, 2011, S3.2).

We also computed the average number of nucleotide differences per site among a pair of randomly drawn alleles from each individual, using the following equation:

$$d(X,Y) = \frac{1}{L}\sum_{i=1}^{L}[1 - \frac{1}{4}(\delta_{a_i c_i} + \delta_{b_i d_i} + \delta_{a_i d_i} + \delta_{b_i c_i})] \tag{E8.2}$$

In order to be included in the analysis, sites had to pass the GF2 and SF filters and had no missing genotypes for all of the six samples.

S8.1.2 Results on Genome-wide Pairwise Distances

We took all of the sites across the genome that passed the quality filters defined above to compute a matrix of pairwise distances between all canid genomes using E8.1 and E8.2 (Tables S8.1.1 and S8.1.2, respectively). The distances of all taxa to the golden jackal are very similar

(approximately 0.0021) while the distances between dogs and wolves were about a half of that (0.0011). We used the matrix of pairwise distances generated by E8.1 and E8.2 to generate phylogenetic trees using the neighbor joining method as implemented on the program *neighbor* of the phylogenetic package *PHYLIP* [2].

In the neighbor-joining tree generated by using E8.1 (Figure S8.1.1A), all dogs were clustered into a single clade. Wolves also comprised a single clade, separated from other species by a branch of relatively short length. The Dingo was recovered as the outgroup to a clade comprised of Basenji and Boxer. Similarly, the Chinese wolf was inferred as the outgroup to the clade formed by the Israeli and Croatian wolves. Thus, the phylogenetic tree supports the hypothesis that dogs and wolves are reciprocally monophyletic taxa.

The tree created using E8.2 (Figure S8.1.1B) differs from the previous tree in the position of the Chinese Wolf lineage. The Chinese Wolf appears as an outgroup to the clade comprised of the remaining dogs and wolves. However, the bootstrap support is low for both the branch that joins that lineage to the whole wolf-dog clade (54.2%) and the branch ancestral to the clade comprised of the Croatian and Israeli wolves 53.7%).

Table S8.1.1. Genome-wide pairwise sequence divergence, estimated using E8.1 using all the genomic sites that passed the genomic quality filters outlined in S.8.1.1.

	Boxer	Basenji	Dingo	Israeli wolf	Croatian wolf	Chinese wolf	Golden jackal
Boxer							
Basenji	0.00087						
Dingo	0.00094	0.00097					
Israeli wolf	0.00111	0.00105	0.00111				
Croatian wolf	0.00113	0.00110	0.00112	0.00101			
Chinese wolf	0.00114	0.00111	0.00111	0.00106	0.00105		
Golden jackal	0.00211	0.00211	0.00212	0.00209	0.00209	0.00210	

Table S8.1.2. Genome-wide pairwise sequence divergence, estimated using E8.2 using all the genomic sites that passed the genomic quality filters outlined in S.8.1.1.

	Boxer	Basenji	Dingo	Israeli wolf	Croatian wolf	Chinese wolf	Golden jackal
Boxer							
Basenji	0.00087						
Dingo	0.00094	0.00100					
Israeli wolf	0.00111	0.00112	0.00116				
Croatian wolf	0.00113	0.00117	0.00116	0.00115			
Chinese wolf	0.00114	0.00117	0.00115	0.00118	0.00115		
Golden jackal	0.00211	0.00214	0.00214	0.00214	0.00214	0.00214	

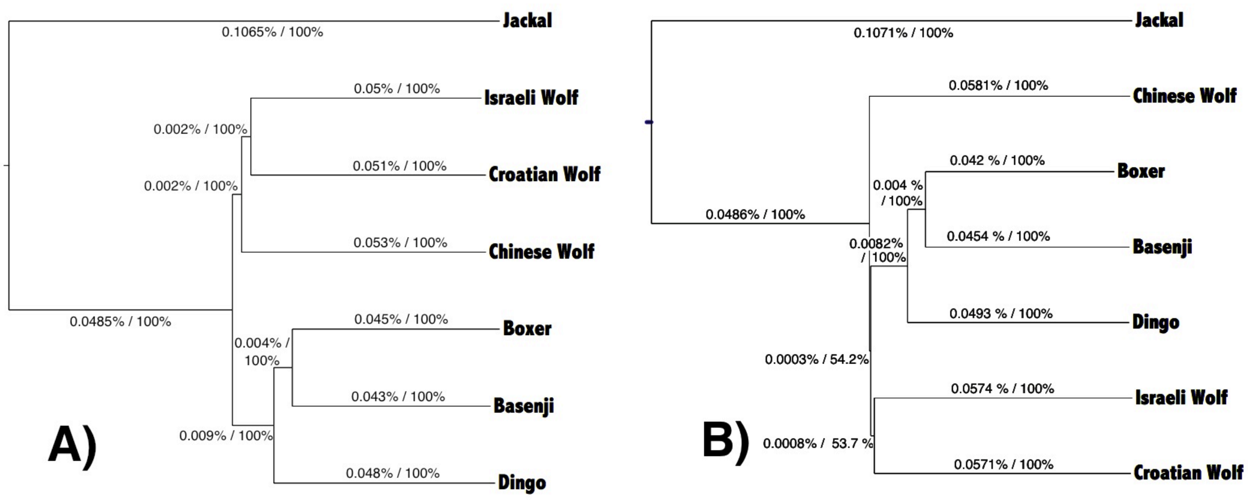

Figure S8.1.1. Neighbor-joining tree of canid samples plus the Boxer reference (CanFam3.0) for all positions passing the GF2 and SF filters and for which there was no missing data for any sample. The distance metrics used were E8.1 and E8.2 for panel A) and B), respectively. For each branch, we report the genetic distance (left side of the slash) and the bootstrap support (right side of the slash). Bootstrap replicates were generated by dividing the genome of each species into windows of 500 kb based on the genomic coordinates of the Boxer reference, and then resampling with replacement from those windows until the bootstrapped genomes for each species contain an equal or greater number of sites called as the true genomes.

S8.2 Population Size Change From Single Genome Sequences

S8.2.1 PSMC: General Approach

We used the methods developed by Li and Durbin [3] to infer the trajectory of population sizes across time for the six canid genome sequences. Briefly, the method uses the distribution of heterozygote sites across the genome and a pairwise sequentially Markovian coalescent (PSMC) model that defines a Hidden Markov Model, where the parameters are the mutation rate, recombination rate and the effective population sizes through time. The parameters are inferred through an Expectation-Maximization algorithm.

The genotypes for each diploid genome sample that passed the GF2 and SF filters were transformed into a sequence of '0', '1' and '.', with one character for each 100bp, and where a '1' was assigned if there were heterozygous sites in the window, 0 if there were none, and a '.' was given if more than 90 positions were missing in the 100 bp window. Passing this data into the PSMC software, we ran 20 iterations of the Expectation-Maximization algorithm [3]. The EM algorithm was run using an upper bound on the time to the most recent common ancestor equal to 10 in a $2N_0$ scale and an initial θ/ρ set to the default value of 5. Following [3], the N_e was inferred across 64 different intervals for each dog genome, where the interval boundaries were set equal to:

$$t_i = 0.1\ \exp\ [\frac{1}{n}\ \log\ (i + 100)] - 0.1$$

on a $2N_0$ scale ,where i takes values from 0 to 64. In a preliminary run we found that the number of recombination events inferred in the most recent time intervals by PSMC falls below 10. In such situations, the authors of PSMC recommend refraining from inferring a population size during such time intervals. Thus, we merged the first 6 intervals such that only a single N_e is

inferred across them while the next 58 intervals were allowed to have interval-specific N_e values (in the Chinese wolf, the number of recombination events was higher and thus we continued to use all 64 intervals).

To translate from time units of generations to calendar years, we assume a generational time of 3 years for the wolves and the golden jackal. For the Dingo and the basenji, we used a generational time of 2 years from the present until the N_e interval that reached 10,000 years ago and for all N_e intervals further into the past, we used a generational time of 3 years. We found this scaling improved the concordance of the trajectories during the ancestral period where we expect them to be identical across lineages and is motivated by the known shorter generation time in domestic dogs. Following Lindblad-Toh *et al.* [4], the mutation rate assumed was 1.0×10^{-8} per generation.

The full results including the golden jackal are shown here (Figure S8.2.1). The golden jackal shows an apparent large increase in effective populations size around 80,000 years ago. We address interpretations of this signal in more detail in the results of our validation study (see below).

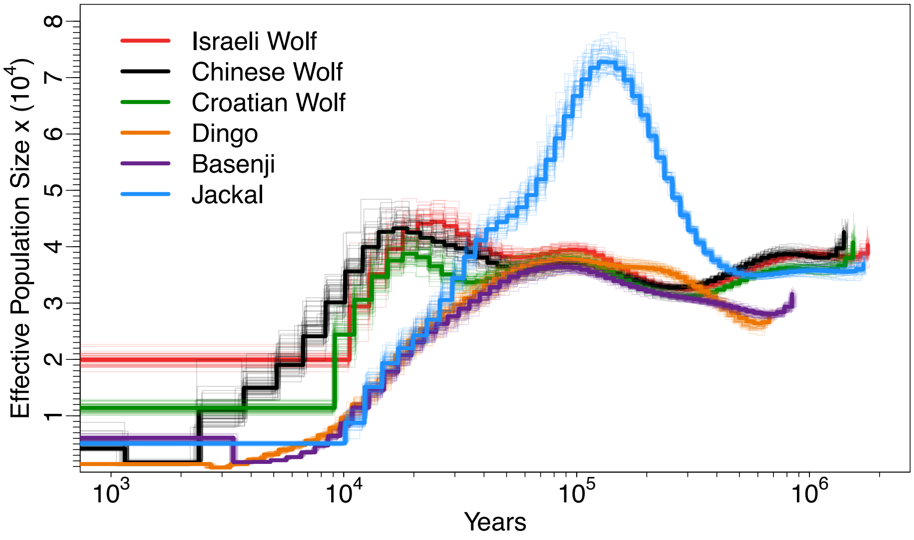

Figure S8.2.1. N_e trajectories of 6 canid lineages reconstructed using the PSMC method of Li and Durbin [3]. Dark and light lines indicate whole genome based estimates and bootstrap estimates, respectively.

S8.2.2 Validation

We assessed the confidence in our PSMC findings in three ways. First, to assess the certainty in the inferred N_e trajectories, we ran the PSMC method using the same settings for the initial estimations, assessing the variance in those estimates from 100 bootstrap replicates for each genome. To sample a bootstrap replicate, we divided the genome into segments of 5Mb, sampled with replacement from those segments until we obtained a sequence with approximately the same length as the original genome as defined by using the "-b" option in the PSMC software, and re-ran the EM-based N_e estimation procedure. This analysis revealed a low variability among the N_e traces, comparable to what has been recovered in the analysis of human genome sequences (Figure S8.2.1) [3].

Second, we tested the sensitivity of the methods to long runs of homozygosity (RoH), as the Chinese wolf sample evidenced several runs (see Text S5.6). To test if long runs of homozygosity could bias the inference of Ne trajectories, we identified runs of homozygosity

with the program PLINK [5] (see Text S5.6, Figure S5.6.1). As can be seen in Figure S8.2.2, the estimated trajectories are not affected by the removal of the RoH regions. This implies that the degree of inbreeding in the Chinese wolf is not large enough to bias the inference of ancestral demographic events estimated by the PSMC method.

Third, to investigate the sensitivity of PSMC to our choice of minimum acceptable genotype quality (GQ≥20), we ran the PSMC analysis including the genotypes that passed the GF2 and SF filters, but relaxing the GQ component of SF such that we included sites with GQ≥10 (as a contrast, Figure S8.2.1 and Figure 3B use the genotypes that passed the GF2 and SF1 filters and had a GQ >= 20). Using this more liberal GQ threshold, values of N_e are lower by approximately 1,000 along the trajectory of all canids (Figure S8.2.3), however the N_e trajectories remain largely concordant. The effect is particularly strong in the golden jackal between 50,000 – 300,000 years ago, where using a lower GQ threshold reduces the estimates of N_e by 2,000. The difference between the dog and wolf N_e at earlier times (5,000-70,000 years) is more noticeable when using a higher GQ threshold. The reductions in N_e across the PSMC traces are consistent with expectations with respect to how confidence in genotype quality scales differently for homozygous versus heterozygous genotype calls. Homozygous sites can be called confidently with less data that is of lower quality. Conversely, heterozygous calls will require more and higher quality data, such that genotype qualities at those sites will be higher. As a result, lowering the GQ threshold leads to the inclusion of disproportionately more homozygous genotypes than low quality heterozygous ones, reducing the observed heterozygosity within defined intervals, and as a result, the inferred N_e. Overall, although changes in GQ filtering does influence the estimates of the N_e trajectories, the magnitude of the changes are not large, and more importantly, the major patterns in the inferred trajectories are preserved.

Fourth, we simulated genome sequences arising from the demographic history inferred from the model analyzed by *G-PhoCS* which assumes that wolves and dogs are reciprocally monophyletic taxa (see Table S9.2 and Figure S9.1) to determine if we could accurately reconstruct changes in N_e conditional on such a history. Specifically, for each species we simulated one hundred regions of 30Mb apiece using the program MaCS [6]. We conducted these simulations under three different scenarios, varying the levels of gene flow between lineages. We used parameter values from the main results obtained with *G-PhoCS* (see Table S9.2). The scenarios tested used:

1) The full model inferred from *G-PhoCS* (Command Line 1, see command-line parameter listings below).
2) Our model inferred with *G-PhoCS* but with no gene flow between any species at any time (Command Line 2).
3) The model inferred by *G-PhoCS* but with only one form of gene flow, from golden jackal to the ancestor of dogs and wolves (Command Line 3).
4) The model inferred by *G-PhoCS* but with only one form of gene flow, from the ancestor of dogs and wolves to the golden jackal (Command Line 4).
5) The model inferred by *G-PhoCS* but only with gene flow from the Israeli wolf to the golden jackal (Command Line 5).

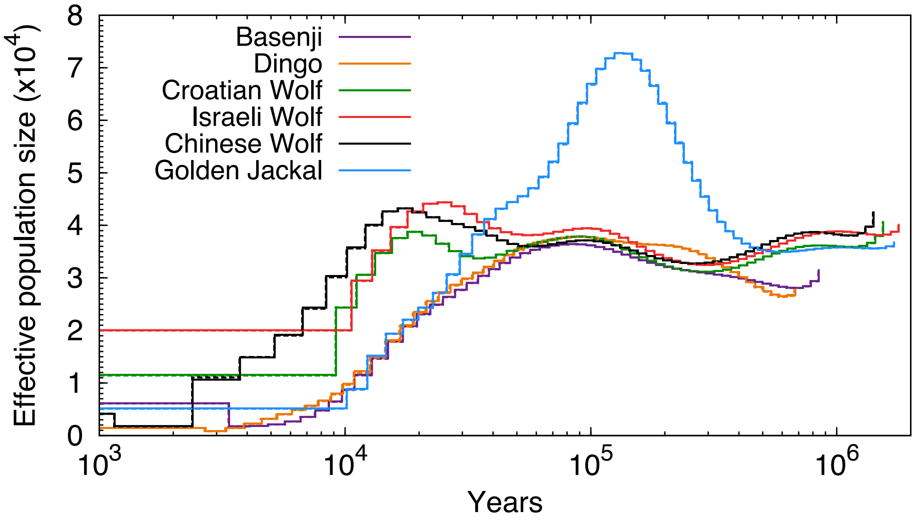

Figure S8.2.2. N_e trajectories of 6 canid lineages reconstructed using the PSMC method of Li and Durbin [3], using all the genomic information that passed our quality filters (dashed lines) and excluding 43 regions with runs of homozygosity (solid lines).

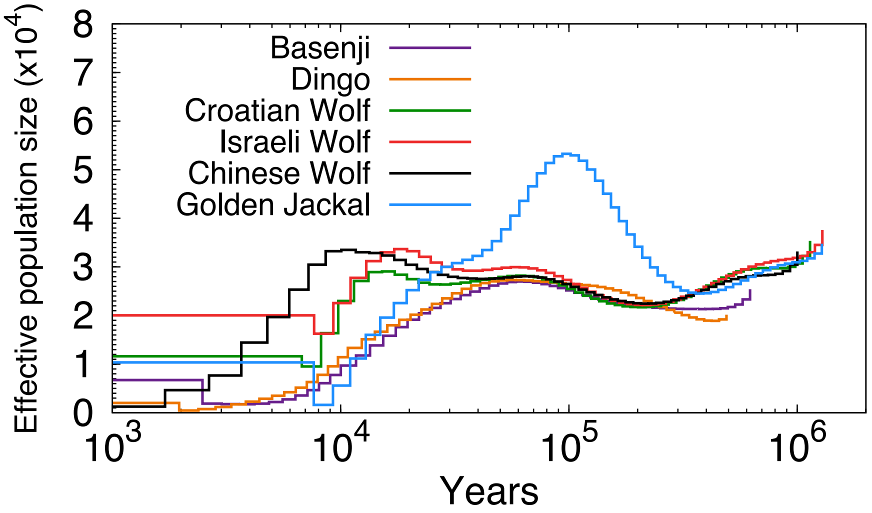

Figure S8.2.3. N_e trajectories of 6 canid lineages reconstructed using the PSMC method of Li and Durbin [3] using the sites that had a GQ >= 10 and passed the SF and GF2 filters.

There are 7 different genomes being simulated in the command lines for each scenario. They are a haploid genome of the Boxer and diploid genomes for the Basenji, Dingo, Israeli wolf, Croatian wolf, Chinese wolf and Golden Jackal, respectively. Only the diploid genomes were used in this analysis. The output of MaCS was processed using perl scripts, so that each of the 30Mb regions was transformed into a binary sequence of '1' and '0', where each character was determined by the presence or absence of a heterozygote site in contiguous windows of 100bp. Then, for each lineage we used the 100 transformed binary sequences of 30Mb to run the PSMC method using the following command line:

```
./psmc -N20 -t10 -r5 -p "1*6+58*1" -o <Output file> <Input file>.
```

The recombination rate in all scenarios was assumed to be equal to 0.92 cM/Mb, a value that is equal to the mean recombination rate estimated in the dog genome in a linkage map generated using microsatellites [7]. In these simulations, we set the generational time to 3 years and mutation rate to 1×10^{-8} per bp per generation for all species.

We compared the N_e trajectories specified in the simulations with the estimations done by the PSMC method for each canid species. Scenarios 2 (Figure S8.2.4) and 3 (Figure S8.2.5) have remarkably similar and accurate trajectories inferred using the PSMC method for all species of canids. In scenarios 4 (Figure S8.2.6), 5 (Figure S8.2.7) and 1 (Figure S8.2.8), the N_e trajectories are also accurate for all species of canids but the golden jackal, where the estimate of N_e is inflated in the interval from 10,000 - 300,000 years ago, with a distinctive sharp peak between 100,000 and 300,000 years ago.

Admixture with wolves or the ancestor of dogs and wolves appears to generate the extreme upward bias in the inferred ancestral jackal N_e. In PSMC inferences from simulated jackal demographic histories the presence of jackal - dog/wolf ancestor and jackal - Israeli wolf migration bands (Figures S8.2.6 – S8.2.8) produced an artefactual spike in the jackal N_e trajectory. This sharp peak is similar to the one observed in the empirical data from the golden jackal, although in the N_e trajectory reconstructed from that data, the peak is slightly more recent. Overall, we conclude the peak in the N_e trajectory observed in the data is likely due to post-divergence gene flow between ancestors of contemporary golden jackals and Israeli wolves or the ancestor of dogs and wolves. Ongoing work has found evidence for multiple highly divergent jackal or jackal-like lineages in Africa and the Middle East (Koepfli et al., unpublished data).

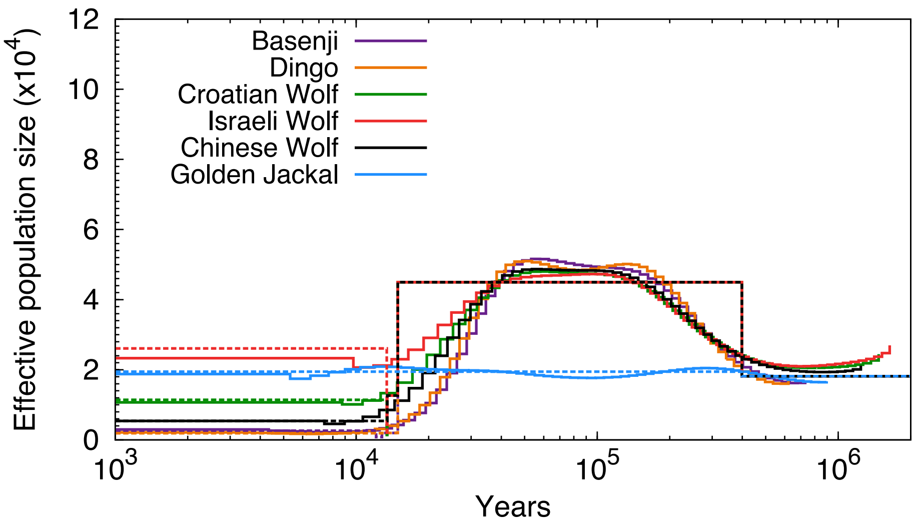

Figure S8.2.4. N_e trajectories of 6 canid lineages reconstructed using the PSMC method of Li and Durbin [3], for data simulated under the *G-PhoCS* inferred demographic history, excluding migration bands. The dotted lines show the actual N_e trajectories whereas the solid lines represent the inferred N_e trajectories.

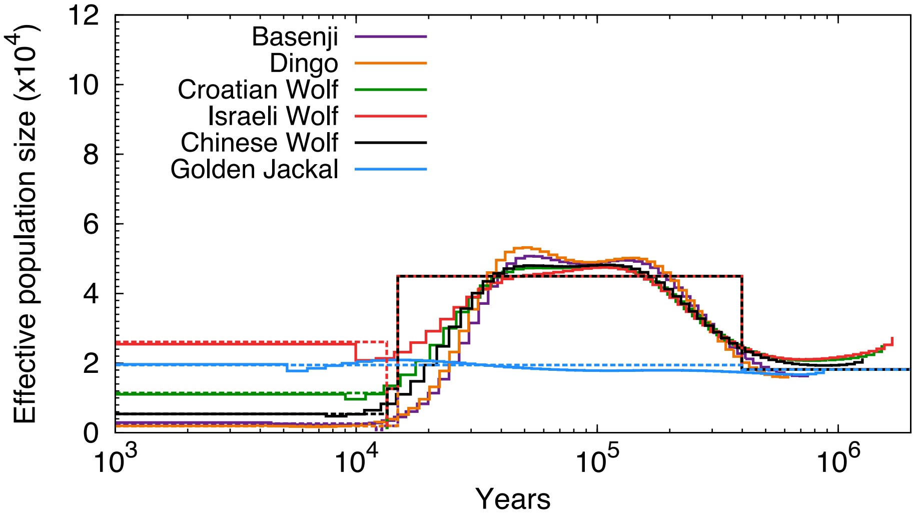

Figure S8.2.5. N_e trajectories of 6 canid lineages reconstructed using the PSMC method of Li and Durbin [3] for data simulated under the *G-PhoCS* inferred demographic history, only including gene flow from the golden jackal to the ancestor of dogs and wolves. Inferred N_e trajectories are shown with solid lines and the actual N_e trajectories are displayed with dotted lines.

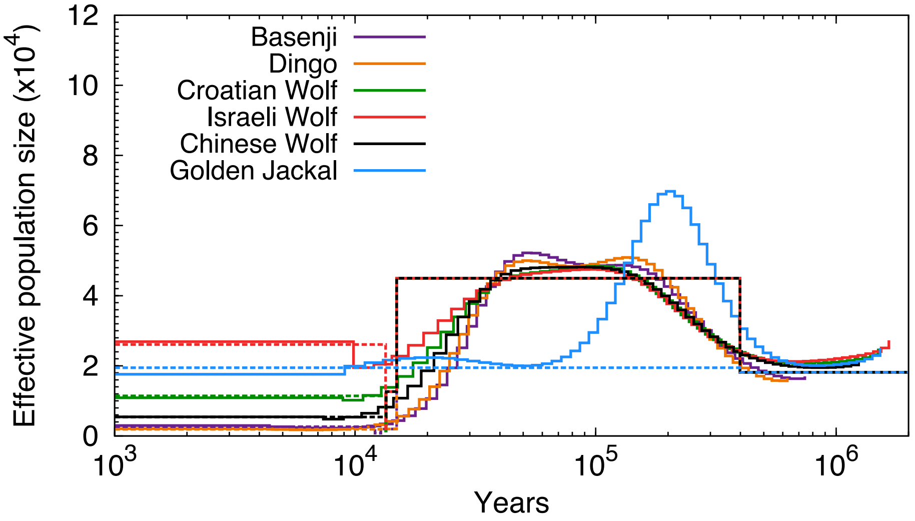

Figure S8.2.6. N_e trajectories of 6 canid lineages reconstructed using the PSMC method of Li and Durbin [3] for data simulated under the *G-PhoCS* inferred demographic history, only including gene flow from the ancestor of dogs and wolves to golden jackal. Inferred N_e trajectories are shown with solid lines and the actual N_e trajectories are displayed with dotted lines.

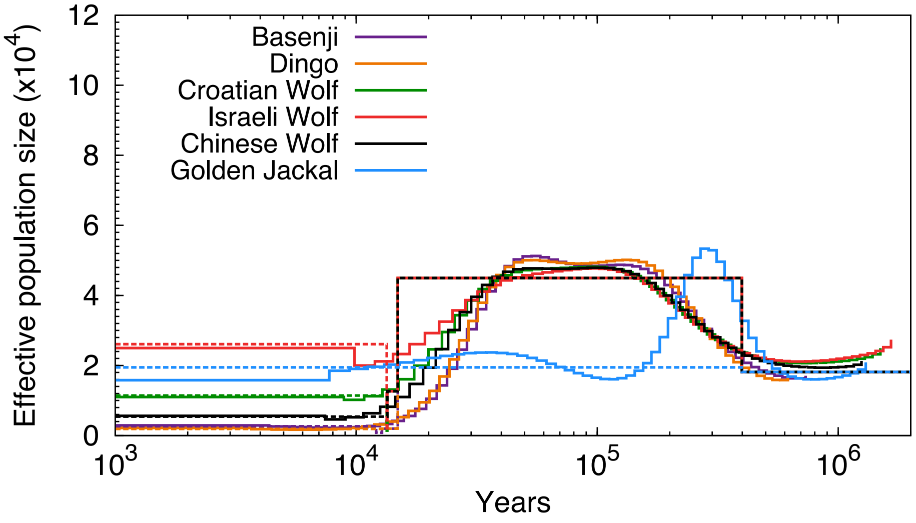

Figure S8.2.7. N_e trajectories of 6 canid lineages reconstructed using the PSMC method of Li and Durbin [3] for data simulated under the *G-PhoCS* inferred demographic history, only including gene flow from Israeli wolf to golden jackal. Inferred N_e trajectories are shown with solid lines and the actual N_e trajectories are displayed with dotted lines.

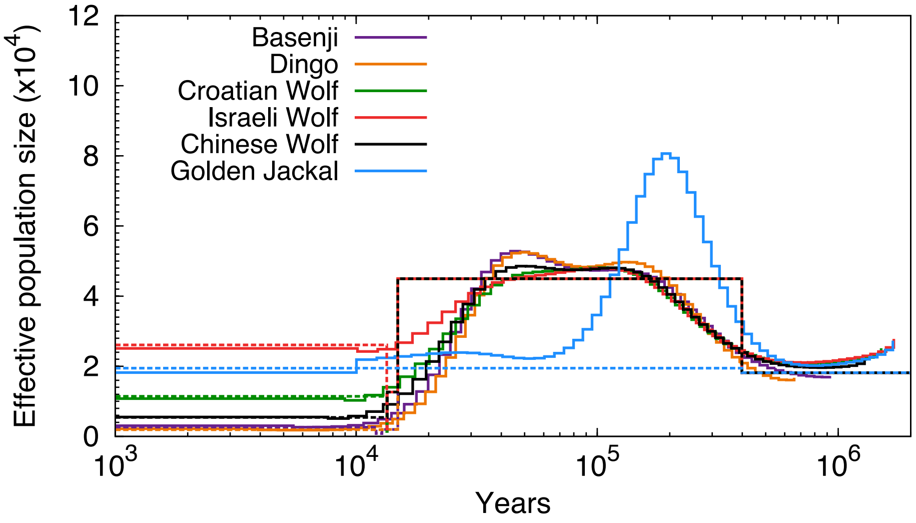

Figure S8.2.8. N_e trajectories of 6 canid lineages reconstructed using the PSMC method of Li and Durbin [3], for data simulated under the *G-PhoCS* inferred demographic history, including all detected gene flow. The actual N_e trajectories are shown as dotted lines whereas the inferred N_e trajectories are depicted by solid lines.

S8.3 Genealogies and Incomplete Lineage Sorting

S8.3.1 Definition of Neutral Loci

To assess patterns of incomplete lineage sorting, we focused on a set of neutral loci, 1kb in length, chosen so as to reduce potential confounding effects of natural selection, following guidelines set by several previous studies [1,8]. To create this set of loci, we scanned the boxer

genome, examining sliding 1kb windows with a step size of 50bp. To be included in the neutral loci set, a region had to pass the following filters: 1) no coding DNA; 2) located at least 100kb away from the nearest gene (both "known" and predicted); 3) GC content within two standard deviations of the mean GC content of the boxer genome; 4) within 1kb, no 50bp window with a PhastCons score >0.5; 5) within 1 kb, no two consecutive 50bp windows with a mappability score >2, with mappability computed using the program TALLYMER [9]; 6) no RepeatMasked elements with divergence less than 25%; and 7) no N's in boxer reference genome. Loci were further selected to be located at least 50kb from one another, leading to a total of 5139 markers, 5073 of which were autosomal. Within each locus, CpG sites present within any of the genomes were masked from further analysis in all genomes.

S8.3.2 Neighbor-joining Trees

For the above 5073 neutral loci (see Text S9.3.1) we reconstructed putative genealogies using the neighbor-joining method as implemented in PHYLIP with the pairwise differences being calculated as in E9.1.

S8.3.3 Coalescent simulations

In order to compare the distribution of genealogies to those expected under the demographic history of dogs and wolves, we simulated genealogies of 5073 1-kb segments with the program *ms* [10] under the demographic history inferred by *G-PhoCS* (see Text S9), and then built a NJ tree from this simulated data. We repeated this procedure 1,000 times using the command line (Command Line 6).

From the 1,000 simulated genealogies, we counted the proportion of those in which dogs were monophyletic, the proportion of times we observed a particular outgroup to dogs (conditional on dog monophyly), and the frequency of different outgroups to the Israeli wolf. We report this last set of statistics because previous research on dog domestication found an excess of haplotype sharing between dogs and Israeli wolves [11], and because we detected substantial admixture between the Israeli wolf and basenji (see Text S8.4). Results from simulations are all reported as the mean values of the 1,000 runs.

S8.3.4. Results

In 385 of the 5073 genealogies recovered from our neutral loci, all branch lengths were equal to 0, and we excluded these from subsequent analyses. Within the remaining 4688 genealogies, 365 (7.79%, binomial 95%CI = 7.02% - 8.55%) contained a monophyletic dog clade. For the simulated genealogies, 212 (4.23%, 95%CI = 3.69% - 4.78%) contained a monophyletic dog clade. The neutral loci and simulated data contain different proportions of trees in which dogs are monophyletic. In both the empirical and simulated data, within the set of genealogies in which dogs were monophyletic, dogs did not have clear outgroup in most trees (neutral loci: 157 trees, 3.35%; simulated genealogies 158 trees, 3.16%; labelled 'NA in Figure S8.3.4.1A). These relatively high frequencies of neutral genealogies that are discordant with the genome-wide species tree point to a combination of a) a lack of resolution due to too few mutations within a 1-kb segment to resolve relationships, and b) incomplete lineage sorting, likely due to both the relatively recent timing of divergence, and recurrent admixture between wild and domestic canids.

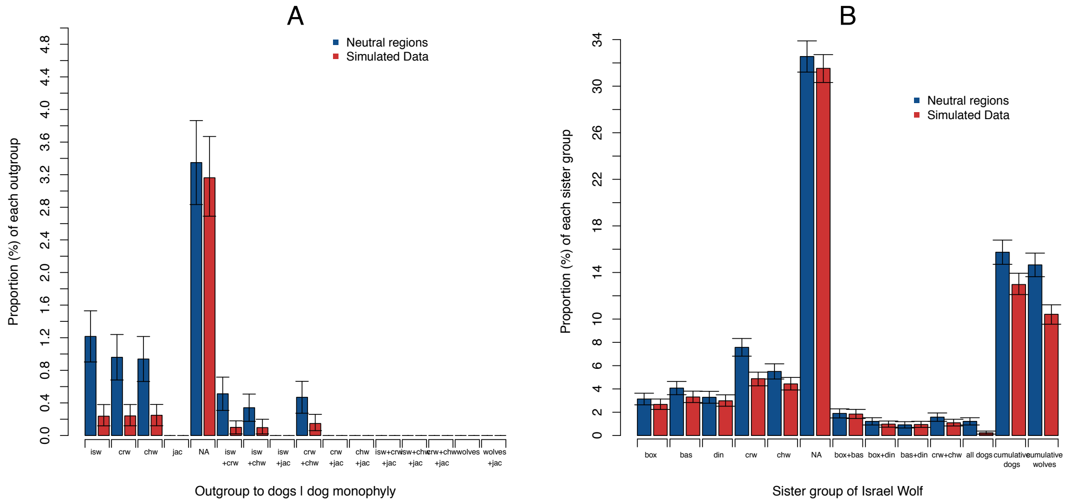

Figure S8.3.4.1. For both neutral loci extracted from sequencing data, and simulated histories inferred from *G-PhoCS*, (A) the frequency of different outgroups to dogs when dogs are recovered as a monophyletic clade in NJ trees, and (B) frequencies of outgroups to the Israeli wolf.

For the remainder of genealogies derived from empirical data, the Israeli wolf is the most common outgroup to dogs (57 trees, 1.22%), with the Croatian and Chinese wolves appearing at similar lower frequencies. In contrast, in the simulated data, the Chinese wolf is the most common outgroup to dogs (0.248%), although the proportions among the three wolves are very similar (isw: 0.237%; crw: 0.241%). Although the most frequent outgroup to dogs in neutral loci is Israeli wolf, the 95%CIs for the three wolves are overlapping (Figure 8.3.4.1A). In both neutral loci and simulated data, no trees were recovered in which a monophyletic wolf clade was sister to the dog clade. Inconsistent with the genome-wide species tree, in both the empirical and simulated data polytomies frequently preclude an assignment of an outgroup to the Israeli wolf. However, in those cases where an outgroup can be assigned, both empirical and simulated data identify the Croatian wolf as the most common outgroup to the Israeli wolf, followed by the Chinese wolf (Figure 8.3.4.1B). Consistent with Israeli wolf - Basenji admixture, of the three dogs the Basenji was the most frequent outgroup to the Israeli wolf.

S8.4 Post-Divergence Gene Flow

To investigate the extent of gene flow between wolves and dogs subsequent to their divergence, we employed a method recently developed by Durand *et al.* [12]. This method tests for gene flow by testing for asymmetries in allele sharing between a source lineage (P3), and either of two receiving lineages (P1, P2). In this case, the ancestor of P1 and P2 is sister to the ancestor of P3. Given a site that is bi-allelic in (P1, P2, P3) where P3 is in state B and an outgroup (O) is in state A, there are two possible allelic configurations of P1-P2-P3-O that are informative with respect to gene flow between P3 and either P1 or P2: ABBA and BABA. In the absence of lineage-specific post-divergence gene flow and under selective neutrality, the genome-wide frequency of these configurations should be approximately equal. Thus, the null hypothesis is that there has

not been gene flow between P3 and P1 or P2 after the divergence of P3 from P1 and P2. We defined an ABBA site as a site where P1 and the outgroup shared the same allele 'A' while P2 and P3 shared an alternative allele 'B'. A site was defined as a BABA site when the outgroup and P2 shared the allele 'A' and the alternative allele 'B' was shared between P1 and P3. The rejection of the null hypothesis indicates that there has been gene flow between P3 and either P1 or P2. Deviations from the null expectation were quantified using the D-statistic:

$$D = \frac{\sum_{i=1}^{n} C_{ABBA}(i) - \sum_{i=1}^{n} C_{BABA}(i)}{\sum_{i=1}^{n} C_{ABBA}(i) + \sum_{i=1}^{n} C_{BABA}(i)} \quad \text{(Eq 8.3)}$$

where $C_{ABBA}(i)$ and $C_{BABA}(i)$ are indicator variables equal to 1 or 0 depending on the presence or absence of the ABBA and BABA sites at the ith site. To calculate the D statistic, we specified the golden jackal as our outgroup, and divided the reference genome into 422 segments of 5 Mb each, excluding the chromosome ends where the remaining segment is < 5Mb. Within these segments, we used stringent filtering criteria, excluding genomic positions with missing data, and sites that failed either the GF2 or SF filters (see Text S4) For each species at each site, with the exception of the haploid boxer reference, we randomly sampled one allele from the called genotype. We then calculated the D statistic from a total of *n* sites that met our quality control filters.

To be consistent with the evolutionary history reflected in the recovered neighbor-joining tree (see S8.1 above), and to focus on gene flow most germane to evolutionary processes influencing wolf-dog divergence, we restricted testing to those cases where when one of the dog samples was P3, the other two (P1 and P2) were wolves, and vice versa (P3=wolf, P1 & P2 = dogs). Using these criteria, and including the boxer reference among the dogs, 18 tests were possible.

Following Durand *et al.* [12], the standard error of the statistic was calculated using a jackknife procedure [13]. A Z-score was then obtained by dividing the value of the D statistic by its standard error. Z-scores with an absolute value ≥3 were considered significant. Rejection of the null hypothesis indicates that there has been gene flow between P3 and either P1 or P2 [14]. Negative significant Z scores indicate gene flow between P1 and P3 while positive significant Z scores indicate gene flow between P2 and P3.

We found evidence for post-divergence gene flow between three pairs of samples: basenji/Israeli wolf, boxer/Israeli wolf, and dingo/Chinese wolf (Table S8.4.1). The mean absolute value of *Z* was highest in basenji/Israeli wolf ($|\hat{Z}|$ = 9.27; range = 5.64 -12.11), compared to Chinese wolf/Dingo $|\hat{Z}|$ = 6.58; range = 3.58 – 10.14), and Israeli wolf/Boxer ($|\hat{Z}|$ = 6.15; range = 5.33 - 6.71).

Because calculation of the D statistic does not account for the effects of gene flow between the outgroup and any of the three samples considered under a given test, it is possible that such gene flow could introduce bias. In particular, our analyses using *G-PhoCS* support gene flow between the jackal and the Israeli wolf and jackal and the ancestral wolf. Nevertheless, our ABBA/BABA results are not affected by this gene flow for the following reasons. First, only the gene flow with Israeli wolf could affect the calculation of the D statistic. Thus, this gene flow would not affect tests that did not include the Israeli wolf. Second, this gene flow would not affect tests with two dogs and one wolf (dog,dog,wolf,jackal = 1,2,3,4), as Israeli wolf –jackal

Table S8.4.1. Estimation of post-divergence gene flow using the D Statistic [12]. The outgroup in all comparisons is the golden jackal. Statistical significance is evaluated using a two-tailed Z test, with the additional requirement that that absolute value of the Z-score to be ≥3. Significant tests and sample pairs showing evidence for post-divergence gene flow are shown in bold.

P1	P2	P3	ABBA Sites	BABA Sites	D (%)	SE (%)	Z	p-value
Basenji	Dingo	Croatian wolf	164211	162364	0.57%	0.40%	1.42	0.16
**Basenji**	Dingo	**Israeli wolf**	158610	179656	-6.22%	0.51%	**-12.21**	**2.79×10^{-34}**
Boxer	Basenji	Croatian wolf	144942	146113	-0.40%	0.46%	-0.88	0.38
Boxer	**Basenji**	**Israeli wolf**	157007	147991	2.96%	0.52%	**5.64**	**1.67×10^{-8}**
Boxer	Dingo	Croatian wolf	177485	176031	0.41%	0.44%	0.94	0.35
**Boxer**	Dingo	**Israeli wolf**	176511	189294	-3.49%	0.52%	**-6.71**	**1.96×10^{-11}**
Croatian wolf	**Israeli wolf**	**Boxer**	226123	210897	3.48%	0.65%	**5.33**	**9.86×10^{-8}**
Croatian wolf	Israeli wolf	Dingo	213742	212876	0.20%	0.54%	0.38	0.71
Croatian wolf	**Israeli wolf**	**Basenji**	205695	182191	6.06%	0.62%	**9.74**	**1.99×10^{-22}**
Basenji	**Dingo**	**Chinese wolf**	173366	162030	3.38%	0.45%	**7.49**	**6.76×10^{-14}**
Boxer	Basenji	Chinese wolf	149172	147273	0.64%	0.41%	1.54	0.12
Boxer	**Dingo**	**Chinese wolf**	192400	175946	4.47%	0.44%	**10.14**	**3.77×10^{-24}**
Croatian wolf	Chinese wolf	Boxer	216145	219859	-0.85%	0.42%	-2.02	4.32×10^{-2}
Croatian wolf	**Chinese wolf**	**Dingo**	221737	212060	2.23%	0.44%	**5.10**	**3.48×10^{-7}**
Croatian wolf	Chinese wolf	Basenji	190706	191336	-0.16%	0.39%	-0.42	0.68
Chinese wolf	**Israeli wolf**	**Boxer**	242452	222327	4.33%	0.68%	**6.41**	**1.43×10^{-10}**
**Chinese wolf**	Israeli wolf	**Dingo**	223003	232071	-1.99%	0.56%	**-3.58**	**3.48×10^{-4}**
Chinese wolf	**Israeli wolf**	**Basenji**	216213	191475	6.07%	0.64%	**9.50**	**2.02×10^{-21}**

gene flow would lead to an allelic configuration that is **AA or **BB and thus not evaluated in the test. It is possible that, in tests with two wolves (one of which is the Israeli wolf), jackal-Israeli wolf admixture could give appearance of gene flow between the dog in question and the other wolf in the test. For example, consider a test that includes Israeli wolf, Croatian wolf, Basenji, and Golden Jackal. If the ‘B’ allele resulted from a mutation that arose in the ancestor to dogs and wolves, the original configuration would be BBBA, but Israeli wolf –jackal admixture would convert it to ABBA, leading to an upwardly biased count of this configuration, which would contribute to a Croatian wolf-Basenji gene flow signal. Nevertheless, we found in all tests with two wolves and one dog that include the Israeli wolf, the significant gene flow that is detected is between the Israeli wolf and the dog in question, the exact opposite of what would be expected if Israeli wolf –jackal gene flow were biasing the test statistic.

S8.5 Model fit using the ABBA/BABA/BBAA configurations statistics

We tested the fit of the three models analyzed with *G-PhoCS* using the proportion of sites that contain alleles that are shared between two lineages but not the other two when comparing four species. The ABBA and BABA sites are defined following the notation seen in Section S8.4. On the other hand, a BBAA site is defined as one where the lineages P_1 and P_2 share one allele while the two other lineages P_3 and O share a different allele. The proportion of those three types of sites is reflective of the genealogies contained in the data when comparing four lineages, where those genealogies are affected by gene flow and the divergence time between species. For a quartet of lineages P_1, P_2, P_3 and O we estimated the frequency of a site being ABBA, BABA or BBAA given that there are two alleles, each present in two of the four species as:

$$f(ABBA \mid two\ alleles, each\ in\ two\ species) = \frac{N(ABBA)}{N(ABBA) + N(BABA) + N(BBAA)} \quad \text{(Eq 8.4)}$$

$$f(BABA \mid two\ alleles, each\ in\ two\ species) = \frac{N(BABA)}{N(ABBA) + N(BABA) + N(BBAA)} \quad \text{(Eq 8.5)}$$

$$f(BBAA \mid two\ alleles, each\ in\ two\ species) = \frac{N(BBAA)}{N(ABBA) + N(BABA) + N(BBAA)} \quad \text{(Eq 8.6)}$$

We refer to these estimates as relative frequencies of ABBA, BABA and BBAA sites, respectively. In the equations, N(ABBA), N(BABA) and N(BBAA) are the number of ABBA, BABA and BBAA sites.

The counts of ABBA, BABA and BBAA sites in the data were calculated using the 18 quartet configurations that are shown in Table S8.4.1 with two additional quartet configurations that contain either three dogs or three wolves. Those two additional configurations were added because they are informative about the actual phylogenetic relationships inside dogs and inside wolves, respectively. A demographic model would be more likely to be correct if it captures similar values for Eq.8.4-8.6 as those seen in data. The estimates of the number of ABBA/BABA/BBAA sites in the data are shown in Table S8.5.1, along with the estimates of the relative frequency of those sites.

To mimic the empirical analysis (see above) we initially simulated 422 regions of 5Mb using the three models analyzed by *G-PhoCS.* However, because this produced an excess of ABBA/BABA/BBAA sites, to match the counts of these site classes seen in the data, we reduced our region size, instead simulating 422 regions of 2Mb. The simulations were performed using the following command lines:

1) Model where the dogs and wolves are each a separate clade (Command Line 7). This command line is identical to Command Line 1, with the only difference being the number of bases simulated.

2) Regional domestication model (Command Line 8)

3) Origin of dogs from the Israeli wolf (Command Line 9)

As a measure of the fit of each model to the data, we calculated the total difference between each model and the data in the relative frequencies of the ABBA/BABA/BBAA sites using the following equation:

$$
\begin{aligned}
\mathit{Absolute\ Error} \\
&= \sum_{i=1}^{combinations} \left| f(ABBA \mid two\ alleles, each\ in\ two\ species)_{model} \right. \\
&\left. - f(ABBA \mid two\ alleles, each\ in\ two\ species)_{data} \right| \\
&+ \left| f(BABA \mid two\ alleles, each\ in\ two\ species)_{model} \right. \\
&\left. - f(BABA \mid two\ alleles, each\ in\ two\ species)_{data} \right| \\
&+ \left| f(BBAA \mid two\ alleles, each\ in\ two\ species)_{model} \right. \\
&\left. - f(BBAA \mid two\ alleles, each\ in\ two\ species)_{data} \right|
\end{aligned}
\qquad \text{(Eq 8.7)}
$$

Overall, we found that the model which provided a better fit to the data, in terms of a smaller absolute error as estimated by Eq 8.7, was the model which assumes that the dogs and wolves are each a separate clade whereas the model which provided the worst fit was the one which assumes a regional domestication model (Table S8.5.2).

Using a threshold of 1.5% to look for important absolute differences between the data and the model in terms of relative frequencies, we found larger differences in the relative frequencies of BBAA sites in the data and the model that provided a better fit to the data in comparisons that included the Dingo, Chinese Wolf and another species of dog. We also found that the model which provided a better fit to the data incorrectly estimated the relative frequencies of ABBA sites in comparisons including the Chinese Wolf as P_1, Israeli wolf as P_2 and the Boxer or Basenji as P_3. Additionally, the number of BBAA sites in the quartet Boxer (P_1), Dingo (P_2) and Croatian Wolf (P_3) deviated substantially from those observed in the empirical data.

The regional domestication model overestimated the relative frequency of shared sites between Basenji and Dingo and underestimated the relative frequency of sites shared between (Dingo, Boxer) and (Boxer, Basenji) in comparisons that included the three dogs and the golden jackal. This shows that the phylogenetic relationships between dogs are more severely distorted under this model. This is also exemplified by the poor fit to the data in terms of the relative frequencies of ABBA/BABA/BBAA sites in the comparisons that include the Dingo, Boxer and another species of wolf. As in the model from Fig. 5A, the number of BBAA sites was also underestimated in the quartet Basenji (P_1), Dingo (P_2) and Chinese Wolf (P_3).

As with the best model, the model that posits the origin of dogs from the Israeli Wolf had poor fit to the data with respect to the relative frequency of BBAA sites in the comparisons of Boxer (P_1), Dingo (P_2) and Chinese Wolf (P_3). The latter model also had problems fitting the relative frequencies of the three types of sites we were inspecting in comparisons that included the Israeli Wolf, Croatian Wolf and a dog. The relative frequency of BBAA sites in the comparison of Boxer, Dingo and Croatian Wolf was underestimated under this model.

Table S8.5.1. Estimates of the number of ABBA/BABA/BBAA sites in the six canid genomes. For each cell and each quartet comparison we report the number of ABBA/BABA/BBAA sites followed by the frequency of those three types of sites given that the site is bi-allelic with the two alleles found in two species each. The golden jackal was used as an outgroup in all comparisons.

			Data		
**P1**	**P2**	**P3**	**ABBA Sites**	**BABA Sites**	**BBAA Sites**
Basenji	Dingo	Croatian wolf	164211; 28.43%	162364; 28.11%	250958; 43.45%
Basenji	Dingo	Israeli wolf	158610; 27.18%	179656; 30.78%	245329; 42.04%
Boxer	Basenji	Croatian wolf	144942; 24.82%	146113; 25.02%	292896; 50.16%
Boxer	Basenji	Israeli wolf	157007; 26.71%	147991; 25.17%	282873; 48.12%
Boxer	Dingo	Croatian wolf	177485; 27.15%	176031; 26.93%	300095; 45.91%
Boxer	Dingo	Israeli wolf	176511; 26.50%	189294; 28.42%	300201; 45.07%
Croatian wolf	Israeli wolf	Boxer	226123; 34.16%	210897; 31.86%	224971; 33.98%
Croatian wolf	Israeli wolf	Dingo	213742; 32.78%	212876; 32.65%	225351; 34.56%
Croatian wolf	Israeli wolf	Basenji	205695; 35.29%	182191; 31.26%	194909; 33.44%
Basenji	Dingo	Chinese wolf	173366; 29.45%	162030; 27.52%	253270; 43.02%
Boxer	Basenji	Chinese wolf	149172; 24.91%	147273; 24.59%	302448; 50.50%
Boxer	Dingo	Chinese wolf	192400; 28.40%	175946; 25.97%	309223; 45.64%
Croatian wolf	Chinese wolf	Boxer	216145; 32.52%	219859; 33.08%	228675; 34.40%
Croatian wolf	Chinese wolf	Dingo	221737; 33.97%	212060; 32.49%	218959; 33.54%
Croatian wolf	Chinese wolf	Basenji	190706; 32.79%	191336; 32.90%	199502; 34.31%
Chinese wolf	Israeli wolf	Boxer	242452; 35.42%	222327; 32.48%	219803; 32.11%
Chinese wolf	Israeli wolf	Dingo	223003; 33.37%	232071; 34.73%	213209; 31.90%
Chinese wolf	Israeli wolf	Basenji	216213; 36.43%	191475; 32.26%	185855; 31.31%
Basenji	Dingo	Boxer	179362; 32.42%	216634; 39.16%	157265; 28.43%
Chinese Wolf	Croatian Wolf	Israeli Wolf	230181; 34.70%	208597; 31.44%	224601; 33.86%

Table S8.5.2. Estimates of the number of ABBA/BABA/BBAA sites in the three *G-PhoCS* models analyzed. For each cell and each quartet comparison we report: 1) The number of ABBA/BABA/BBAA sites; 2) The frequency of those three types of sites given that the site is bi-allelic with the two alleles found in two species each and 3) the difference of that frequency in the simulations minus what is estimated in the data (when this difference is bigger than 1.5%, we highlight the cell in bold). The lower row of the table indicates the fit of the model to the data as estimated by equation 8.7. The golden jackal was used as an outgroup in all comparisons.

			**Fig. 5A model (Model where the dogs and wolves are each a separate clade)**			**Fig. 5B model (Regional domestication model)**			**Fig. 5C model (Origin of dogs from the Israeli wolf)**		
**P1**	**P2**	**P3**	**ABBA Sites**	**BABA Sites**	**BBAA Sites**	**ABBA Sites**	**BABA Sites**	**BBAA Sites**	**ABBA Sites**	**BABA Sites**	**BBAA Sites**
Basenji	Dingo	Croatian wolf	177596;<br>28.53%;<br>0.10%	180202;<br>28.95%;<br>0.84%	264624;<br>42.52%;<br>-0.94%	178773;<br>28.94%;<br>0.50%	177186;<br>28.68%;<br>0.57%	261870;<br>42.39%;<br>-1.07%	178434;<br>28.88%;<br>0.45%	177152;<br>28.67%;<br>0.56%	262289;<br>42.45%;<br>-1.00%
Basenji	Dingo	Israeli wolf	173506;<br>27.87%;<br>0.69%	191296;<br>30.72%;<br>-0.06%	257817;<br>41.41%;<br>-0.63%	173256;<br>27.83%;<br>0.65%	192556;<br>30.93%;<br>0.15%	256705;<br>41.24%;<br>-0.80%	173222;<br>27.82%;<br>0.64%	188792;<br>30.32%;<br>-0.46%	260580;<br>41.85%;<br>-0.18%
Boxer	Basenji	Croatian wolf	157926;<br>25.24%;<br>0.42%	158158;<br>25.28%;<br>0.26%	309616;<br>49.48%;<br>-0.67%	155013;<br>24.78%;<br>-0.04%	156346;<br>24.99%;<br>-0.03%	314275;<br>50.23%;<br>0.08%	158543;<br>25.42%;<br>0.60%	158872;<br>25.47%;<br>0.45%	306268;<br>49.11%;<br>-1.05%
Boxer	Basenji	Israeli wolf	168735;<br>26.93%;<br>0.23%	155221;<br>24.78%;<br>-0.40%	302524;<br>48.29%;<br>0.17%	165943;<br>26.52%;<br>-0.19%	155130;<br>24.79%;<br>-0.38%	304670;<br>48.69%;<br>0.57%	167349;<br>26.80%;<br>0.09%	155402;<br>24.89%;<br>-0.29%	301725;<br>48.32%;<br>0.20%
Boxer	Dingo	Croatian wolf	172541;<br>27.69%;<br>0.53%	175379;<br>28.14%;<br>1.21%	**275228;<br>44.17%;<br>-1.75%**	**148908;<br>23.69%;<br>-3.47%**	**148654;<br>23.64%;<br>-3.29%**	**331136;<br>52.67%;<br>6.76%**	172536;<br>27.92%;<br>0.76%	171583;<br>27.76%;<br>0.83%	**273917;<br>44.32%;<br>-1.59%**
Boxer	Dingo	Israeli wolf	173388;<br>27.77%;<br>1.27%	177664;<br>28.45%;<br>0.03%	273358;<br>43.78%;<br>-1.30%	**147173;<br>23.27%;<br>-3.24%**	**155660;<br>24.61%;<br>-3.82%**	**329753;<br>52.13%;<br>7.05%**	171562;<br>27.53%;<br>1.03%	175185;<br>28.11%;<br>-0.31%	276446;<br>44.36%;<br>-0.72%
Croatian wolf	Israeli wolf	Boxer	205879;<br>33.27%;<br>-0.89%	201724;<br>32.60%;<br>0.74%	211157;<br>34.13%;<br>0.14%	208604;<br>33.71%;<br>-0.44%	200215;<br>32.36%;<br>0.50%	209921;<br>33.93%;<br>-0.06%	208423;<br>33.80%;<br>-0.36%	**207350;<br>33.62%;<br>1.76%**	200941;<br>32.58%;<br>-1.40%
Croatian wolf	Israeli wolf	Dingo	203877;<br>32.96%;<br>0.18%	201160;<br>32.53%;<br>-0.13%	213431;<br>34.51%;<br>-0.06%	202216;<br>32.78%;<br>0.00%	202568;<br>32.84%;<br>0.19%	212020;<br>34.37%;<br>-0.19%	205800;<br>33.35%;<br>0.57%	209303;<br>33.92%;<br>1.27%	**201941;<br>32.73%;<br>-1.84%**
Croatian wolf	Israeli wolf	Basenji	215597;<br>34.74%;<br>-0.56%	197696;<br>31.85%;<br>0.59%	207361;<br>33.41%;<br>-0.03%	216547;<br>34.95%;<br>-0.35%	196012;<br>31.63%;<br>0.37%	207051;<br>33.42%;<br>-0.03%	216467;<br>35.11%;<br>-0.19%	**203118;<br>32.94%;<br>1.68%**	197038;<br>31.95%;<br>-1.49%
Basenji	Dingo	Chinese wolf	188009;<br>30.16%;<br>0.71%	177552;<br>28.49%;<br>0.96%	**257728;<br>41.35%;<br>-1.67%**	188470;<br>30.47%;<br>1.02%	174988;<br>28.29%;<br>0.77%	**254996;<br>41.23%;<br>-1.79%**	185253;<br>29.98%;<br>0.53%	173424;<br>28.06%;<br>0.54%	259312;<br>41.96%;<br>-1.06%
Boxer	Basenji	Chinese wolf	160801;<br>25.64%;<br>0.74%	158007;<br>25.20%;<br>0.61%	308245;<br>49.16%;<br>-1.34%	156840;<br>25.13%;<br>0.22%	155804;<br>24.97%;<br>0.37%	311426;<br>49.90%;<br>-0.60%	157369;<br>25.29%;<br>0.38%	159053;<br>25.56%;<br>0.97%	305845;<br>49.15%;<br>-1.35%
Boxer	Dingo	Chinese wolf	184167;<br>29.48%;<br>1.09%	170916;<br>27.36%;<br>1.40%	**269545;<br>43.15%;<br>-2.48%**	**159174;<br>25.32%;<br>-3.08%**	**144656;<br>23.01%;<br>-2.96%**	**324831;<br>51.67%;<br>6.03%**	178856;<br>28.94%;<br>0.55%	168711;<br>27.30%;<br>1.33%	**270441;<br>43.76%;<br>-1.88%**

Croatian wolf	Chinese wolf	Boxer	203311; 32.95%; 0.43%	202091; 32.76%; -0.32%	211562; 34.29%; -0.11%	200348; 32.66%; 0.14%	198041; 32.28%; -0.80%	215078; 35.06%; 0.66%	204468; 33.45%; 0.93%	203864; 33.35%; 0.27%	202947; 33.20%; -1.20%
Croatian wolf	Chinese wolf	Dingo	213747; 34.53%; 0.57%	196438; 31.74%; -0.75%	208747; 33.73%; 0.18%	209895; 34.22%; 0.25%	193324; 31.52%; -0.97%	210107; 34.26%; 0.71%	210931; 34.50%; 0.53%	201135; 32.90%; 0.41%	199265; 32.60%; -0.95%
Croatian wolf	Chinese wolf	Basenji	205710; 33.27%; 0.48%	201464; 32.58%; -0.32%	211167; 34.15%; -0.15%	201556; 32.84%; 0.05%	196880; 32.08%; -0.82%	215250; 35.07%; 0.77%	203801; 33.28%; 0.49%	204552; 33.41%; 0.50%	203964; 33.31%; -1.00%
Chinese wolf	Israeli wolf	Boxer	**208018; 33.51%; -1.91%**	205083; 33.04%; 0.56%	207667; 33.45%; 1.35%	210840; 34.03%; -1.38%	204758; 33.05%; 0.58%	203911; 32.91%; 0.81%	210065; 34.00%; -1.42%	209596; 33.92%; 1.45%	198217; 32.08%; -0.03%
Chinese wolf	Israeli wolf	Dingo	200720; 32.34%; -1.03%	215312; 34.69%; -0.04%	204645; 32.97%; 1.07%	200301; 32.34%; -1.03%	217224; 35.07%; 0.34%	201859; 32.59%; 0.69%	204194; 33.06%; -0.31%	217493; 35.21%; 0.49%	195969; 31.73%; -0.18%
Chinese wolf	Israeli wolf	Basenji	**216436; 34.81%; -1.62%**	202781; 32.61%; 0.35%	202571; 32.58%; 1.27%	217724; 35.14%; -1.29%	201865; 32.58%; 0.32%	199982; 32.28%; 0.96%	218547; 35.38%; -1.05%	204447; 33.10%; 0.84%	194752; 31.53%; 0.21%
Basenji	Dingo	Boxer	190695; 31.36%; -1.06%	242304; 39.85%; 0.69%	175036; 28.79%; 0.36%	**244189; 40.10%; 7.69%**	**219636; 36.07%; -3.08%**	**145058; 23.82%; -4.60%**	192265; 31.81%; -0.60%	237327; 39.27%; 0.12%	174739; 28.91%; 0.49%
Chinese Wolf	Croatian Wolf	Israeli Wolf	208874; 33.63%; -1.06%	203245; 32.73%; 1.28%	208912; 33.64%; -0.22%	206703; 33.38%; -1.32%	198457; 32.05%; 0.61%	214034; 34.57%; 0.71%	204824; 33.27%; -1.43%	200458; 32.56%; 1.12%	210316; 34.16%; 0.31%
		Absolute Error	0.4298			0.8219			0.4668		

Simulation Command Lines

Command Line 1. *G-PhoCS* model with the full set of migration bands inferred:

```
./macs 13 30000000 -t 1 -r 0.92 -I 7 1 2 2 2 2 2 2 -n
1 0.000010 -n 2 0.000106 -n 3 0.000077 -n 4 0.001044 -
n 5 0.000457 -n 6 0.000217 -n 7 0.000778 -m 2 4 4505.0
-m 4 2 1840.0 -m 3 6 573.0 -m 6 3 942.0 -m 4 7 58.0 -m
7 4 1162.0 -ej 0.0000403 2 1 -en 0.0000403 1 0.000032
-em 0.0000403 1 4 0.0 -em 0.0000403 4 1 0.0 -em
0.0000403 2 4 0.0 -em 0.0000403 4 2 0.0 -ej 0.0000427
3 1 -en 0.0000427 1 0.000080 -em 0.0000427 1 6 0.0 -em
0.0000427 6 1 0.0 -em 0.0000427 3 6 0.0 -em 0.0000427
6 3 0.0 -ej 0.0000446 5 4 -en 0.0000446 4 0.000056 -em
0.0000446 1 4 0.0 -em 0.0000446 4 1 0.0 -em 0.0000446
4 7 0.0 -em 0.0000446 7 4 0.0 -ej 0.0000449 6 4 -en
0.0000449 4 0.000505 -em 0.0000449 1 4 0.0 -em
0.0000449 4 1 0.0 -em 0.0000449 4 7 0.0 -em 0.0000449
7 4 0.0 -ej 0.0000496 4 1 -en 0.0000496 1 0.001800 -em
0.0000496 1 4 0.0 -em 0.0000496 4 1 0.0 -em 0.0000496
```

```
4 7 0.0 -em 0.0000496 7 4 0.0 -em 0.0000496 1 7 17.0 -
em 0.0000496 7 1 746.0 -ej 0.0013275 7 1 -en 0.0013275
1 0.000727 -em 0.0013275 1 7 0.0 -em 0.0013275 7 1 0.0
```

Command Line 2. The model inferred from *G-PhoCS* but with no gene flow between any species at any time:

```
./macs 13 30000000 -t 1 -r 0.92 -I 7 1 2 2 2 2 2 2 -n
1 0.000010 -n 2 0.000106 -n 3 0.000077 -n 4 0.001044 -
n 5 0.000457 -n 6 0.000217 -n 7 0.000778 -m 2 4 0.0 -m
4 2 0.0 -m 3 6 0.0 -m 6 3 0.0 -m 4 7 0.0 -m 7 4 0.0 -
ej 0.0000403 2 1 -en 0.0000403 1 0.000032 -em
0.0000403 1 4 0.0 -em 0.0000403 4 1 0.0 -em 0.0000403
2 4 0.0 -em 0.0000403 4 2 0.0 -ej 0.0000427 3 1 -en
0.0000427 1 0.000080 -em 0.0000427 1 6 0.0 -em
0.0000427 6 1 0.0 -em 0.0000427 3 6 0.0 -em 0.0000427
6 3 0.0 -ej 0.0000446 5 4 -en 0.0000446 4 0.000056 -em
0.0000446 1 4 0.0 -em 0.0000446 4 1 0.0 -em 0.0000446
4 7 0.0 -em 0.0000446 7 4 0.0 -ej 0.0000449 6 4 -en
0.0000449 4 0.000505 -em 0.0000449 1 4 0.0 -em
0.0000449 4 1 0.0 -em 0.0000449 4 7 0.0 -em 0.0000449
7 4 0.0 -ej 0.0000496 4 1 -en 0.0000496 1 0.001800 -em
0.0000496 1 4 0.0 -em 0.0000496 4 1 0.0 -em 0.0000496
4 7 0.0 -em 0.0000496 7 4 0.0 -em 0.0000496 1 7 0.0 -
em 0.0000496 7 1 0.0 -ej 0.0013275 7 1 -en 0.0013275 1
0.000727 -em 0.0013275 1 7 0.0 -em 0.0013275 7 1 0.0
```

Command Line 3. The model inferred from *G-PhoCS* but with only one event of gene flow, from the golden jackal to the ancestor of dogs and wolves:

```
./macs 13 30000000 -t 1 -r 0.92 -I 7 1 2 2 2 2 2 2 -n
1 0.000010 -n 2 0.000106 -n 3 0.000077 -n 4 0.001044 -
n 5 0.000457 -n 6 0.000217 -n 7 0.000778 -m 2 4 0.0 -m
4 2 0.0 -m 3 6 0.0 -m 6 3 0.0 -m 4 7 0.0 -m 7 4 0.0 -
ej 0.0000403 2 1 -en 0.0000403 1 0.000032 -em
0.0000403 1 4 0.0 -em 0.0000403 4 1 0.0 -em 0.0000403
2 4 0.0 -em 0.0000403 4 2 0.0 -ej 0.0000427 3 1 -en
0.0000427 1 0.000080 -em 0.0000427 1 6 0.0 -em
0.0000427 6 1 0.0 -em 0.0000427 3 6 0.0 -em 0.0000427
6 3 0.0 -ej 0.0000446 5 4 -en 0.0000446 4 0.000056 -em
0.0000446 1 4 0.0 -em 0.0000446 4 1 0.0 -em 0.0000446
4 7 0.0 -em 0.0000446 7 4 0.0 -ej 0.0000449 6 4 -en
0.0000449 4 0.000505 -em 0.0000449 1 4 0.0 -em
0.0000449 4 1 0.0 -em 0.0000449 4 7 0.0 -em 0.0000449
7 4 0.0 -ej 0.0000496 4 1 -en 0.0000496 1 0.001800 -em
```

```
0.0000496 1 4 0.0 -em 0.0000496 4 1 0.0 -em 0.0000496
4 7 0.0 -em 0.0000496 7 4 0.0 -em 0.0000496 1 7 17.0 -
em 0.0000496 7 1 0.0 -ej 0.0013275 7 1 -en 0.0013275 1
0.000727 -em 0.0013275 1 7 0.0 -em 0.0013275 7 1 0.0
```

Command Line 4. The model inferred from *G-PhoCS* but with only one event of gene flow, from the ancestor of dogs and wolves to golden jackal:

```
./macs 13 30000000 -t 1 -r 0.92 -I 7 1 2 2 2 2 2 2 -n 1
0.000010 -n 2 0.000106 -n 3 0.000077 -n 4 0.001044 -n 5
0.000457 -n 6 0.000217 -n 7 0.000778 -m 2 4 0.0 -m 4 2 0.0
-m 3 6 0.0 -m 6 3 0.0 -m 4 7 0.0 -m 7 4 0.0 -ej 0.0000403 2
1 -en 0.0000403 1 0.000032 -em 0.0000403 1 4 0.0 -em
0.0000403 4 1 0.0 -em 0.0000403 2 4 0.0 -em 0.0000403 4 2
0.0 -ej 0.0000427 3 1 -en 0.0000427 1 0.000080 -em
0.0000427 1 6 0.0 -em 0.0000427 6 1 0.0 -em 0.0000427 3 6
0.0 -em 0.0000427 6 3 0.0 -ej 0.0000446 5 4 -en 0.0000446 4
0.000056 -em 0.0000446 1 4 0.0 -em 0.0000446 4 1 0.0 -em
0.0000446 4 7 0.0 -em 0.0000446 7 4 0.0 -ej 0.0000449 6 4 -
en 0.0000449 4 0.000505 -em 0.0000449 1 4 0.0 -em 0.0000449
4 1 0.0 -em 0.0000449 4 7 0.0 -em 0.0000449 7 4 0.0 -ej
0.0000496 4 1 -en 0.0000496 1 0.001800 -em 0.0000496 1 4
0.0 -em 0.0000496 4 1 0.0 -em 0.0000496 4 7 0.0 -em
0.0000496 7 4 0.0 -em 0.0000496 1 7 0.0 -em 0.0000496 7 1
746.0 -ej 0.0013275 7 1 -en 0.0013275 1 0.000727 -em
0.0013275 1 7 0.0 -em 0.0013275 7 1 0.0
```

Command Line 5. The model inferred from *G-PhoCS* but with only one event of gene flow, from Israeli wolf to golden jackal:

```
./macs 13 30000000 -t 1 -r 0.92 -I 7 1 2 2 2 2 2 2 -n 1
0.000010 -n 2 0.000106 -n 3 0.000077 -n 4 0.001044 -n 5
0.000457 -n 6 0.000217 -n 7 0.000778 -m 2 4 0.0 -m 4 2 0.0
-m 3 6 0.0 -m 6 3 0.0 -m 4 7 0.0 -m 7 4 1162.0 -ej
0.0000403 2 1 -en 0.0000403 1 0.000032 -em 0.0000403 1 4
0.0 -em 0.0000403 4 1 0.0 -em 0.0000403 2 4 0.0 -em
0.0000403 4 2 0.0 -ej 0.0000427 3 1 -en 0.0000427 1
0.000080 -em 0.0000427 1 6 0.0 -em 0.0000427 6 1 0.0 -em
0.0000427 3 6 0.0 -em 0.0000427 6 3 0.0 -ej 0.0000446 5 4 -
en 0.0000446 4 0.000056 -em 0.0000446 1 4 0.0 -em 0.0000446
4 1 0.0 -em 0.0000446 4 7 0.0 -em 0.0000446 7 4 0.0 -ej
0.0000449 6 4 -en 0.0000449 4 0.000505 -em 0.0000449 1 4
0.0 -em 0.0000449 4 1 0.0 -em 0.0000449 4 7 0.0 -em
```

```
0.0000449 7 4 0.0 -ej 0.0000496 4 1 -en 0.0000496 1
0.001800 -em 0.0000496 1 4 0.0 -em 0.0000496 4 1 0.0 -em
0.0000496 4 7 0.0 -em 0.0000496 7 4 0.0 -em 0.0000496 1 7
0.0 -em 0.0000496 7 1 0.0 -ej 0.0013275 7 1 -en 0.0013275 1
0.000727 -em 0.0013275 1 7 0.0 -em 0.0013275 7 1 0.0
```

Command Line 6. *ms* command line that uses the demographic history estimated from *G-PhoCS*.

```
./ms 7 1 -t 1000 -r 920 1000 -I 7 1 1 1 1 1 1 1 -n 1
0.000010 -n 2 0.000106 -n 3 0.000077 -n 4 0.001044 -n 5
0.000457 -n 6 0.000217 -n 7 0.000778 -m 2 4 4505.0 -m 4 2
1840.0 -m 3 6 573.0 -m 6 3 942.0 -m 4 7 58.0 -m 7 4 1162.0
-ej 0.0000403 2 1 -en 0.0000403 1 0.000032 -em 0.0000403 1
4 0.0 -em 0.0000403 4 1 0.0 -em 0.0000403 2 4 0.0 -em
0.0000403 4 2 0.0 -ej 0.0000427 3 1 -en 0.0000427 1
0.000080 -em 0.0000427 1 6 0.0 -em 0.0000427 6 1 0.0 -em
0.0000427 3 6 0.0 -em 0.0000427 6 3 0.0 -ej 0.0000446 5 4 -
en 0.0000446 4 0.000056 -em 0.0000446 1 4 0.0 -em 0.0000446
4 1 0.0 -em 0.0000446 4 7 0.0 -em 0.0000446 7 4 0.0 -ej
0.0000449 6 4 -en 0.0000449 4 0.000505 -em 0.0000449 1 4
0.0 -em 0.0000449 4 1 0.0 -em 0.0000449 4 7 0.0 -em
0.0000449 7 4 0.0 -ej 0.0000496 4 1 -en 0.0000496 1
0.001800 -em 0.0000496 1 4 0.0 -em 0.0000496 4 1 0.0 -em
0.0000496 4 7 0.0 -em 0.0000496 7 4 0.0 -em 0.0000496 1 7
17.0 -em 0.0000496 7 1 746.0 -ej 0.0013275 7 1 -en
0.0013275 1 0.000727 -em 0.0013275 1 7 0.0 -em 0.0013275 7
1 0.0
```

Command Line 7. Model where the dogs and wolves are each a separate clade, identical to Command Line 1, except for the simulation of smaller (2Mb) genomic regions.

```
./macs 13 2000000 -t 1 -r 0.92 -I 7 1 2 2 2 2 2 2 -n 1
0.000010 -n 2 0.000106 -n 3 0.000077 -n 4 0.001044 -n
5 0.000457 -n 6 0.000217 -n 7 0.000778 -m 2 4 4505.0 -
m 4 2 1840.0 -m 3 6 573.0 -m 6 3 942.0 -m 4 7 58.0 -m
7 4 1162.0 -ej 0.0000403 2 1 -en 0.0000403 1 0.000032
-em 0.0000403 1 4 0.0 -em 0.0000403 4 1 0.0 -em
0.0000403 2 4 0.0 -em 0.0000403 4 2 0.0 -ej 0.0000427
3 1 -en 0.0000427 1 0.000080 -em 0.0000427 1 6 0.0 -em
0.0000427 6 1 0.0 -em 0.0000427 3 6 0.0 -em 0.0000427
6 3 0.0 -ej 0.0000446 5 4 -en 0.0000446 4 0.000056 -em
0.0000446 1 4 0.0 -em 0.0000446 4 1 0.0 -em 0.0000446
4 7 0.0 -em 0.0000446 7 4 0.0 -ej 0.0000449 6 4 -en
0.0000449 4 0.000505 -em 0.0000449 1 4 0.0 -em
0.0000449 4 1 0.0 -em 0.0000449 4 7 0.0 -em 0.0000449
7 4 0.0 -ej 0.0000496 4 1 -en 0.0000496 1 0.001800 -em
0.0000496 1 4 0.0 -em 0.0000496 4 1 0.0 -em 0.0000496
```

```
4 7 0.0 -em 0.0000496 7 4 0.0 -em 0.0000496 1 7 17.0 -
em 0.0000496 7 1 746.0 -ej 0.0013275 7 1 -en 0.0013275
1 0.000727 -em 0.0013275 1 7 0.0 -em 0.0013275 7 1 0.0
```

Command Line 8. Regional domestication model.

```
./macs 13 2000000 -t 1 -r 0.92 -I 7 1 2 2 2 2 2 2 -n 1
0.000010 -n 2 0.000128 -n 3 0.000032 -n 4 0.000889 -n
5 0.000565 -n 6 0.000171 -n 7 0.000771 -m 1 2 20054 -m
2 1 59 -m 1 3 3459 -m 3 1 9560 -m 2 3 51 -m 3 2 7618 -
m 4 5 5276 -m 5 4 48 -m 4 6 19 -m 6 4 4958 -m 5 6 26 -
m 6 5 5312 -m 4 7 182.0 -m 7 4 1207.0 -ej 0.0000478 4
2 -en 0.0000478 2 0.000437 -em 0.0000478 1 2 0.0 -em
0.0000478 2 1 0.0 -em 0.0000478 2 3 0.0 -em 0.0000478
3 2 0.0 -em 0.0000478 4 5 0.0 -em 0.0000478 5 4 0.0 -
em 0.0000478 4 6 0.0 -em 0.0000478 6 4 0.0 -em
0.0000478 4 7 0.0 -em 0.0000478 7 4 0.0  -ej 0.0000614
5 1 -en 0.0000614 1 0.000162 -em 0.0000478 1 2 0.0 -em
0.0000478 2 1 0.0 -em 0.0000478 1 3 0.0 -em 0.0000478
3 1 0.0 -em 0.0000478 4 5 0.0 -em 0.0000478 5 4 0.0 -
em 0.0000478 5 6 0.0 -em 0.0000478 6 5 0.0 -ej
0.0000617 6 3 -en 0.0000617 3 0.000017 -em 0.0000478 3
2 0.0 -em 0.0000478 2 3 0.0 -em 0.0000478 1 3 0.0 -em
0.0000478 3 1 0.0 -em 0.0000478 6 5 0.0 -em 0.0000478
5 6 0.0 -em 0.0000478 4 6 0.0 -em 0.0000478 6 4 0.0 -
ej 0.0000618 2 1 -en 0.0000618 1 0.000252 -ej
0.0000626 3 1 -en 0.0000626 1 0.001790 -em 0.0000626 1
7 3.0 -em 0.0000626 7 1 782.0 -ej 0.0013859 7 1 -en
0.0013859 1 0.000682 -em 0.0013859 1 7 0.0 -em
0.0013859 7 1 0.0
```

Command Line 9. Origin of dogs from the Israeli wolf.

```
./macs 13 2000000 -t 1 -r 0.92 -I 7 1 2 2 2 2 2 2 -n 1
0.000010 -n 2 0.000103 -n 3 0.000076 -n 4 0.000894 -n
5 0.000445 -n 6 0.000221 -n 7 0.000765 -m 2 4 5032.0 -
m 4 2 1196.0 -m 3 6 865.0 -m 6 3 524.0 -m 4 7 142.0 -m
7 4 1063.0 -ej 0.0000401 2 1 -en 0.0000401 1 0.000025
-em 0.0000401 1 4 0.0 -em 0.0000401 4 1 0.0 -em
0.0000401 2 4 0.0 -em 0.0000401 4 2 0.0 -ej 0.0000419
3 1 -en 0.0000419 1 0.000029 -em 0.0000419 1 6 0.0 -em
0.0000419 6 1 0.0 -em 0.0000419 3 6 0.0 -em 0.0000419
6 3 0.0 -ej 0.0000444 4 1 -en 0.0000444 1 0.000186 -em
0.0000444 1 4 0.0 -em 0.0000444 4 1 0.0 -em 0.0000444
4 7 0.0 -em 0.0000444 7 4 0.0 -ej 0.0000447 5 1 -en
```

```
0.0000447 1 0.000229 -em 0.0000447 1 4 0.0 -em
0.0000447 4 1 0.0 -em 0.0000447 4 7 0.0 -em 0.0000447
7 4 0.0 -ej 0.0000450 6 1 -en 0.0000450 1 0.001801 -em
0.0000450 1 4 0.0 -em 0.0000450 4 1 0.0 -em 0.0000450
4 7 0.0 -em 0.0000450 7 4 0.0 -em 0.0000450 1 7 5.0 -
em 0.0000450 7 1 778.0 -ej 0.0013954 7 1 -en 0.0013954
1 0.000663 -em 0.0013954 1 7 0.0 -em 0.0013954 7 1 0.0
```

References

1. Gronau I, Hubisz MJ, Gulko B, Danko CG, Siepel A (2011) Bayesian inference of ancient human demography from individual genome sequences. Nature Genetics 43: 1031-U1151.
2. Felsenstein J (1989) PHYLIP - Phylogeny Inference Package (Version 3.2). Cladistics 5: 164.
3. Li H, Durbin R (2011) Inference of human population history from individual whole-genome sequences. Nature 475: 493-U484.
4. Lindblad-Toh K, Wade CM, Mikkelsen TS, Karlsson EK, Jaffe DB, et al. (2005) Genome sequence, comparative analysis and haplotype structure of the domestic dog. Nature 438: 803-819.
5. Price AL, Patterson NJ, Plenge RM, Weinblatt ME, Shadick NA, et al. (2006) Principal components analysis corrects for stratification in genome-wide association studies. Nature Genetics 38: 904-909.
6. Chen GK, Marjoram P, Wall JD (2009) Fast and flexible simulation of DNA sequence data. Genome Research 19: 136-142.
7. Wong AK, Ruhe AL, Dumont BL, Robertson KR, Guerrero G, et al. (2010) A Comprehensive Linkage Map of the Dog Genome. Genetics 184: 595-U436.
8. Wall JD, Cox MP, Mendez FL, Woerner A, Severson T, et al. (2008) A novel DNA sequence database for analyzing human demographic history. Genome Research 18: 1354-1361.
9. Kurtz S, Narechania A, Stein JC, Ware D (2008) A new method to compute K-mer frequencies and its application to annotate large repetitive plant genomes. Bmc Genomics 9.
10. Hudson RR (2002) Generating samples under a Wright-Fisher neutral model of genetic variation. Bioinformatics 18: 337-338.
11. vonHoldt BM, Pollinger JP, Lohmueller KE, Han EJ, Parker HG, et al. (2010) Genome-wide SNP and haplotype analyses reveal a rich history underlying dog domestication. Nature 464: 898-U109.
12. Durand EY, Patterson N, Reich D, Slatkin M (2011) Testing for Ancient Admixture between Closely Related Populations. Molecular Biology and Evolution 28: 2239-2252.
13. Efron B (1981) Nonparametric Estimates of Standard Error - the Jackknife, the Bootstrap and Other Methods. Biometrika 68: 589-599.
14. Rasmussen M, Guo XS, Wang Y, Lohmueller KE, Rasmussen S, et al. (2011) An Aboriginal Australian Genome Reveals Separate Human Dispersals into Asia. Science 333: 94-98.
