# Supplementary material for: Genome Sequencing Highlights the Dynamic Early History of Dogs
Source: PLoS Genet. 2014 Jan 16;10(1):e1004016. doi: 10.1371/journal.pgen.1004016 (PMC3894170; doi:10.1371/journal.pgen.1004016)
Supplement: Text S9 — Methods and supporting results for demographic analyses using G-PhoCS. (PDF) [file pgen.1004016.s028.pdf]

S9 Demographic Analysis Using *G-PhoCS*

Ilan Gronau[1], Adam H. Freedman[2], Robert K. Wayne[2], John Novembre[2], Adam Siepel[1]

[1]Cornell University,
Department of Biological Statistics and Computational Biology
Ithaca, New York, United States of America

[2]University of California, Los Angeles
Department of Ecology and Evolutionary Biology
Los Angeles, California, United States of America

S9.1 Overview of *G-PhoCS*

Our main demographic analysis is based on the Generalized Phylogenetic Coalescent Sampler (*G-PhoCS*) developed by Gronau *et al.* [1]. *G-PhoCS* performs demographic inference conditioned on a given population phylogeny augmented by a collection of migration bands (see Fig. S9.1.1). Migration bands describe scenarios of post-divergence gene flow in the demographic model, and are defined by ordered pairs of branches in the population phylogeny, allowing different rates to be associated with the two directions of gene flow. *G-PhoCS* infers demographic parameters associated with the population phylogeny (i.e., ancestral population sizes, population divergence times, and migration rates) based on inferred genealogies at

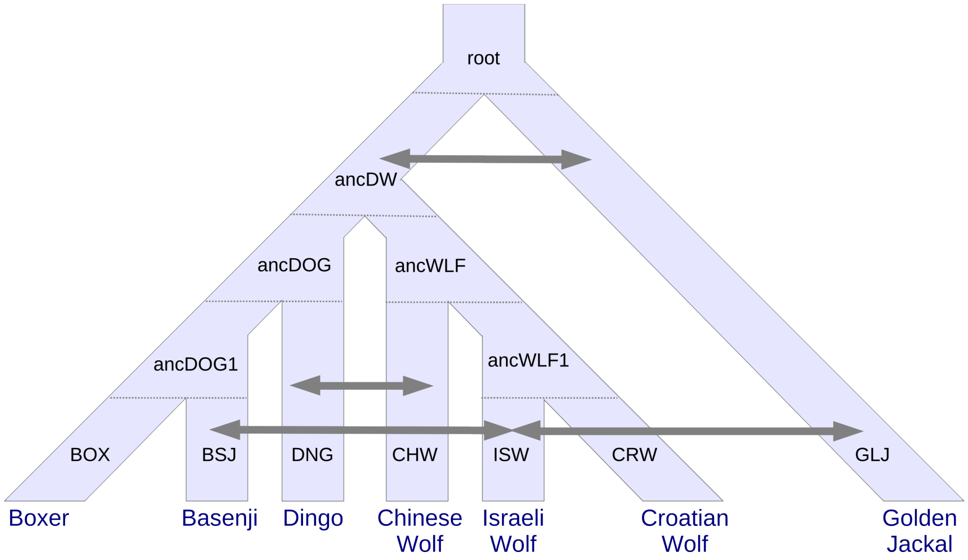

Figure S9.1.1: Population phylogeny assumed in main *G-PhoCS* demographic inference. The six genome samples and the reference genome (boxer) are indicated at the tips of the tree. Each branch in the tree is shown with its population label. The population phylogeny consists of a dog clade, a wolf clade and a jackal outgroup. Within the dog clade, boxer and basenji are assumed to be sister taxa, and within the wolf clade, the Croatian wolf and Israeli wolf are assumed to be sister taxa. The topology of the tree was inferred by Neighbor-Joining using average pairwise genomic divergences (Fig. 4; see also Text S8). Alternative topologies were considered as well (see Section S9.6). Parameters of the demographic model include effective population sizes for all branches in the tree, divergence times for all internal nodes, and migration rates for the migration bands assumed in the analysis. Bidirectional arrows represent the eight migration bands assumed in our main analysis (see Section S9.3).

thousands of neutrally evolving loci along the genome. To estimate these genealogies, *G-PhoCS* receives as input a collection of multiple sequence alignments of individual genomes at a given set of genomic loci, selected to reduce the effects of selection and sequencing error (see Section S9.2.1). Each genome in the input set is associated with a certain sampled population (terminal branch of the input phylogeny). *G-PhoCS* can analyze haploid genomes, such as the boxer reference genome (CanFam3), as well as diploid genomes, such as the six genomes sequenced in this study. Heterozygous genotypes are given in an unphased manner, and the likelihood computation analytically sums over all possible phasings.

Inference is achieved by jointly sampling values for the demographic parameters and local genealogies according to an approximate posterior distribution conditioned on the multiple sequence alignments and the input phylogeny. The method uses a full probabilistic model of coalescent with migration, and a Markov Chain Monte Carlo (MCMC) sampling strategy. The probabilistic model assumes a separate constant population size for each branch of the population phylogeny, and a separate constant migration rate for each migration band. All demographic parameters are scaled by mutation rate, which is allowed to vary across loci. Translation of parameters to absolute values, divergence times in years and population sizes in individual counts, is done by assuming a certain average neutral mutation rate and an average generation time (see Section S9.2.3).

S9.2 Sequence Data and Analysis Setup

S9.2.1 Alignments at Putative Neutral Regions

We followed a similar procedure to that described by Gronau *et al.* [1] in defining the set of loci on which to run the demographic analysis. We first filtered out regions covered by the genomic filter GF2 (see Text S4), namely, regions in the CanFam3 genome with assembly gaps, repeats, low mappabilty, and regions where none of the six sequenced genomes had reliable sequence data (Table S9.1.1). In addition, we removed regions of the genome that were likely to have evolved under the effect of strong natural selection. In particular, we filtered out exons of protein

Table S9.1.1. Data Filters used in *G-PhoCS* analysis.

Filter name	Type	Genome % [a]	Description
mappability	mappability	2.7%	Consecutive pairs of 50 bp blocks with mean mappability score > 2
repeatMasker25	mappability	24.7%	Regions with RepeatMasker score <= 25
refGaps	assembly gaps	5.3%	Sites identified as gaps in the CanFam3 assembly
maskIntersection	missing data	18.6%	Sites with no confident genotype in any of the six sequenced genomes [b]
genesAndFlanks10kb	non-neutral	42.6%	Exons of protein coding genes (see Text S7) and 10kb flanking each exon on each side
phastConsAndFlanks100b	non-neutral	12.5%	phastCons elements computed for eleven euarchontoglire mammals in the the 30-way alignment for the mouse reference, and the 100 bp flanking each element on each side
allFilters		68.7%	Union of all filters

[a] Percent of the CanFam3 genome covered by this filter.

[b] Individual genomes are filtered using the SF filter (see Text S4).

coding genes and the 10 kilobases (kb) flanking them on each side, as well as conserved non-coding elements (CNEs) and the 100 bases on each side of these elements. CNEs were defined using a conservation track for eleven euarchontoglire mammals computed using the 30-way genome alignment with mouse reference downloaded from the UCSC Genome Browser (see Text S7). Removing flanking regions around genes and CNEs reduces potential biases from selection at linked sites (e.g., background selection and hitchhiking) on our analysis (see also Section S9.7.3). After filtering, 31.3% of the CanFam3 genome remained, from which we selected 1 kb loci located at least 30 kb apart. We chose a locus length of 1 kb, because it is expected to result in small amounts of intra-locus recombination in the time scale of dog and wolf evolution (see Section S9.7.2). The inter-locus distance of 30 kb was chosen to ensure sufficient inter-locus recombination to reduce the correlation between the local genealogies at different loci.

We identified a collection of 16,434 loci that obey these criteria, and extracted multiple sequence alignments for these loci using sequence data from the six individual genomes in addition to the boxer reference (CanFam3). We further masked each genome individually for positions where there was no confident genotype call (SF filter; see Text S4). In order to avoid biases from hypermutable CpGs, we masked out all position pairs having a “CG” dinucleotide in any of the six genomes or the boxer reference genome sequence [1]. To avoid possible ancestral CpGs, we also masked out position pairs with a C* dinucleotide in one genome and *G in another. Our main set of estimates was obtained by jointly analyzing the full set of 16,434 loci. However, to expedite the supporting analyses presented in this supplement, we used a subset of 5,478 loci obtained by selecting every third locus in the original set.

S9.2.2 MCMC Setup for *G-PhoCS*

All MCMC runs were executed using the same setup, unless otherwise indicated. The prior distribution over model parameters was defined by a product of Gamma distributions. We used the default settings chosen by Gronau *et al.* [1]: a Gamma distribution with α=1.0 and β=10,000 for the mutation-scaled population sizes and divergence times, and a Gamma distribution with α=0.002 and β=0.00001 for the mutation-scaled migration rates. Each Markov Chain was run for 100,000 burn-in iterations, after which parameter values were sampled for 200,000 iterations every 10 iterations, resulting in a total of 20,001 samples from the approximate posterior. Convergence was inspected manually for each run. The finetune parameters of the sampling procedure were set automatically during the first 10,000 burn-in iterations (using the 'find-finetunes TRUE' option in the *G-PhoCS* control file).

S9.2.3 Parameter Calibration

Parameters in the probabilistic model of *G-PhoCS* are scaled by mutation rate μ. Effective population sizes are given by $\theta=4N_e\mu$, and divergence times are given by $\tau=T\mu/g$, where N_e is the absolute effective population size (in number of individuals), g is the average generation time (in years), and T is the absolute divergence time (in years). Following Lindblad-Toh *et al.* (2005), we assumed an average mutation rate of $\mu=1.0\times10^{-8}$ mutations per site per generation, and an average generation time of g=3 years. Throughout this section, we follow the convention

of discussing the calibrated estimates (N_e and T) in the text and showing both the raw estimates and calibrated values in figures and tables. For better readability, we scale up the raw estimates (τ and θ) by an additional factor of 10^4, and scale down the calibrated estimates (N_e and T) by a factor of 10^{-3}. The probablistic model of *G-PhoCS* also uses a scaled version of migration rate, $M=m/\mu$, where m is the probability of migration across a given band in a single generation. The level of gene flow across a given migration band is measured by the *total migration rate*, which is the migration rate scaled by the time span of the migration band (τ_m): $m^{tot} = M\tau_m$. If m^{tot} is sufficiently small (m^{tot}<0.5), then it approximately equals the probability that a given lineage will migrate through the band. By scaling the rate M with the time span τ_m, we obtain a measure that is independent of our assumptions on mutation rate. The time span of a migration band is defined using the start and end times of the two populations that define it. For example, the time span of the migration band from BSJ to ISW is $\min\{\tau_{ancWLF1}, \tau_{ancDOG1}\}$, and the time span of the migration band from GLJ to the ancestral population ancDW is $\tau_{root} - \tau_{ancDW}$.

S9.3 Inferring Gene Flow

The unique advantage of *G-PhoCS* is its capability to detect and measure gene flow throughout the history of the sampled populations by introducing migration bands to the demographic model. A limitation of this approach is that demographic models with large numbers of migration bands often have identifiability issues that can lead to spurious inference of migration events. To address the challenge of detecting the significant signals of gene flow in the data, we followed a strategy of examining a large number of migration bands by partitioning them across seven separate *G-PhoCS* analyses. Each of these separate analyses was conducted on the set of 5,478 neutral loci described in Section S9.2.1 using the settings described in Section S9.2.2. A migration band was inferred to have significant gene flow if the 95% Bayesian credible interval of the total migration rate for that band did not include 0, or if the total migration rate was estimated to be greater than 0.03 with posterior probability greater than 50%. We used this somewhat lax criterion for significance to ensure that we accounted for all scenarios of gene flow that have some support in the data. We then executed an additional *G-PhoCS* analysis incorporating all migration bands with significant gene flow, as well as migration bands in the opposite direction.

S9.3.1 Identifying Migration Bands with Significant Gene Flow

First, we examined gene flow between dogs and wolves by considering the 18 directional migration bands between one of the three sampled dog populations (BSJ, BOX, and DNG) and one of the three sampled wolf populations (ISW, CRW, and CHW). We conducted six separate analyses labeled according to the six sampled dog and wolf populations: the analysis labeled by population X contained the six migration bands that contain population X. Note that each of the 18 migration bands is covered in two separate *G-PhoCS* runs: the run labeled by the dog population in that band, and the run labeled by the wolf population. Thus, for each of the nine dog-wolf pairs, we recorded four migration intensities: two for the dog-to-wolf migration band, and two for the band in the opposite direction (Fig. S9.3.1A). Significant gene flow was inferred for the two migration bands between ISW and BSJ and the migration band DNG-to-CHW,

consistently in both runs that included each of these migration bands. The migration band BOX-to-ISW was inferred to have a significant total rate of 0.1 (0.045–0.155) in the 'BOX' analysis, but not in the 'ISW' analysis. This observation is consistent with gene flow from BSJ to ISW, which, in the absence of a migration band between BSJ and ISW, is likely to be inferred as gene flow from BOX to ISW. Migration bands CHW-to-DNG, and ISW-to-DNG were inferred to have nonnegligible (but insignificant) total rates of 0.021 (0–0.055) and 0.023 (0–0.058) (resp.) in one of the runs that contained each of them. We conclude that significant gene flow occurred between Israeli wolf and basenji (in both directions), and from dingo to Chinese wolf. Note that our findings are consistent with the non-parametric ABBA/BABA tests for gene flow (see Text

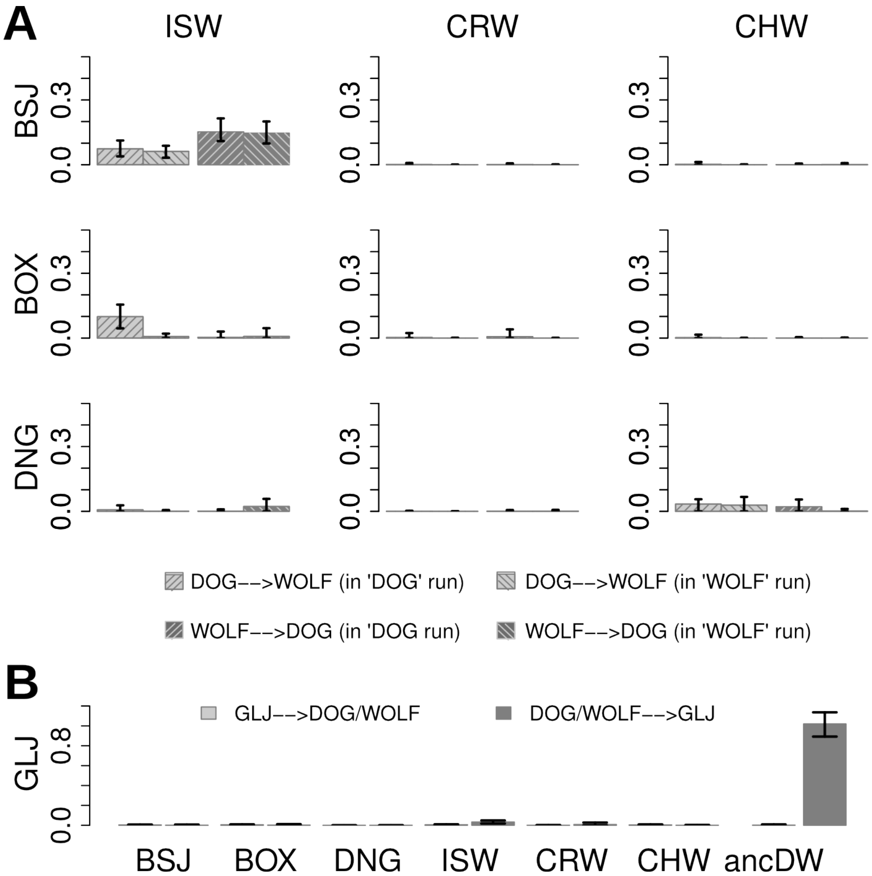

Figure S9.3.1: Identifying Migration Bands with Significant Gene Flow. A collection of 32 migration bands was examined in seven separate *G-PhoCS* analyses of the set of 5,478 neutral loci defined in Section S9.2.1 using the default MCMC settings described in Section S9.2.2. (A) Total migration rates estimated for the 18 migration bands between the sampled populations of dogs and wolves are shown with 95% Bayesian credible intervals. For each pair of dog and wolf sampled population, the left pair of bars corresponds to the DOG-to-WOLF migration band, and the right pair corresponds to the band in the opposite direction. For each migration band, the left bar indicates the total rate inferred in the analysis containing bands associated with the dog population, and the right bar corresponds to the analysis associated with the wolf population. We find significant evidence for gene flow along migration bands BSJ-to-ISW, ISW-to-BSJ, and DNG-to-CHW (see text). (B) Total migration rates inferred for 14 migration bands with GLJ. For each of the seven populations considered, rates are shown for the migration band from GLJ to that population (left) and the band in the opposite direction (right). We find significant evidence for gene flow along migration bands ISW-to-GLJ and ancDW-to-GLJ.

S8), but the ability to consider several migration bands in a single analysis allowed us to explain the positive ABBA/BABA signal observed for boxer and Israeli wolf as a result of gene flow from basenji to Israeli wolf.

Using the migration model of *G-PhoCS*, we were also able to model gene flow between the jackal outgroup and each of the other six samples. We conducted another analysis with 14 additional directional migration bands: twelve between GLJ and the other six sampled populations, and two between GLJ and the population, ancDW, ancestral to all dogs and wolves (Fig. S9.3.1B). We inferred a very high total migration rate of 1.02 (0.89–1.14) for the ancDW-to-GLJ migration band, and a smaller, but significant total rate of 0.033 (0.018–0.049) for the ISW-to-GLJ migration band.

S9.3.2 The effect of Gene Flow on Parameter Estimates

We found evidence for significant gene flow between four pairs of populations in our demographic model: (ISW,BSJ), (CHW,DNG), (GLJ,ISW), and (GLJ,ancDW). For all pairs other than (ISW,BSJ), significant gene flow was inferred only in one direction. However, to ensure we account for all plausible scenarios of gene flow, we kept all eight directional migration bands associated with these four pairs in our subsequent analysis. In order to test the effect of gene flow on estimates of population divergence times and effective population sizes, we

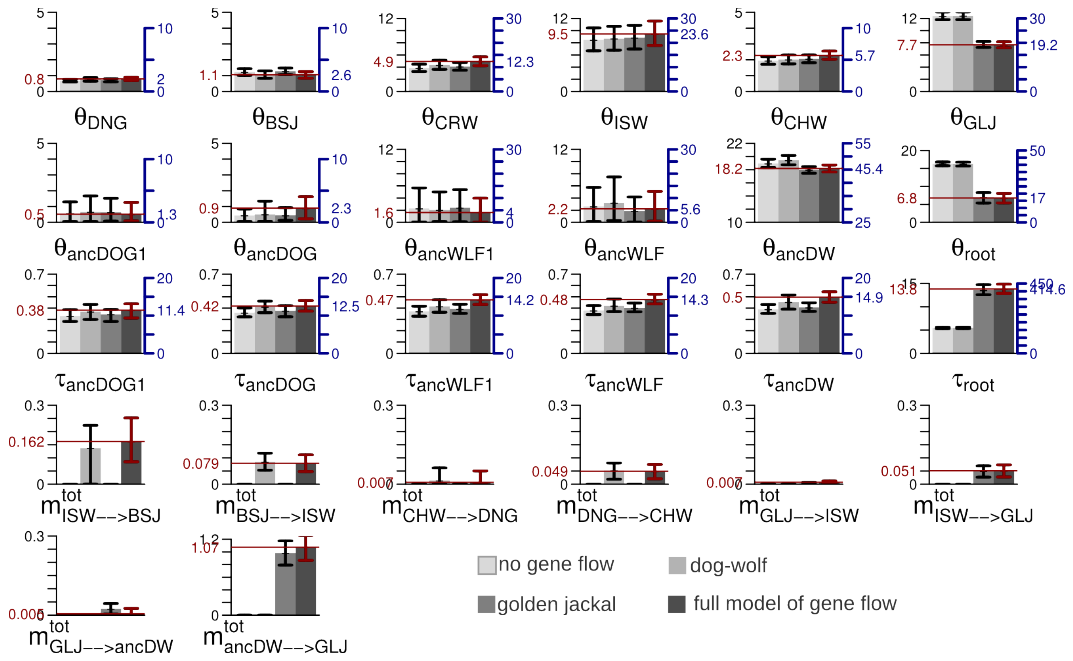

Figure S9.3.2: Parameter estimates under different scenarios of gene flow. Estimates and 95% Bayesian credible intervals for the 26 demographic parameters were obtained assuming four different scenarios of gene flow (left to right): (1) no gene flow; (2) gene flow between populations (ISW,BSJ) and between populations (CHW,DNG); (3) gene flow between populations (GLJ,ISW) and between populations (GLJ,ancDW); and (4) gene flow along all eight migration bands (highlighted in red). All four analyses were conducted on the set of 5,478 loci defined in Section S9.2.1 with the MCMC settings as described in Section S9.2.2. Raw estimates, scaled by mutation rate ($x10^4$), are shown (left axis) next to calibrated estimate (right axis). Calibrated divergence times are given in 1,000 years and calibrated population sizes are given in thousands of individuals (see Section S9.2.3 for details).

compared between sets of estimates obtained in four additional analyses: an analysis without any migration band, an analysis with the four bands corresponding to (ISW,BSJ) and (CHW,DNG) population pairs, an analysis with the four bands corresponding to (GLJ,ISW) and (GLJ,ancDW) population pairs, and an analysis with all eight bands. The four sets of parameter estimates are presented in Figure S9.3.2. Modeling gene flow with the golden jackal reduced the estimated effective size for the ancestral root population (N_{root}) from 41,000 to 17,000, and the effective size of the population ancestral to dogs and wolves (N_{ancDW}) from 47,000 to 45,000. The divergence times associated with these ancestral populations consequently increased from 163 thousand years ago (kya) to 415 kya (T_{root}) and from 11.7 kya to 13.1 kya (T_{ancDW}). Modeling gene flow between dogs and wolves had no significant effect on the ancestral effective population sizes, but it did result in an increase in the estimate of the dog-wolf divergence time (T_{ancDW}=13.6 kya). Our full model of gene flow with eight migration bands resulted in further increase of this divergence time to 14.9 kya.

S9.4 Main Set of Estimates for All Demographic Parameters

The main set of parameter estimates reported in our study is based on a single *G-PhoCS* analysis of the 16,434 neutral loci defined in Section S9.2.1, assuming the population phylogeny with eight migration bands shown in Fig. S9.1.1. Parameter estimates are described in Supplementary Table S12. See Section S9.2.3 for details on calibration of the raw parameter estimates. In the following sections we validate the robustness of this inferred demographic model to various factors:

1. In Section S9.5 we compare the demographic model inferred by *G-PhoCS* to the one implied by the ancestral effective population sizes inferred by the pairwise sequentially Markovian coalescent (PSMC) method of Li and Durbin [2] (see Text S8).
2. In Section S9.6 we examine several other plausible topologies for the population phylogeny associated with alternative hypotheses for dog domestication.
3. In Section S9.7 we demonstrate the robustness of our estimates to assumptions made in the construction of the collection of neutral loci we used in the analysis.

S9.5 Comparison with Estimates from PSMC Analysis

The demographic history of dogs and wolves as inferred by *G-PhoCS* is fairly consistent with the history inferred by separately analyzing the six diploid genomes using the pairwise sequentially Markovian coalescent (PSMC) method of Li and Durbin [2] (see Text S8). Both analyses infer similar ancestral population sizes, with a parallel decline in sizes observed for dogs as well as wolves. However, whereas *G-PhoCS* infers that dogs and wolves diverged roughly 15 kya, the ancestral effective population sizes inferred from the two dog genomes by PSMC diverge from those inferred from the three wolf genomes at a time point roughly 40-50 kya. Li and Durbin note that their method is likely to interpret abrupt changes in population sizes as gradual changes that started earlier in time. Thus, if dogs and wolves experienced strong population bottlenecks, their inferred ancestral sizes would appear to diverge before the ancestral populations diverged. We confirmed this observation by showing that PSMC produces a similar pattern of early divergence when run on data simulated according to the demographic model inferred by *G-PhoCS* (Supplementary Fig. S2; see also subsection S8.2.2 in Text S8).

As additional validation of the more recent divergence inferred by *G-PhoCS*, we conducted the reciprocal experiment in which *G-PhoCS* was run on data simulated according to a demographic model implied by the PSMC estimates. In these simulations, we assumed the population phylogeny inferred by neighbor joining (Fig. 4) without the boxer population (since the haploid boxer genome was not analyzed by PSMC). Divergence times (in years) were set to T_{ancDOG} = 13,000, T_{ancWLF} = T_{ancWLF1} = 42,800, and T_{ancDW} = 47,500, according to approximate times associated with divergence of the ancestral effective population sizes inferred by PSMC. We simulated gradual change in effective population size, as inferred by PSMC; for the current populations BSJ, DNG, ISW, CRW, CHW, and GLJ we used ancestral sizes inferred for the appropriate genome, for the ancestral population ancDOG we used ancestral sizes inferred from the basenji genome, and for the ancestral populations ancWLF1, ancWLF, ancDW, and root we used ancestral sizes inferred from the genome of the Israeli wolf. All parameters were scaled assuming an average mutation rate of 1.0×10^{-8} mutations per site per generation, and an average generation time of 3 years (see Section S9.2.3).

In order to examine the potential effects of intra-locus recombination on our estimates, we simulated data under three levels of recombination: r = 0.0 cM/Mb, r = 0.25cM/Mb, and r = 0.92 cM/Mb. The lower recombination rate (r = 0.25 cM/Mb) was based on the estimate from the PSMC analysis (see Text S8), and the higher rate (r = 0.92 cM/Mb) was based on the mean recombination rate estimated in the dog genome from a linkage map generated using microsatellites [3]. We generated four replicate data sets for each recombination rate using the MS simulation software [4] , each with 5,000 alignments of length 1 kb, and ran *G-PhoCS* on these data sets using the same settings as in our main analysis (including migration bands). Estimates of divergence times were highly concordant with the values used in generation of the data across the twelve data sets, regardless of recombination. Recombination appears mostly to influence the estimates for the effective population size and divregence time at the root (N_{root} and T_{root}), due to the recombination events that occurred since divergence from golden jackal.

The parameter estimates obtained on these 4x3=12 simulated data sets are described in Supplementary Figure S3. This experiment shows that *G-PhoCS* accurately infers population divergence times in demographic histories with gradual changes in ancestral population sizes, even in the presence of a small amount of intra-locus recombination. Because the divergence times *G-PhoCS* infered from real data were very different from the ones it infered from data simulated under the PSMC-based model, we conclude that the PSMC-based model with deep divergence does not fit the data. Additionally, the reciprocal experiment where PSMC was run on data generated according to the demographic model inferred by *G-PhoCS* (Supplementary Fig. S2) suggests that the deep divergences observed in the PSMC estimates are consistent with the model inferred by *G-PhoCS.*

S9.6 Alternative Topologies of the Population Phylogeny

Our demographic analysis is conditioned on a given topology for the population phylogeny. In our main analysis, we assumed the topology of the neighbor joining tree (Fig. 4). This tree describes dogs and wolves as evolving in two separate clades. We examined plausible alternative topologies in two series of analysis, to ensure that our estimates were not strongly affected by

our assumptions on the tree topology.

S9.6.1 Regional Origin

One alternative scenario for the joint history of dogs and wolves is that dogs were domesticated separately in different geographic regions. To test this hypothesis, we considered three alternative topologies for the population phylogeny, in which each geographic region–Middle East (MEA), East Asia (EAS), and Europe (EUR)–corresponds to an ancestral population with two daughter populations: dog and wolf (Supplementary Fig. S4A). Each of the three alternative topologies is determined by the order of geographic divergence events. We conducted demographic inference conditioned on each of these three topologies, once assuming no gene flow between populations, and once with 16 migration bands: all bands between sampled dog populations, all bands between sampled wolf populations, and bands between GLJ and ISW and the population ancestral to all dogs and wolves (ancDW).

When no post-divergence gene flow is allowed in the model, the estimated divergence times decrease to levels lower than our original estimate of the divergence between bansenji and boxer (Supplementary Fig. S4B; T_{ancDW} = 9,000 (8,600-10,200) across the three runs). This likely reflects poor fit of these models to the data, as a consequence of the similarity between the dog genomes. When we introduced post-divergence gene flow between dogs and between wolves into the model, the estimated divergence times increase significantly. However, migration rates were estimated to be very high, with total rates near 1.0 for the BSJ-to-BOX migration band, and total rates near 0.5 for the BSJ-to-DNG migration band. We conclude that in order to accommodate a hypothesis of separate regional domestication of dogs, there had to have been very high levels of post-divergence gene flow between dog (and wolf) populations from different geographic regions. This is in contrast to our default model with separate clades for dogs and wolves, which can be fit to data with considerably less post-divergence gene flow.

S9.6.2 Alternative Origins for the Dog Clade

Another alternative is that dogs were domesticated once, and thus form a distinct clade in the phylogeny, but the origin of domestication is not the population ancestral to all wolves. Assuming the topology of the wolf subphylogney is ((ISW,CRW),CHW), there are five possible origins for the dog clade, corresponding to the five branches of that phylogeny (Supplementary Fig. S5A). We conducted demographic inference conditioned on each of the four alternative topologies with the eight migration bands assumed in our original analysis. Overall, estimates of all parameters were very similar to our original estimates (Supplementary Fig. S5B). In all five analyses, the difference between the three divergence times T_{ancWLF1}, T_{ancWLF}, and T_{ancDW} were very small, but they were markedly higher when the original topology was assumed: $|\Delta_\tau| = |\tau_{\text{ancDW}} - \tau_{\text{ancWLF}}|$ = 597 years (42–1,416) in our original analysis compared to $|\Delta_\tau|$= 81 years (0–643) across the other four analyses. We conclude that the data does not significantly support a particular origin for dogs, but regardless of our assumptions on the identity of the ancestral lineage from which dogs were domesticated, this lineage diverged from other wolf lineages considered in this study at roughly the same time they diverged from each other (14–15 kya).

S9.7 Alternative Sets of Neutral Loci

The parameter estimates obtained by *G-PhoCS* depend on the collection of neutral loci used in the analysis (see Section S9.2.1). Certain assumptions made in the construction of these loci determined locus length, distance from coding exons, and even random subsetting, all of which can potentially influence the resulting estimates. *G-PhoCS* has been shown by Gronau *et al.* [1] to be robust to these factors in the analysis of individual human genomes. In this section we present similar validation experiments conducted on the individual canid genomes analyzed in this study.

S9.7.1 Subsetting of Loci

We compared parameter values inferred for the full set of 16,434 loci to values inferred for each of three disjoint equally-sized subsets of that set, obtained by selecting every third locus in the original set. Estimates of all parameters show high levels of agreement across these four analyses (Fig. S9.7.1). As expected, Bayesian credible intervals were smaller when all 16,434 loci were analyzed.

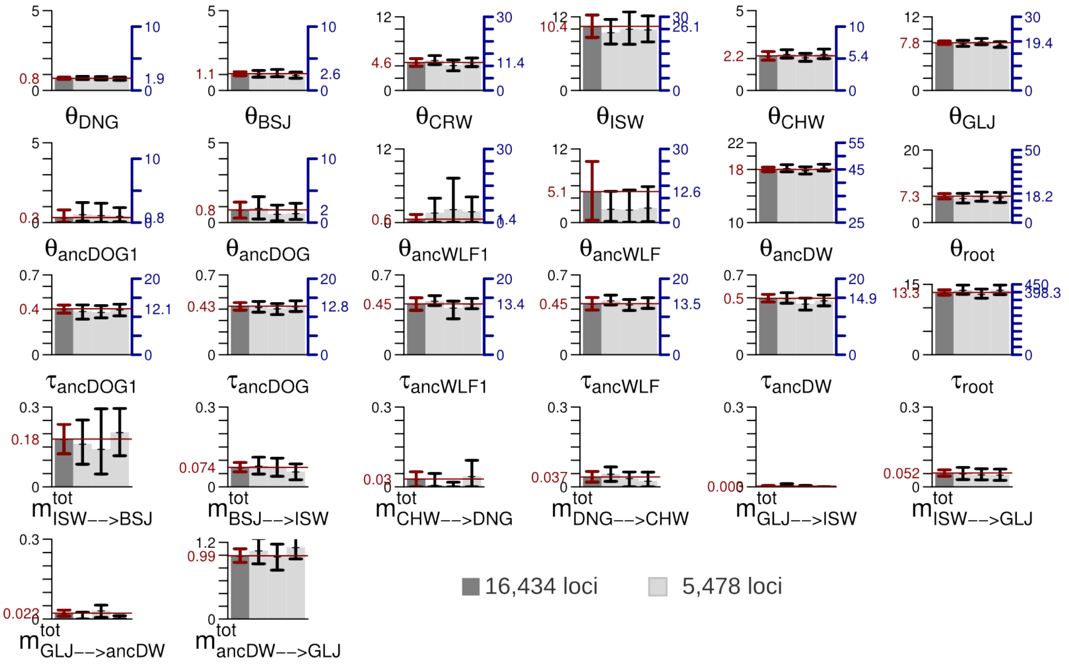

Figure S9.7.1: Parameter estimates for different sets of neutral loci. Estimates and 95% Bayesian credible intervals for the 26 demographic parameters were obtained using four different sets of neutral loci: the full set of 16,434 loci (dark gray; see Section S9.2.1), and three equally-sized disjoint subsets of that set (light gray) obtained by selecting every third locus in the original set. Raw estimates, scaled by mutation rate ($\times 10^4$), are shown (left axis) next to calibrated estimate (right axis) (see Section S9.2.3 for details on calibration).

S9.7.2 Locus Length and Intra-locus Recombination

A locus size of 1 kb was chosen for our main analysis in order to ensure small amounts of intra-locus recombination, while maintaining a reasonable number of informative sites within each locus. In order to validate the robustness of our parameter estimates for the potential effects of intra-locus recombination, we redid the analysis for different sets of loci with different lengths. To this end, we computed a set of 7,297 neutral loci, 2 kb long, from our collection of filtered neutral sites (see Table S9.1.1) with an inter-locus distance of at least 30 kb. By partitioning each locus in this set to two non-overlapping blocks of size 1kb, we constructed two non-overlapping collections of 1 kb loci, and by further partitioning each 1 kb locus to two 500 bp blocks, we constructed four collections of 500 bp loci.

We analyzed each of these seven different collections of 7,297 loci using *G-PhoCS* with the population phylogeny shown in Fig. S9.1.1, including eight migration bands. Overall, estimates obtained from loci of length 1 kb were very similar to the ones obtained from the shorter 500 bp loci (Fig. S9.7.2). Importantly, estimates of migration rates along the eight migration bands did not appear to be substantially affected by locus length. Recombination events that occurred since divergence of dogs and wolves within the analyzed loci would tend to increase the estimated divergence time (T_{ancDW}). However, estimates of T_{ancDW} obtained from the 1 kb loci and 500 bp loci were highly concordant with our original estimate of T_{ancDW}= 14.9 kya (13.9–15.9 kya). On the other hand, the estimate obtained from the collection of 2 kb loci increased to 21 kya (19–23 kya), most likely owing to a substantial increase in the number of intra-locus recombination events in these longer loci.

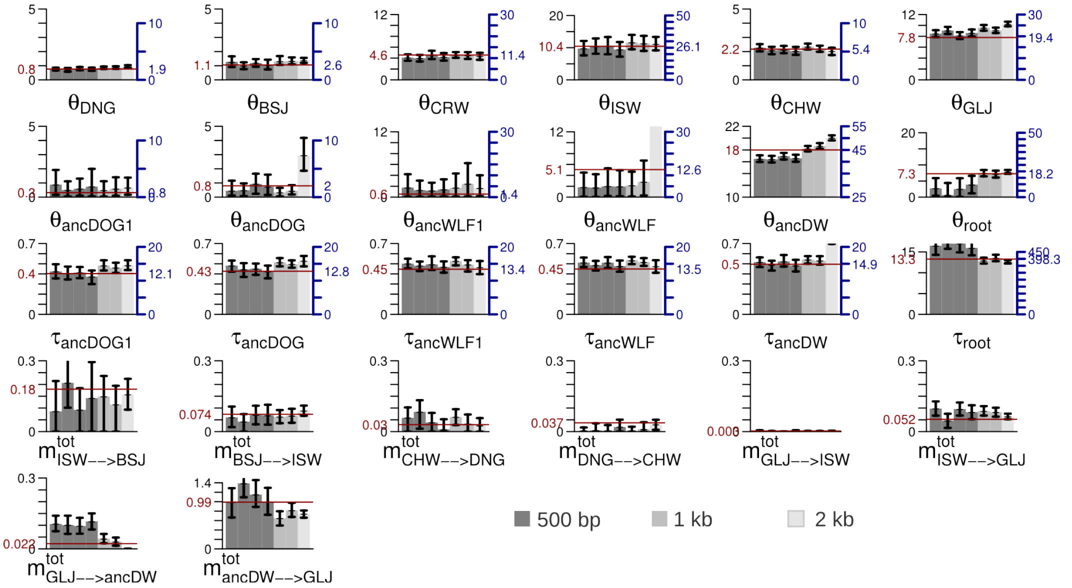

Figure S9.7.2: Effect of intra-locus recombination on parameter estimates. Estimates and 95% Bayesian credible intervals for the 26 demographic parameters were obtained using seven different sets of neutral loci at different lengths: 500 bp, 1 kb and 2 kb. Each data set contains 7,297 neutral loci. The horizontal red line marks the estimate obtained by our main analysis of 16,434 loci of length 1 kb. Raw estimates, scaled by mutation rate ($\times 10^4$), are shown (left axis) next to calibrated estimate (right axis) (see Section S9.2.3 for details on calibration).

S9.7.3 Distance from Coding Exons and Effect of Selection at Linked Sites

Another factor that could potentially affect our estimates is natural selection acting on linked sites (e.g., background selection or hitchhiking), which is known to reduce levels of genomic diversity around genes [5]. For this reason, we chose our neutral loci in regions that are located at least 10 kb away from the closest gene. In order to ensure that this approach was sufficiently conservative, we computed alternative sets of loci using different thresholds for this distance: 1, 2, 5, 20, 50, and 100 kb. We applied the same pipeline described in Section S9.2.1 to compute the alternative sets of loci (using alternative thresholds for distances to genes). We subsampled a collection of 5,478 loci from each set, to match the number of loci in our original analysis, and ran *G-PhoCS* on each of these six alternative data sets (Fig. S9.7.3). None of the parameters showed a strong trend in estimated values as a function of distance from genes, implying that our analysis is not sensitive to selection at linked sites.

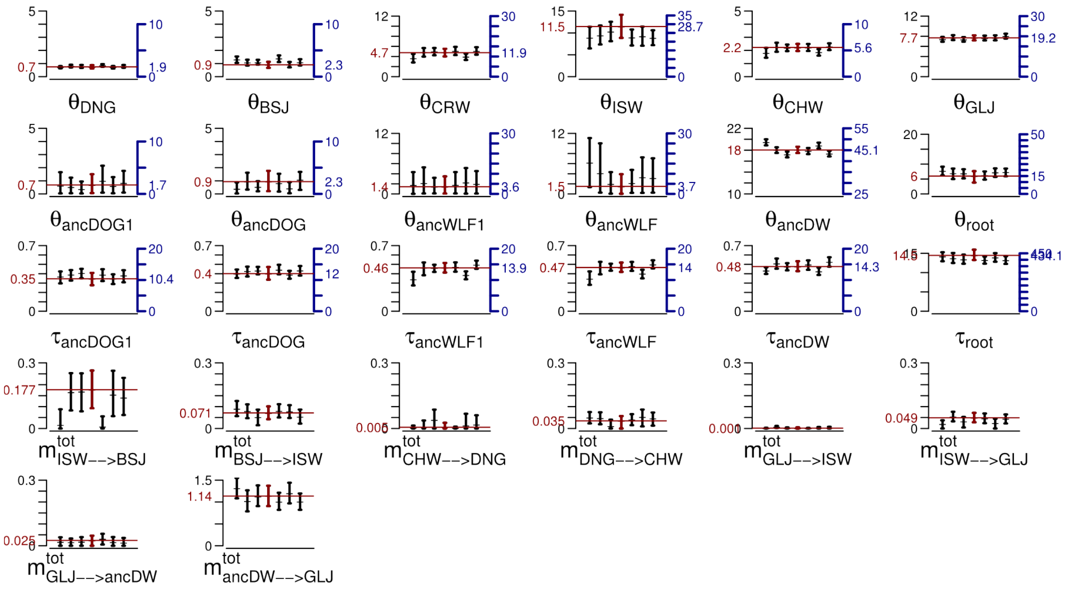

Figure S9.7.3: Effect of distance from genes on parameter estimates. Estimates and 95% Bayesian credible intervals for the 26 demographic parameters were obtained using seven different sets of neutral loci computed using different thresholds for distance from coding exons (left to right; in kb): 1, 2, 5, 10, 20, 50, 100. Each data set contains 5,478 loci of length 1 kb. The horizontal red line marks the estimate obtained using the default threshold of 10 kb. Raw estimates, scaled by mutation rate ($x10^4$), are shown (left axis) next to calibrated estimate (right axis) (see Section S9.2.3 for details on calibration).

References

1. Gronau I, Hubisz MJ, Gulko B, Danko CG, Siepel A (2011) Bayesian inference of ancient human demography from individual genome sequences. Nat Genet 43(10):1031–1034.

2. Li H, Durbin R (2011) Inference of human population history from individual whole-genome sequences. Nature 475(7357):493–496.

3. Wong AK, Ruhe AL, Dumont BL, Robertson KR, Guerrero G, et al. (2010) A comprehensive linkage map of the dog genome. Genetics 184(2):595–605..

4. Hudson R (2002) Generating samples under a Wright-Fisher neutral model of genetic variation. Bioinformatics 18(2) 337–338.

5. McVicker G, Gordon D, Davis C, Green P (2009) Widespread genomic signatures of natural selection in hominid evolution. PLoS Genet 5(5)
