# Supplementary material for: Genome Sequencing Highlights the Dynamic Early History of Dogs
Source: PLoS Genet. 2014 Jan 16;10(1):e1004016. doi: 10.1371/journal.pgen.1004016 (PMC3894170; doi:10.1371/journal.pgen.1004016)
Supplement: Text S10 — Copy number status of the amylase gene (AMY2B) on CFA6 in 12 dog breeds. (PDF) [file pgen.1004016.s029.pdf]

## S10 Copy Number Status of the Amylase Gene (*AMY2B*) on CFA6 in 12 Dog Breeds

***Holly Beale[1], Elaine Ostrander[1]***

***[1]National Institutes of Health***
*Cancer Genetics Branch*
*National Human Genome Research Institute*
*Bethesda, Maryland, United States of America.*

Copy number at the variant near AMY2B on Chr6 was measured in whole genome sequencing of 12 dogs, each belonging to a different breed, in a manner similar to the measurement in the wild canids. Specifically, a 7Kb CNV was identified with the approximate CanFam 3.1 coordinates 46,948,800- 46,956,325. Two of the exons of the human AMY2B transcript ENST00000361355 are syntenic to the variant (Table S10.1). Depth plots suggest that the copy is not continuous across the variant, and consequently a subregion spanning the syntenic exons was selected for the purposes of measuring copy number (Figure S6). Copy number was calculated as the average fold increase of sequence depth in the selected region divided by the average read depth (5.9-11.4x) in the surrounding 1 Mb. The expected average value for diploid single-copy regions is approximately 2.

The copy counts are consistent with the AMY2B results reported in Axelsson et al. [1]. All dogs have a duplication (Figure S6). The lowest copy number in these 12 samples is 3 or 4 copies (Husky). The copy number in other dogs ranges from 8 (Beagle) to 30 (Saluki).

**Table S10.1.** Syntenic positions of Human *AMY2B* exons on Chr6 (CanFam3.1 coordinates).

| ID | Start | End |
|---|---|---|
| ENSE00001940684 | 4,6954,362 | 46,954,570 |
| ENSE00001712096 | 46,955,754 | 46,955,872 |

### References

1. Axelsson E, Ratnakumar A, Arendt M-J, Maqbool K, Webster MT, et al. (2013) The genomic signature of dog domestication reveals adaptation to a starch-rich diet. Nature 495: 360-364.
