# Supplementary material for: Genome Sequencing Highlights the Dynamic Early History of Dogs
Source: PLoS Genet. 2014 Jan 16;10(1):e1004016. doi: 10.1371/journal.pgen.1004016 (PMC3894170; doi:10.1371/journal.pgen.1004016)
Supplement: Text S11 — Comparison of golden jackal sample to jackals and wolves. (PDF) [file pgen.1004016.s030.pdf]

S11 Comparison of Golden Jackal Sample to Jackals and Wolves

Carles Vilà[1], Robert K Wayne[2]

[1]Estación Biológica de Doñana
Department of Integrative Ecology
Sevilla, Spain

[2]University of California, Los Angeles
Department of Ecology and Evolutionary Biology
Los Angeles, California, United States of America

Recent reports have indicated that some North African golden jackals possess gray wolf-like mitochondrial DNA sequences [1,2]. The authors have suggested that those individuals may represent a previously undescribed gray wolf lineage present in North Africa, thus supporting previous claims based on the usual morphology and size of some canid specimens from this region [3]. Conceivably, the recent divergence estimated with *G-PhoCS* between golden jackals and gray wolves/dogs compared to previous estimates [4] could be due to a presence of wolf-like individuals in Israel rather than the more basal golden jackal lineage [5]. However, the mitochondrial DNA of the sequenced jackal was not wolf-like (unpublished results) and critically, analysis of previously published SNP data suggest Israeli golden jackals, including the specimen used in this paper, represent a lineage distinct from those in North Africa (Figure S11.1). Also, an analysis of 25 exonic sequences (16,180 bp) across a large panel of over 150 jackals from outside Africa did not indicate close proximity to the gray wolf / dog clade (Koepfli *et al.* unpublished data).

The divergence time of wolves and golden jackals has been estimated as about 1.5 million years from fossil data [4]. The molecular data presented here and results in preparation suggest a much more recent divergence time, indicating that fossil remains thought to be golden jackals are not directly ancestral to living forms. The difficult of deducing affinities of a generalized canid such as the golden jackal are exemplified by the problems in assigning recent specimens to one species or another [3].

Methods

<u>Allele Sharing Distance/Neighbor Joining Tree Reconstruction</u>

A subset of golden jackal samples from Kenya and Israel were previously genotyped using the Affymetrix Canine Mapping SNP Array 2.0 (127K SNPs) [6,7] and Illumina CanineHD array (170K SNPs), along with a number of other species in the *Canis* genus. To provide additional resolution and insight into the phylogenetic relationships of the African and Middle Eastern golden jackals with other canids, a neighbor-joining (NJ) tree based on allele-sharing distances was generated with a subset of SNPs from a panel of two Kenyan golden jackals, two Israeli golden jackals representing both mitochondrial haplotype clades (Cau-like and Clu-like), two ancient and two modern dog breeds, gray wolves from the Middle East, Asia, Europe and North America, Coyotes (*C. latrans*), Ethiopian wolves (*C. simensis*), and a Black-backed jackal (*C. adjustus*) and Side-striped

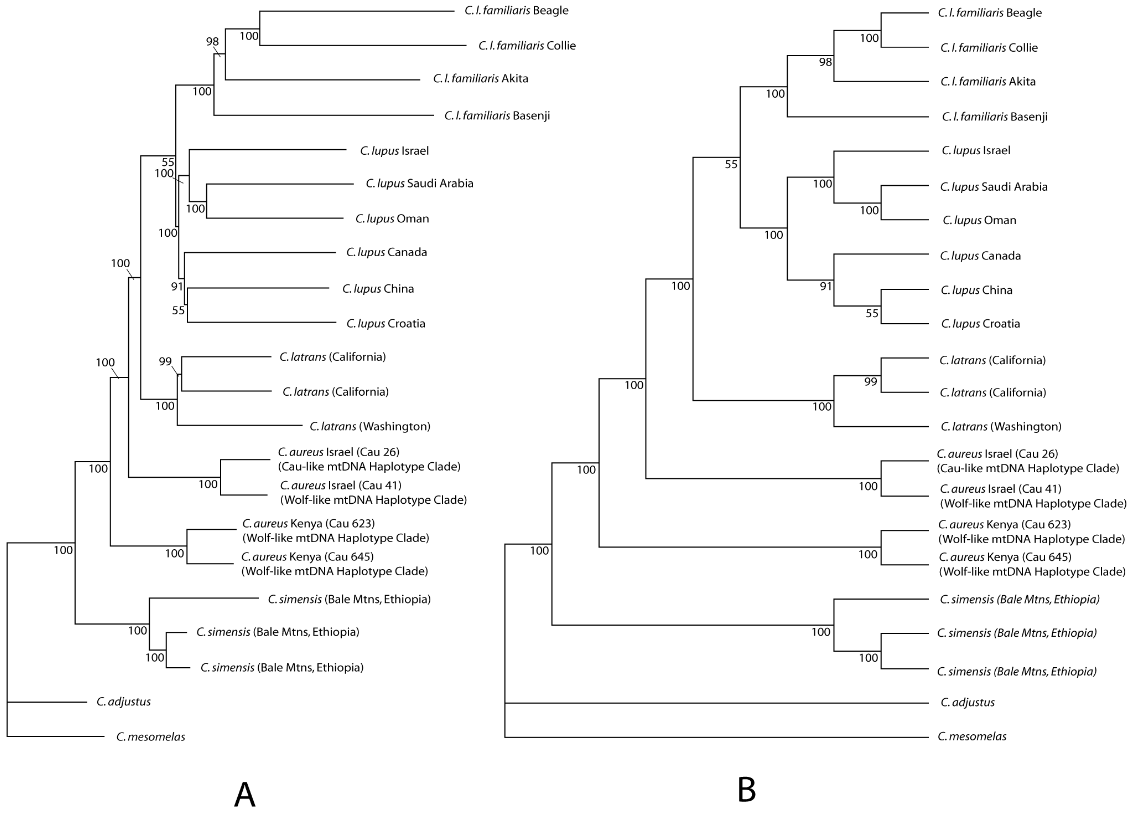

Figure S11.1. Neighbor-joining allele-sharing distance based phylogram (A) and cladogram (B) utilizing the 14,695 SNP dataset from canine SNP array assays. Bootstrap support based on 1,000 replicates is shown. Sample numbers and mtDNA haplotype clades for golden jackals are indicated for comparison with other analysis results.

jackal (*C. mesomelas*). We applied allele conversions to a subset of 14,695 nuclear SNPs distributed across the genome to generate a combined dataset, based on a comparative analysis of the 52,329 SNPs that overlap between the two different arrays (kindly provided by Suiyuan Zhang of NHGRI).

The allele-sharing distance was calculated as one minus the proportion of alleles shared for the 14,695 SNP panel, using the program MICROSAT [8] with 1,000 bootstrap replicates. Neighbor joining trees for each replicate of the resulting pairwise matrices of allele sharing distance were calculated using the program NEIGHBOR and a consensus tree was generated using the majority rule option in the program CONSENSE, both from the PHYLIP package [9]. The resulting tree was visualized as unrooted (Figure S11.1) using DENDROSCOPE [10].

References

1. Rueness EK, Asmyhr MG, Sillero-Zubiri C, Macdonald DW, Bekele A, et al. (2011) The Cryptic African Wolf: Canis aureus lupaster Is Not a Golden Jackal and Is Not Endemic to Egypt. PLoS ONE 6: e16385.
2. Gaubert P, Bloch C, Benyacoub S, Abdelhamid A, Pagani P, et al. (2012) Reviving the African Wolf Canis lupus lupaster in North and West Africa: A Mitochondrial Lineage Ranging More than 6,000 km Wide. PLoS ONE 7: e42740.
3. Ferguson WW (1981) The Systematic Position of Canis-Aureus-Lupaster (Carnivora, Canidae) and the Occurrence of Canis-Lupus in North-Africa, Egypt and Sinai. Mammalia 45: 459-465.
4. Perini FA, Russo CAM, Schrago CG (2010) The evolution of South American endemic canids: a history of rapid diversification and morphological parallelism. J Evol Biol 23: 311-322.
5. Lindblad-Toh K, Wade CM, Mikkelsen TS, Karlsson EK, Jaffe DB, et al. (2005) Genome sequence, comparative analysis and haplotype structure of the domestic dog. Nature 438: 803-819.
6. vonHoldt BM, Pollinger JP, Earl DA, Knowles JC, Boyko AR, et al. (2011) A genome-wide perspective on the evolutionary history of enigmatic wolf-like canids. Genome Res 21: 1294-1305.
7. vonHoldt BM, Pollinger JP, Lohmueller KE, Han EJ, Parker HG, et al. (2010) Genome-wide SNP and haplotype analyses reveal a rich history underlying dog domestication. Nature 464: 898-902.
8. Minch E, Ruiz-Linares A, Goldstein D, Feldman M, Cavalli-Sforza L (2009) MICROSAT: A computer program for calculating various statistics on microsatellite allele data. Palo Alto, CA: Stanford University. pp. MICROSAT: A computer program for calculating various statistics on microsatellite allele data.
9. Felsenstein J (1993) PHYLIP (Phylogency Inference Package) version 3.5c. Seattle, WA: Department of Genetics, University of Washington.
10. Huson DH, Richter DC, Rausch C, Dezulian T, Franz M, et al. (2007) Dendroscope: An interactive viewer for large phylogenetic trees. BMC Bioinformatics 8: 460.
